# Metal-free alkene oxy- and amino-perfluoroalkylations *via* carbocation formation by using perfluoro acid anhydrides: unique reactivity between styrenes and perfluoro diacyl peroxides†
†Electronic supplementary information (ESI) available: General experimental procedures, experimental method, compound characterization, and NMR spectroscopic data. See DOI: 10.1039/c8sc02547a


**DOI:** 10.1039/c8sc02547a

**Published:** 2018-08-01

**Authors:** Elena Valverde, Shintaro Kawamura, Daisuke Sekine, Mikiko Sodeoka

**Affiliations:** a Synthetic Organic Chemistry Laboratory , RIKEN Cluster for Pioneering Research , 2-1 Hirosawa , Wako , Saitama 351-0198 , Japan . Email: sodeoka@riken.jp; b RIKEN Center for Sustainable Resource Science , 2-1 Hirosawa , Wako , Saitama 351-0198 , Japan

## Abstract

We present a strategy for metal-free, alkene difunctionalization-type, oxy- and amino-perfluoroalkylations, using perfluoro acid anhydrides as practical and user-friendly perfluoroalkyl sources. This method provides efficient access to oxy-perfluoroalkylation products *via* carbocation formation due to the unique reactivity between styrenes and bis(perfluoroacyl) peroxides generated *in situ* from perfluoro acid anhydrides. This reaction is also applicable to metal-free intramolecular amino-perfluoroalkylation of styrenes bearing a pendant amino group. Synthetic utility of the oxy-trifluoromethylation products was confirmed by demonstrating derivatization *via* hydrolysis, elimination, and acid-catalyzed substitution with carbon nucleophiles. The mechanism of the carbocation formation was investigated experimentally and theoretically.

## Abstract 2

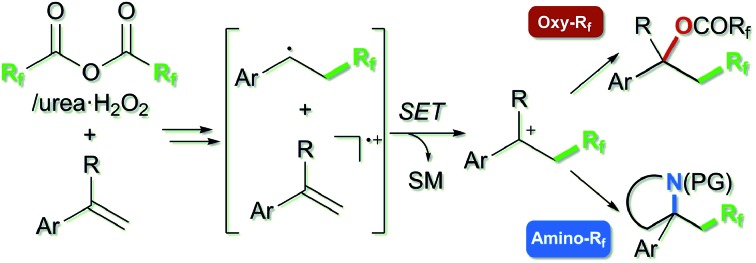
A practical metal-free perfluoroalkylation using acid anhydrides with unique reaction mode *via* carbocation has been developed.

## Introduction

**Scheme 1:**
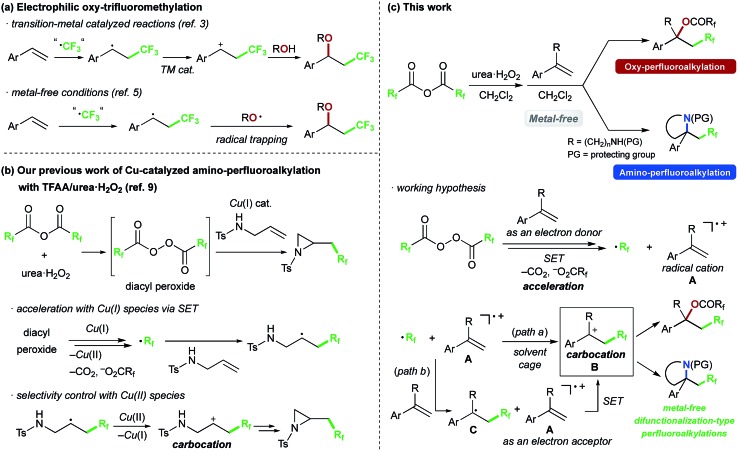
(a) Proposed mechanisms of previous oxy-trifluoromethylations, (b) our previous work on Cu-catalyzed amino-perfluoroalkylation with TFAA/urea·H_2_O_2_, and (c) metal-free difunctionalization-type perfluoroalkylation of styrenes by using perfluoro acid anhydrides (this work).

Introduction of perfluoroalkyl groups is an important strategy for modifying the properties of bioactive compounds, agrochemicals and functional materials.^1^ Various methods are available for C–CF_3_ bond formation to construct functionalized CF_3_-containing compounds, and alkene difunctionalization-type trifluoromethylation has recently attracted particular interest.^2^–^10^ Styrene derivatives are often used as substrates in these reactions because of their unique reactivity and the utility of the products as CF_3_-containing synthetic building blocks.^2^–^5^ For example, transition-metal-catalyzed intermolecular oxy-trifluoromethylation to form C–O bonds has been well studied (Scheme 1a).^3^ As pioneering works, Szabó3*a* and we3*b* independently reported Cu-catalyzed intermolecular oxy-trifluoromethylation of styrenes with Togni reagent in 2012. In this reaction, the trifluoromethyl group is introduced into the β-position, and then 2-iodobenzoate group derived from the Togni reagent is introduced at the benzylic position *via* carbocation intermediate formation with the aid of copper-catalyst. In contrast to transition-metal-catalyzed reactions, metal-free oxy-trifluoromethylations generally proceed *via* the following steps: (1) formation of a CF_3_ radical, (2) formation of an alkyl radical intermediate by reaction of the CF_3_ radical and alkene, (3) trapping with an O-radical species.^5^ In 1993, Uneyama reported an electrochemical reaction of butyl acrylate with trifluoroacetic acid (TFA) and O_2_ as the trapping agent, affording CF_3_-containing alcohol products.5*b* In 2011, Xiao found that *S*-(trifluoromethyl)diphenylsulfonium salt could react with styrenes under aerobic conditions to afford ketone products.5*c* A similar transformation was also achieved by using CF_3_SO_2_Na in the presence of O_2_, obtaining a mixture of ketone and alcohol products.5*e* Lei developed the reaction with CF_3_SO_2_Na in the presence of O_2_ with the aid of K_2_S_2_O_8_ or the combination of NMP/PPh_3_, to obtain the ketone or alcohol product selectively.5f,h Fu reported oxazoline forming-trifluoromethylation of allylamide with CF_3_SO_2_Na by using oxidant.5*g* In 2012, Studer reported an efficient TEMPONa-promoted oxy-trifluoromethylation with Togni reagent,5d,j in which Togni reagent was decomposed *via* single electron transfer with TEMPONa as an electron donor, affording CF_3_ and TEMPO radicals and eventually providing the CF_3_-containing TEMPO adduct by addition to the alkene. In 2015, Tan and Liu reported the metal-free oxy-trifluoromethylation using hydroxamic acids, affording products containing an aminoxyl group.5*i* As regards metal-free reaction *via* a carbocation intermediate, Uneyama developed an electrochemical oxy-trifluoromethylation of butyl methacrylate with TFA and water as the oxygen nucleophile and solvent by careful tuning the current density to oxidize the radical intermediate, obtaining the alcohol product in up to 35% yield.5*a* Further, in 2016, Liu reported an amine-catalyzed intramolecular oxy-trifluoromethylation of alkenes bearing a 1,3-diaryl diketone group with Togni reagent, affording dihydrofuran products.5k,^6^

Recently, we have been interested in alkene perfluoroalkylation by using perfluoro acid anhydrides, which are convenient and practical perfluoroalkyl sources because of their low cost, ready availability and reasonable stability compared to conventional perfluoroalkylating reagents.^9^–^12^ We found that perfluoro diacyl peroxides prepared *in situ* from perfluoro acid anhydrides and urea·H_2_O_2_ showed excellent reactivity and selectivity in allylic perfluoroalkylation9*a* and in intramolecular amino-perfluoroalkylation9*b* of alkenes in the presence of Cu(i) salt as a catalyst. Our mechanistic studies of the amino-perfluoroalkylation indicated that it proceeds *via* (1) formation of a perfluoroalkyl radical (·R_f_) and Cu(ii) species from the peroxide and Cu(i) catalyst, (2) addition of the R_f_ radical to the double bond of the alkene, (3) oxidation of the resulting alkyl radical with Cu(ii) species to afford a carbocation intermediate with recovery of the Cu(i) species, and (4) nucleophilic cyclization (Scheme 1b). In the absence of copper catalyst, the reaction of alkenes gave complex mixtures. Exceptionally, reaction of alkenes bearing an aromatic ring at an appropriate position selectively generated perfluoroalkyl group-containing carbocycles, because the aromatic ring acted as a scavenger of the alkyl radical. We were interested in the unique reactivity of styrenes and radical cation species in perfluoroalkylation with the perfluoro acid anhydride/urea·H_2_O_2_ system (Scheme 1c), and postulated that the styrene substrate serves to control the reactivity and selectivity in the formation of the carbocation intermediate without transition-metal-catalyst;9*b**i.e.*, styrene serves as an electron donor to accelerate generation of the perfluoroalkyl radical *via* decomposition of the diacyl peroxide by SET. Then, addition of the perfluoroalkyl radical to the resulting radical cation **A** affords the carbocation **B** (path a). Another possibility is that the perfluoroalkyl radical reacts with another styrene molecule (having higher electron density compared to the radical cation **A**), and the resulting benzyl radical intermediate **C** is oxidized by the radical cation **A** as an electron acceptor to afford the same benzyl cation intermediate **B** (path b). In this work, we focused on this carbocation formation, as a key process in difunctionalization-type perfluoroalkylation, and aimed to develop metal-free oxy- and amino-perfluoroalkylations of styrene derivatives by using perfluoro acid anhydrides. We also carried out various derivatizations to confirm the synthetic potential of the products.

## Results and discussion

**Scheme 2:**
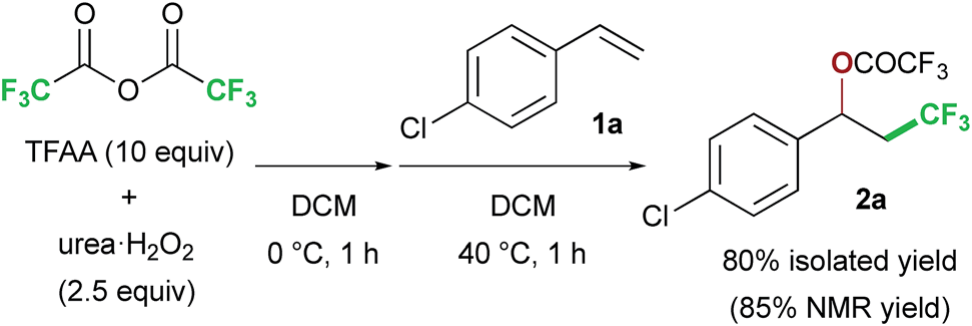
Metal-free oxy-trifluoromethylation of styrene **1a** with TFAA/urea·H_2_O_2_ under optimized conditions.

**Scheme 3:**
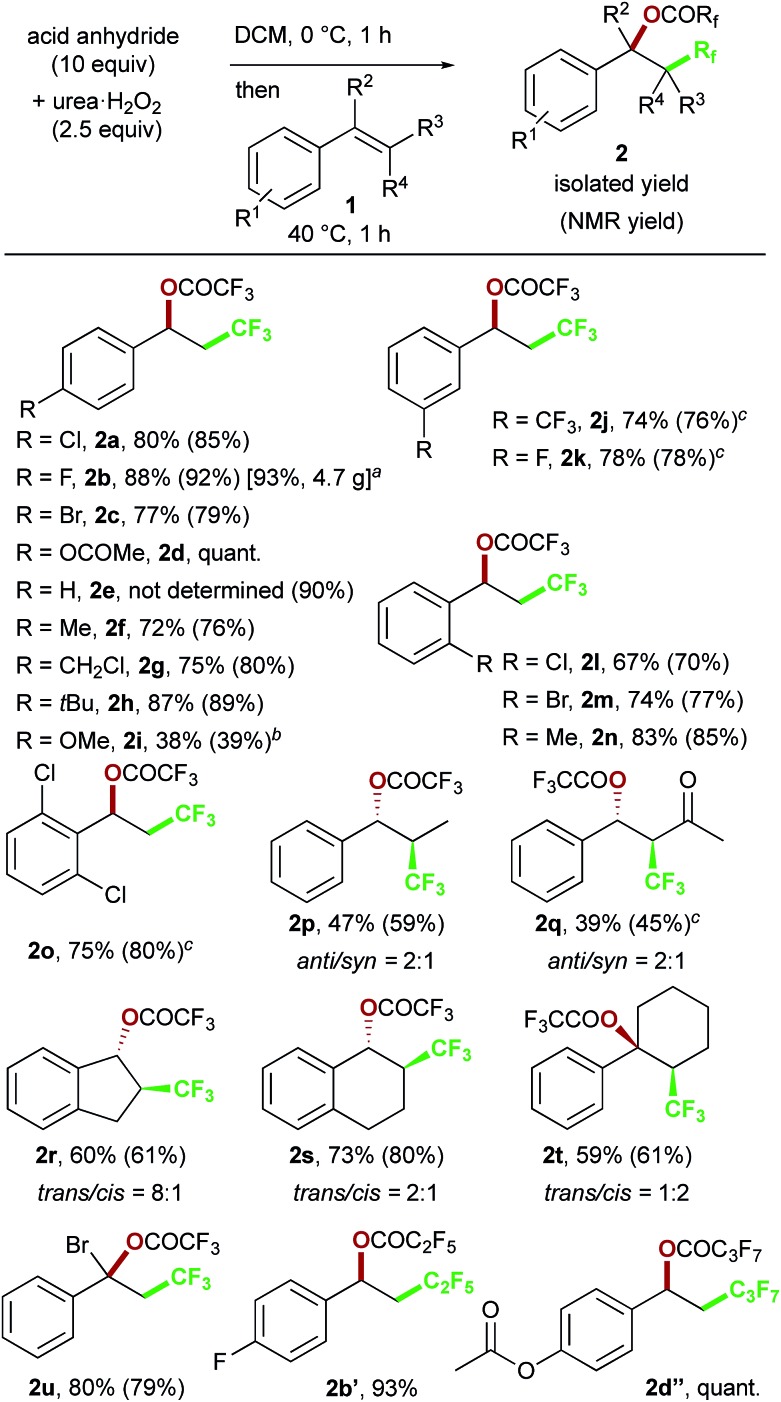
Substrate scope of the metal-free oxy-perfluoroalkylation reaction. ^a^Gram-scale synthesis using 2.0 g (16 mmol) of **1b**. ^b^Run at 0 °C for 10 min with Cs_2_CO_3_ (5 equiv.) as an additive. ^c^Run at 60 °C in 1,2-dichloroethane.

We chose commercially available 4-chlorostyrene **1a** as a model substrate to explore the reaction. To our delight, after *in situ* generation of bis(trifluoroacetyl)peroxide (BTFAP) from trifluoroacetic anhydride (TFAA) with urea·H_2_O_2_ in DCM at 0 °C for 1 h, reaction with **1a** at 40 °C for 1 h afforded the desired oxy-trifluoromethylation product **2a**. Careful tuning of the ratio of the reagents and the reaction temperature improved the yield.^13^ Finally, the reaction with TFAA (10 equiv.) and urea·H_2_O_2_ (2.5 equiv.) provided the corresponding oxy-trifluoromethylated product **2a** in 80% isolated yield (85% NMR yield) (Scheme 2).^14^ The scope of the optimized reaction conditions was then explored using a range of styrene-based substrates (Scheme 3). Various functional groups at the *para* position were tolerated and the corresponding oxy-trifluoromethylated products were formed efficiently (**2a–i**).^15^ The usefulness of the reaction was demonstrated in a gram-scale experiment with 4-fluorostyrene **1b**, which was transformed into the desired compound **2b** in 93% yield (4.7 g). *meta*- and *ortho*-substituted styrene substrates performed well in the oxy-trifluoromethylation reaction (**2j–n**), although higher temperatures were needed for *meta*-substituted substrate because of slow conversion compared to *para*- and *ortho*-substituted styrenes. A disubstituted styrene **1o** afforded the target compound **2o** in good yield. The generality of the reaction was also assessed with several internal alkenes, which afforded the corresponding difunctionalized products in moderate to good yields (**2p–s**). Quaternary carbon centres could be constructed successfully, and more complex compounds **2t** and **2u** were isolated in 59% and 80% yield, respectively. Finally, this metal-free procedure was applied to the oxy-perfluoroalkylation of styrene-based substrates with other perfluoro acid anhydrides, and the desired products **2b′** and **2d′′** were isolated in excellent yields.

**Scheme 4:**
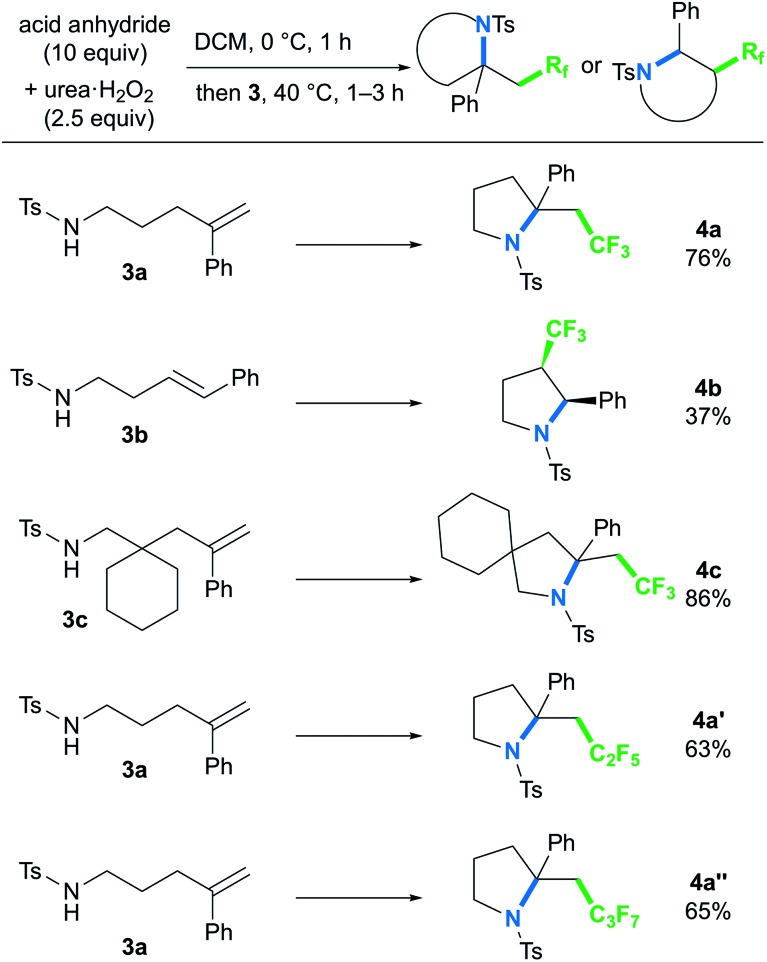
Metal-free amino-perfluoroalkylation reaction of alkenes.

To further explore the ability of the styryl functionality to provide the carbocation intermediate, we next investigated whether pyrrolidines could be obtained by intramolecular amino-perfluoroalkylation of styrene derivatives bearing a pendant amino group *via* nucleophilic cyclization, based on our previous work.9b,^16^ In contrast to metal-free oxy-perfluoroalkylation, metal-free amino-perfluoroalkylation has rarely been reported,5a,^8^ probably because of the lack of appropriate N-radical trapping agents. Thus, we examined the reaction of styryl group-containing aminoalkene **3a** with *in situ*-generated BTFAP under the optimal conditions for the oxy-trifluoromethylation (Scheme 4).^17^ As we had hoped, the amino-trifluoromethylation proceeded well to afford the corresponding CF_3_-containing pyrrolidine **4a** in 76% yield. This styrene-driven amino-trifluoromethylation was also applicable to internal alkene **3b**, which provided disubstituted pyrrolidine **4b** as a *syn*-diastereomer.^18^ In this reaction, the oxy-trifluoromethylation product was obtained as a by-product in 40% yield, and it was not converted to the amino-trifluoromethylation product **4b** even upon prolonged reaction. This observation suggested that this amino-trifluoromethylation does not proceed *via* nucleophilic substitution of the oxy-trifluoromethylation product under the conditions. Alkenyl amine **3c** featuring a 6-membered ring as a tethering group in the carbon chain was tolerated, and the spirocyclic product **4c** was formed in high yield. The use of acid anhydrides bearing longer perfluoroalkyl chains furnished C_2_F_5_- and C_3_F_7_-substituted pyrrolidines **4a′** and **4a′′** in good yields.

**Scheme 5:**
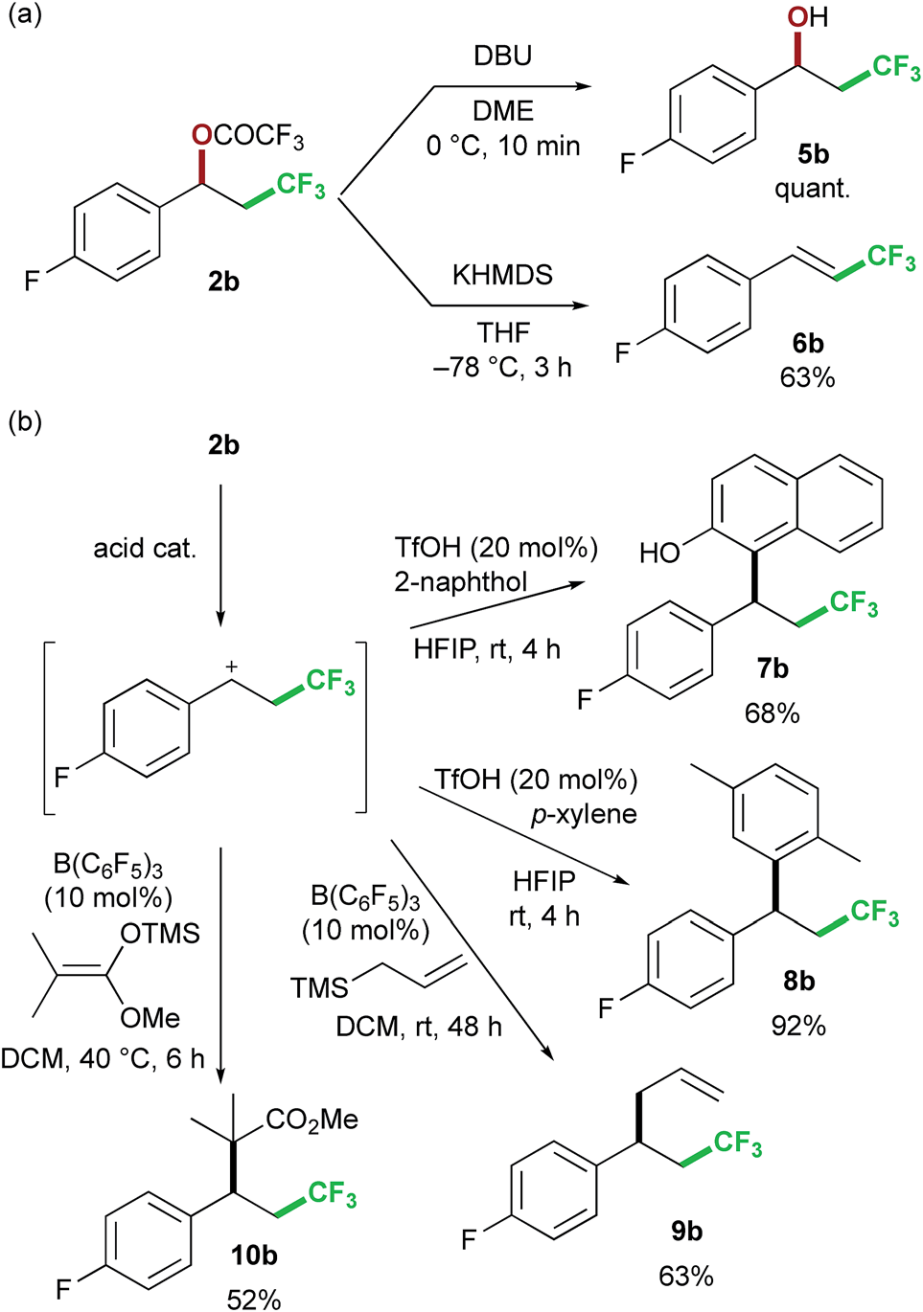
Examples of derivatization of benzyl trifluoroacetate **2b**: (a) hydrolysis and elimination reaction (b) intermolecular C–C bond formation reactions.

To further expand the chemical space of the perfluoroalkylated compounds, we focused on the reactivity of the perfluoroacetoxy group, as a labile functional group, in the oxy-trifluoromethylation products (Scheme 5). First, we examined the reaction of **2b**, as a representative substrate, with bases. When DBU was reacted with **2b** in DME, the alcohol **5b** was obtained in quantitative yield. On the other hand, KHMDS as the base was found to give the vinyl product **6b***via* elimination reaction. Next, we attempted to construct attractive carbon skeletons and examined S_N_1-type nucleophilic substitution reactions with various carbon nucleophiles in the presence of acid catalysts. The trifluoroacetoxy group was readily dissociated with triflic acid, and trapping of the resulting carbocation with arenes resulted in C–C bond formation to give **7b** and **8b**.^19^ In the presence of a catalytic amount of B(C_6_F_5_)_3_ to promote formation of the carbocation, allylation with allylsilane proceeded to afford **9b** in good yield.^20^ This approach was also applicable to the reaction with a ketene silyl acetal as a nucleophilic partner, providing **10b**. The presented procedures for the substitution reactions of the benzylic trifluoroacetoxy group provide rapid access to various perfluoroalkyl-group-containing molecules, which should be useful building blocks in organic synthesis.

**Scheme 6:**
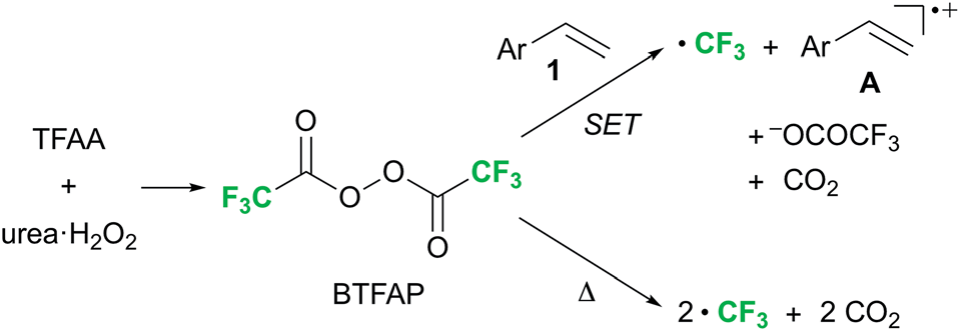
Possible pathway of decomposition of BTFAP.

**Scheme 7:**
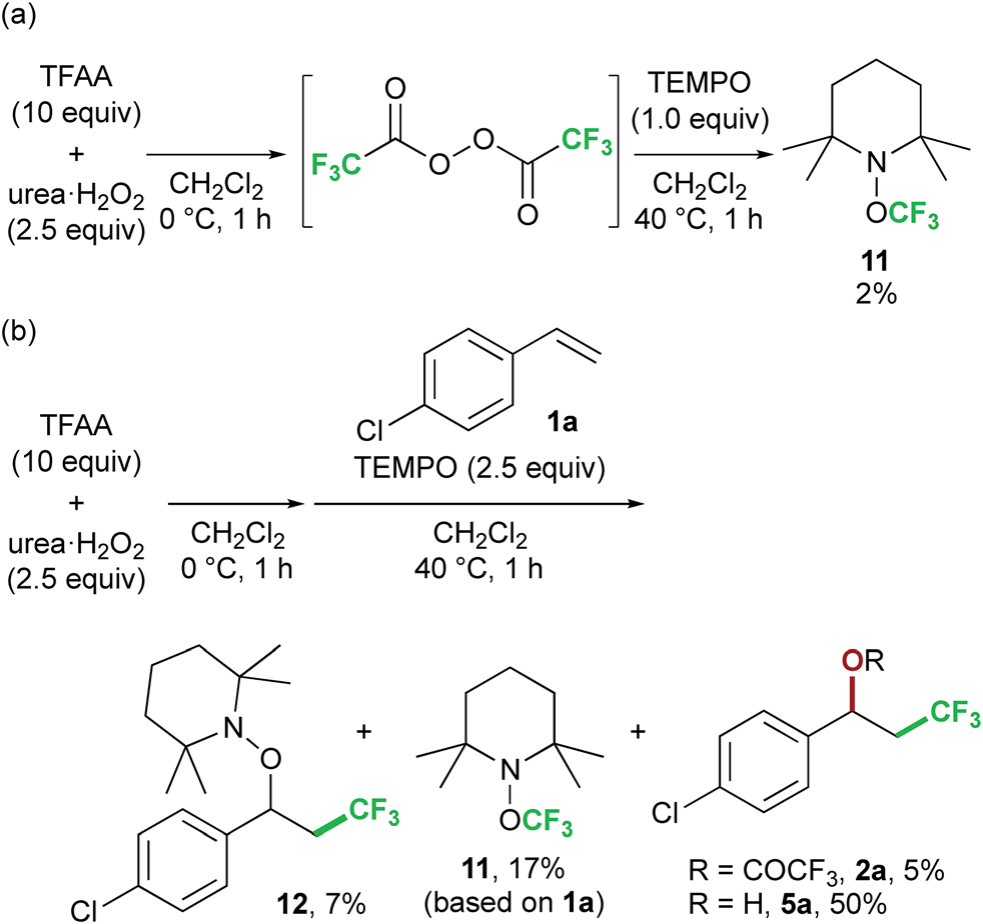
TEMPO trapping test: (a) reaction of TEMPO with BTFAP and (b) oxy-trifluoromethylation in the presence of TEMPO.

**Scheme 8:**
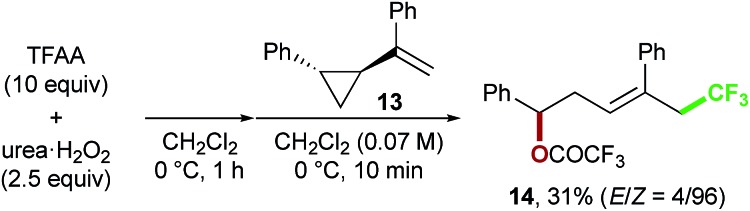
Radical probe test using **13**.

**Scheme 9:**
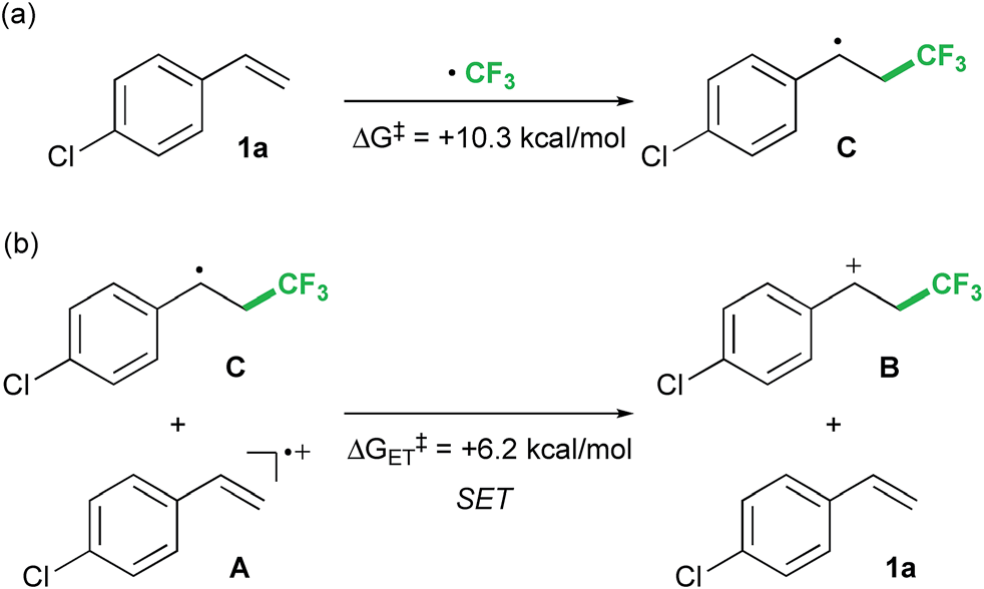
Activation energies of (a) addition of CF_3_ radical to **1a** and (b) SET between benzyl radical **C** and radical cation **A**.

Finally, we focused on the reaction mechanism of the perfluoroalkylation (Scheme 1c). The reaction starts with the generation of an electrophilic CF_3_ radical *via* decomposition of the diacyl peroxide, *i.e.* BTFAP which is formed *in situ* from TFAA and urea·H_2_O_2_. Decomposition of BTFAP would be triggered by SET with styrene as the electron donor and/or by heating (Scheme 6). To trace the decomposition, TEMPO instead of styrene was reacted with *in situ*-generated BTFAP (Scheme 7a). TEMPO-CF_3_ adduct **11** was obtained in only 2% yield, which suggested that thermal decomposition would be very slow under these reaction conditions.^21^ Furthermore, the decomposition could not be observed by ^19^F NMR monitoring of the peroxide in CD_2_Cl_2_ at 40 °C without any substrate.^13^ Yoshida similarly found that aromatic compounds such as benzene accelerate the decomposition of BTFAP by SET.11*b* The HOMO level of 4-chlorostyrene **1a** (–6.41 eV), used as the model substrate in this work, is higher than that of benzene (–7.09 eV).^13^ Accordingly, decomposition of BTFAP was concluded to be induced by SET with styrene under the present conditions at 40 °C, affording CF_3_ radical and radical cation **A**. Radical trapping with TEMPO under the optimized conditions afforded the CF_3_-containing TEMPO-benzyl adduct **12**, generated *via* the benzyl radical intermediate **C**, in 7% yield (Scheme 7b). In addition to **12**, TEMPO-CF_3_**11** was formed, together with a mixture of oxy-trifluoromethylation products **2a** and its hydrolysis product **5a**. Furthermore, a known radical probe alkene **13**, 1-phenyl-1-(*trans*-2-phenylcyclopropyl)ethane,8*b* was subjected to the reaction (Scheme 8). The corresponding ring-opening product **14** was formed *via* the radical intermediate as the major product, along with a complex mixture of other products.^13^ These results proved that the CF_3_ radical reacts with styrene **1a** (path b, Scheme 1c), although both path a and path b may be operated. Indeed, DFT calculation indicated that the activation energy of the reaction of the CF_3_ radical with **1a** is low (Δ*G*^‡^ = +10.3 kcal mol^–1^, Scheme 9a). Next, we considered the oxidation step of the benzyl radical to the carbocation **B**. Comparison of the calculated LUMO levels of potential oxidants, BTFAP and radical cation **A**, indicated that radical cation **A** (–6.26 eV) has a lower LUMO level than that of BTFAP (–2.35 eV). In addition, the LUMO level of **A** was closer to that of Cu(ii)(O_2_CCF_3_)_2_ (–5.09 eV) which was reported to oxidize the radical intermediate in the amino-perfluoroalkylation reaction (Scheme 1b).9*b* Thus, radical cation **A** was considered to act as the oxidant, affording carbocation **B**, which leads to the desired products. The DFT calculated activation energy of oxidation of benzyl radical **C** with **A** was Δ*G*‡ET = +6.2 kcal mol^–1^ (Scheme 9b), which is much lower than that of the addition of CF_3_ radical to **1a** suggesting rapid conversion of the highly reactive benzyl radical **C** to the metastable benzyl cation intermediate **B**. These mechanistic studies supported our original hypothesis shown in Scheme 1c, in which substrate styrene itself acts as SET donor to trigger the perfluoroalkyl radical formation from the diacyl peroxide. The resulting perfluoroalkyl radical could react with styrene affording benzyl radical intermediate **C**, which is rapidly oxidized to the benzyl cation **B** by the radical cation **A**. The generated benzyl cation intermediate **B** is trapped by the perfluoro carboxylate anion or amine yielding the desired oxy- and amino-perfluoroalkylation products, **2** and **4**.

## Conclusions

We have developed a mild and efficient method for the metal-free oxy- and amino-perfluoroalkylation of styrenes *via* carbocation intermediates, using perfluoro acid anhydrides as inexpensive and practical perfluoroalkyl sources. The oxy-trifluoromethylation products were derivatized to a variety of CF_3_-containing unique molecules. We believe this method will prove useful in medicinal and agro-chemistry discovery programs. In addition, the unique reactivity between styrene and perfluoro diacyl peroxide may provide clues to design new reactions and catalysts in the future.

## Conflicts of interest

There are no conflicts to declare.

## Supplementary Material

Supplementary information
