# Supplementary material for: Metal-free alkene oxy- and amino-perfluoroalkylations via carbocation formation by using perfluoro acid anhydrides: unique reactivity between styrenes and perfluoro diacyl peroxides
Source: Chem Sci. 2018 Aug 1;9(35):7115–21. doi: 10.1039/c8sc02547a (PMC6137437; doi:10.1039/c8sc02547a)
Supplement: Supplementary file 1 [file SC-009-C8SC02547A-s001.pdf]

Electronic Supplementary Material (ESI) for Chemical Science.
This journal is © The Royal Society of Chemistry 2018

SUPPORTING INFORMATION

Metal-free alkene oxy- and amino-perfluoroalkylations via carbocation formation by using perfluoro acid anhydrides: unique reactivity between styrenes and perfluoro diacyl peroxides

Elena Valverde,[a] Shintaro Kawamura,[a,b] Daisuke Sekine,[a] and Mikiko Sodeoka*[,a,b]

[a]*Synthetic Organic Chemistry Laboratory, RIKEN Cluster for Pioneering Research, 2-1 Hirosawa, Wako, Saitama 351-0198, Japan*

[b]*RIKEN Center for Sustainable Resource Science, 2-1 Hirosawa, Wako, Saitama 351-0198, Japan*

1. General Experimental

General: Reactions were conducted in a dry vessel under a positive pressure of nitrogen gas by using a nitrogen-filled balloon. Analytical thin-layer chromatography (TLC) was performed on glass plates coated with 0.25 mm 230–400 mesh silica gel (Merck, Silica gel 60 F_{254}) containing a fluorescent indicator. Visualization was accomplished by means of ultraviolet irradiation at 254 nm and/or by spraying an ethanolic solution of 12-molybdo(VI)phosphoric acid as a developing agent. Flash column chromatography was performed using Silica gel N-60 (spherical, neutral, 40–50 μm, Kanto Chemical Co., Inc. (Kanto)) as described by Still *et al.*[1]

Instrumentation:

NMR analysis

NMR spectra were recorded at room temperature on a JEOL JNM-ECS-400 NMR spectrometer at 400 MHz for ^{1}H, 100 MHz for ^{13}C, and 376 MHz for ^{19}F. The proton chemical shift values are reported in parts per million (ppm, δ scale) downfield from tetramethylsilane and referenced to the proton resonance of $CHCl_3$ (δ 7.26). The carbon chemical shift values are reported in parts per million (ppm, δ scale) downfield from tetramethylsilane and referenced to the carbon resonance of $CDCl_3$ (δ 77.16). The fluorine chemical shift values are reported in parts per million (ppm, δ scale) with $CFCl_3$ (δ 0.00) as an external standard. *J* values are reported in hertz (Hz). The data are presented in the following order: chemical shift, multiplicity (s = singlet, d = doublet, t = triplet, q = quartet, m = multiplet and/or multiple resonances, and br = broad), coupling constant and signal area integration in natural numbers.

IR analysis

Infrared spectra were measured on a Thermo Nicolet iS5. Only diagnostic absorptions are listed.

HRMS analysis

ESI-MS spectra were measured on a Bruker micrOTOF-QII-RSL. The samples were diluted with MeOH for measurement. EI-MS was taken on a JEOL JMS-T100GCV gas chromatograph time-of-flight mass spectrometer. The samples were diluted with $CHCl_3$ for measurement.

Solvents: Anhydrous dichloromethane was purchased from Kanto Chemical Co., Inc.

Materials: Reagents were purchased from Wako Pure Chemical Industries, Ltd. Tokyo

[1] W. C. Still, M. Kahn, A. Mitra, *J. Org. Chem.,* 1978, **43**, 2923.

Chemical Industry Co., Ltd. and Sigma-Aldrich Inc. Known alkenes **3a**[2] and **12**[3] were prepared according to the cited literature.

2. Additional Results

Optimization of the reaction conditions

Table S1. Optimization of the conditions for the synthesis of 2a[a]

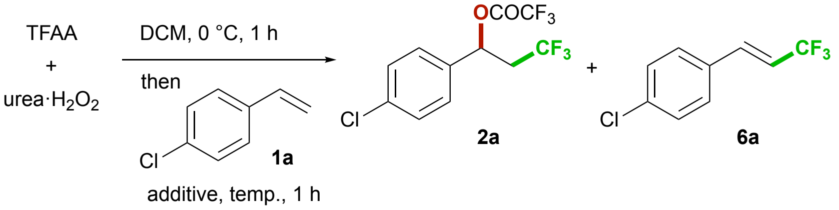

Entry	TFAA/urea·H_2O_2 (equiv.)	Temp. (°C)	Additive	Yield (%)[b] 2a	6a	Recovery of **1a** (%)[c]
1	4.0/1.2	0	–	0	0	94
2	4.0/1.2	25	–	38	3	32
3	4.0/1.2	40	–	60	5	14
4	6.0/1.2	40	–	44	4	15
5	8.0/2.2	40	–	74	4	0
6	8.0/2.2	40	TFA (0.4 equiv.)	75	4	0
7	8.0/2.2	40	TFA (1.0 equiv)	75	3	0
8	10/2.5	40	–	85(80)[d]	4	0
9	12/3.0	40	–	81	3	0
10	10/3.5	40	–	83	3	0

[a]The reactions were conducted on 0.20 mmol scale. [b]The yields were estimated by means of ^{19}F NMR analysis, with α,α,α-trifluorotoluene as an internal standard. [c]The recovery of **1a** was estimated by means of ^{1}H NMR analysis, with 1,1,2,2-tetrachloroethane as an internal standard. [d]Yield in parenthesis is the isolated yield.

***^{19}F NMR monitoring*:**

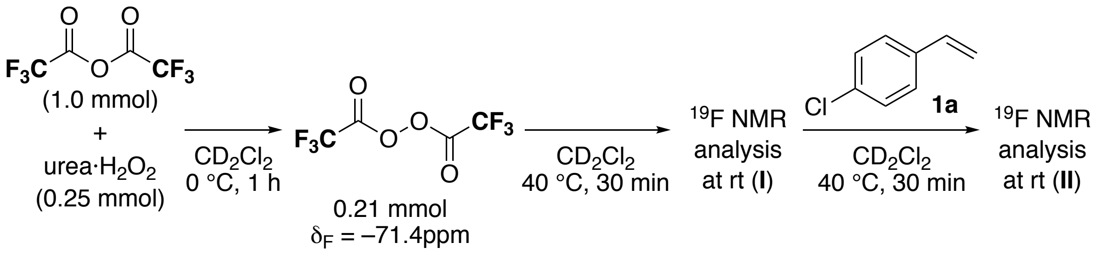

Trifluoroacetic anhydride (0.14 mL, 1.0 mmol) was slowly added to a suspension of urea · H_2O_2 (24 mg, 0.25 mmol) in CD_2Cl_2 (0.6 mL) in a Schlenk tube at 0 °C, and the mixture

[2]L. Zhou, J. Chen, C. K. Tan, Y.-Y. Yeung, *J. Am. Chem. Soc.*, 2011, **133**, 9164.
[3]Y. Arai, R. Tomita, G. Ando, T. Koike, M. Akita, *Chem. Eur. J.*, 2016, **22**, 1262.

was stirred for 1 h. The obtained colorless solution containing bis(trifluoroacetyl)peroxide (BTFAP) was transferred to a valve NMR tube containing α,α,α-trifluorotoluene (13 mg, 0.09 mmol) as an internal standard under a N_2 atmosphere. The ^{19}F NMR spectrum of the sample was measured at room temperature, and 0.21 mmol of the peroxide was found to have been formed. After the measurement, the NMR sample was warmed to 40 °C on an oil bath. After 30 min, ^{19}F NMR measurement was conducted at room temperature (**I**) (Figure S1); and no change of the spectral signals or integration values was observed. Then, styrene **1a** (14 mg, 0.10 mmol) was added to the sample solution at room temperature, and the mixture was warmed to 40 °C on an oil bath. After 30 min, the ^{19}F NMR spectrum of the sample was measured at room temperature (**II**); the results indicated the presence of 0.09 mmol (86% yield based on **1a**) of oxy-trifluoromethylation product **2a** and 0.10 mmol of BTFAP. This shows that styrene is essential for decomposition of BTFAP and CF_3 radical generation.

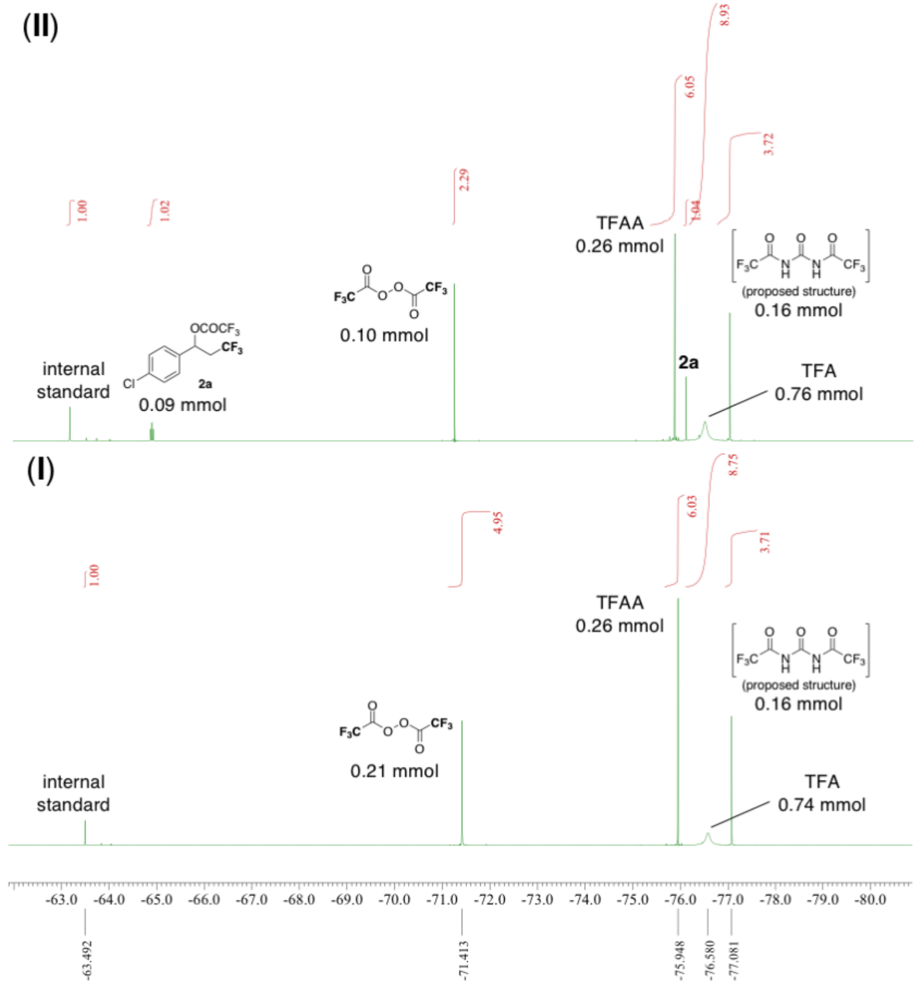

Figure S1. **^{19}F NMR monitoring of the decomposition of BTFAP**

Reaction of N-(2-vinylphenethyl)-p-toluenesulfonamide:

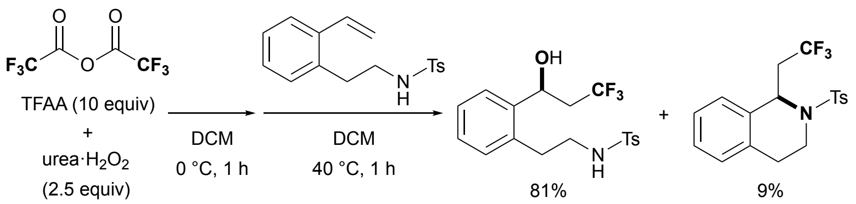

To a suspension of urea·H_2O_2 (47 mg, 0.50 mmol) in DCM (1 mL) trifluoroacetic anhydride (0.28 mL, 2.0 mmol) was slowly added at 0 °C. After stirring for 1 h, *N*-(2-vinylphenethyl)-*p*-toluenesulfonamide (60 mg, 0.20 mmol) was added. Then, the mixture was immediately warmed to 40 °C, further stirred for 3 h, then diluted with Et_2O (5 mL), quenched with saturated K_2CO_3 solution at 0 °C, again stirred for 20 min. The phases were separated and the aqueous layer was extracted with Et_2O (2 x 5 mL). The combined organic phase was dried over Na_2SO_4, filtered and concentrated *in vacuo*. Purification of the crude product by column chromatography on silica gel afforded *N*-(2-(3,3,3-trifluoro-1-hydroxypropyl)phenethyl)-*p*-toluenesulfonamide (63 mg, 81% yield) and 2-tosyl-1-(2,2,2-trifluoroethyl)-1,2,3,4-tetrahydroisoquinoline (7 mg, 9%).

<N-(2-(3,3,3-trifluoro-1-hydroxypropyl)phenethyl)-p-toluenesulfonamide>

^{1}H NMR (400 MHz, $CDCl_3$)

2.28–2.43 (m, 2H), 2.40 (s, 3H), 2.56–2.72 (m, 1H), 2.80 (ddd, *J* = 14.0, 6.9, 6.7 Hz, 1H), 2.89 (ddd, *J* = 14.0, 7.0, 6.9 Hz, 1H), 3.14 (ddd, *J* = 13.0, 7.0, 6.9 Hz), 3.22 (ddd, *J* = 13.0, 6.9, 6.7 Hz, 1H), 4.85–5.10 (br, 1H), 5.24 (dd, *J* = 8.9, 3.3 Hz, 1H), 7.07 (d, *J* = 7.5 Hz, 1H), 7.18–7.29 (overlap, 4H), 7.41 (d, *J* = 7.5 Hz, 1H), 7.63 (d, *J* = 7.9 Hz, 2H)

^{13}C NMR (100 MHz, $CDCl_3$)

21.6, 32.2, 42.2 (q, *J* = 27 Hz). 44.2, 64.9 (q, *J* = 2.9 Hz), 126.0 (q, *J* = 277 Hz), 126.4, 127.1 (2C), 127.7, 128.7, 129.9 (2C), 130.3, 134.9, 136.8, 140.5, 143.7.

^{19}F NMR (376 MHz, $CDCl_3$)

–63.7 (t, *J* = 10.1 Hz)

IR (neat, cm^{-1})

3287, 1325, 1260, 1156, 1093, 815, 761, 662.

HRMS-ESI (*m*/*z*)

$[M+Na]^+$ calcd. for $C_{18}H_{20}F_3NO_3SNa$, 410.1008; found, 410.1009.

<2-Tosyl-1-(2,2,2-trifluoroethyl)-1,2,3,4-tetrahydroisoquinoline>

^{1}H NMR (400 MHz, $CDCl_3$)

2.35 (s, 3H), 2.41–2.56 (m, 1H), 2.56–2.82 (m, 3H), 3.48 (ddd, *J* = 14.2, 10.3,

5.3 Hz, 1H), 3.77 (dddd, *J* = 14.2, 6.4, 3.7, 0.9 Hz, 1H), 5.42 (dd, *J* = 8.4, 5.1 Hz, 1H), 6.96 (d, *J* = 7.0 Hz, 1H), 7.08 (dd, *J* = 7.0, 1.8 Hz, 1H), 7.12–7.18 (m, 4H), 7.63 (d, *J* = 8.3 Hz, 2H)

^{13}C NMR (100 MHz, $CDCl_3$)

21.6, 26.3, 39.3, 41.3 (q, *J* = 27 Hz), 51.1 (q, *J* = 2.9 Hz), 125.3 (q, *J* = 278 Hz), 126.7, 127.0, 127.4 (2C), 127.8, 129.3, 129.6 (2C), 133.3, 134.4, 137.0, 143.6.

^{19}F NMR (376 MHz, $CDCl_3$)

–63.3 (t, *J* = 11.6 Hz)

IR (neat, cm^{-1})

1339, 1266, 1165, 1091, 941, 815, 734, 659.

HRMS-ESI (*m*/*z*)

$[M+H]^+$ calcd. for $C_{18}H_{19}F_3NO_2S$, 370.1083; found, 370.1090.

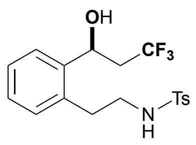

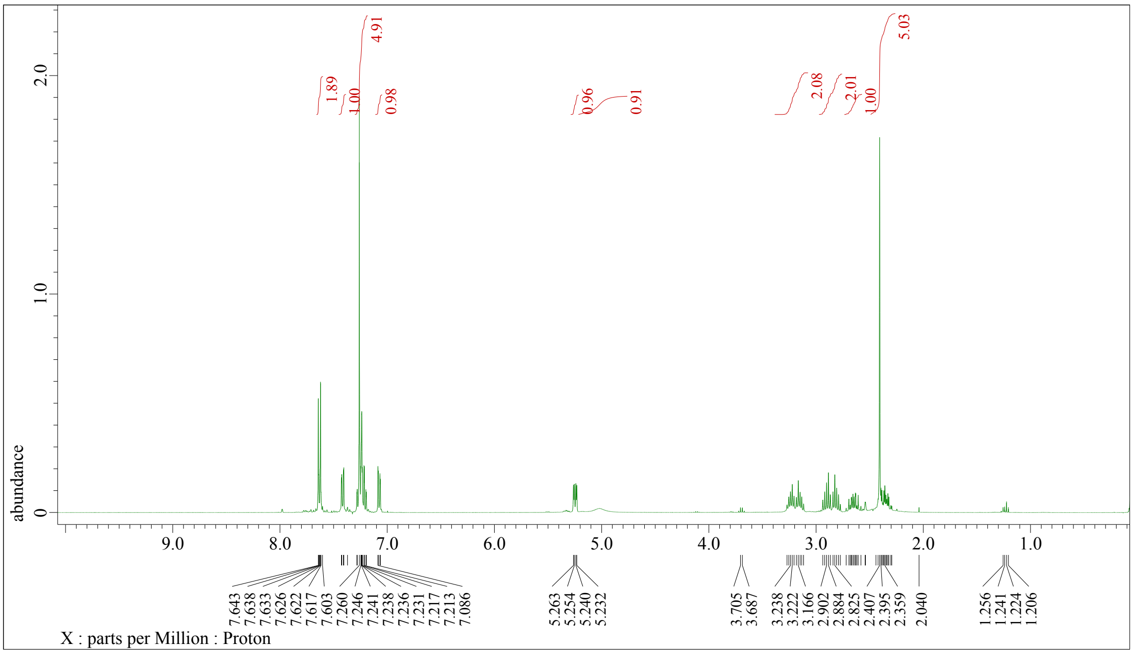

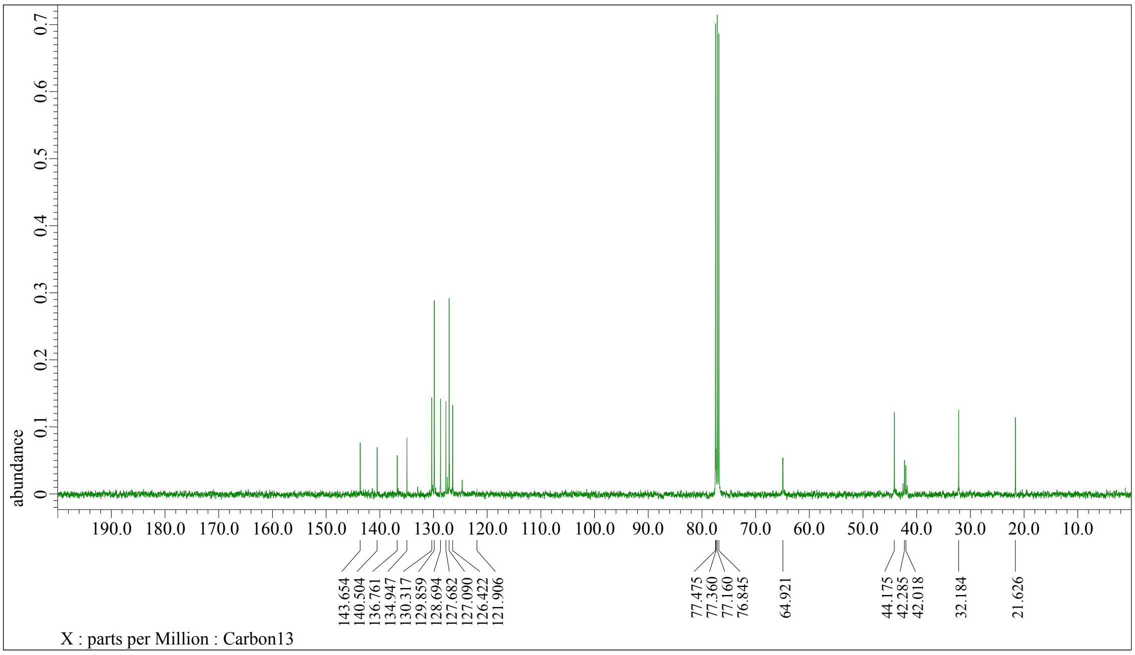

Figure S2. NMR spectra of *N*-(2-(3,3,3-trifluoro-1-hydroxypropyl)phenethyl)-*p*-toluenesulfonamide

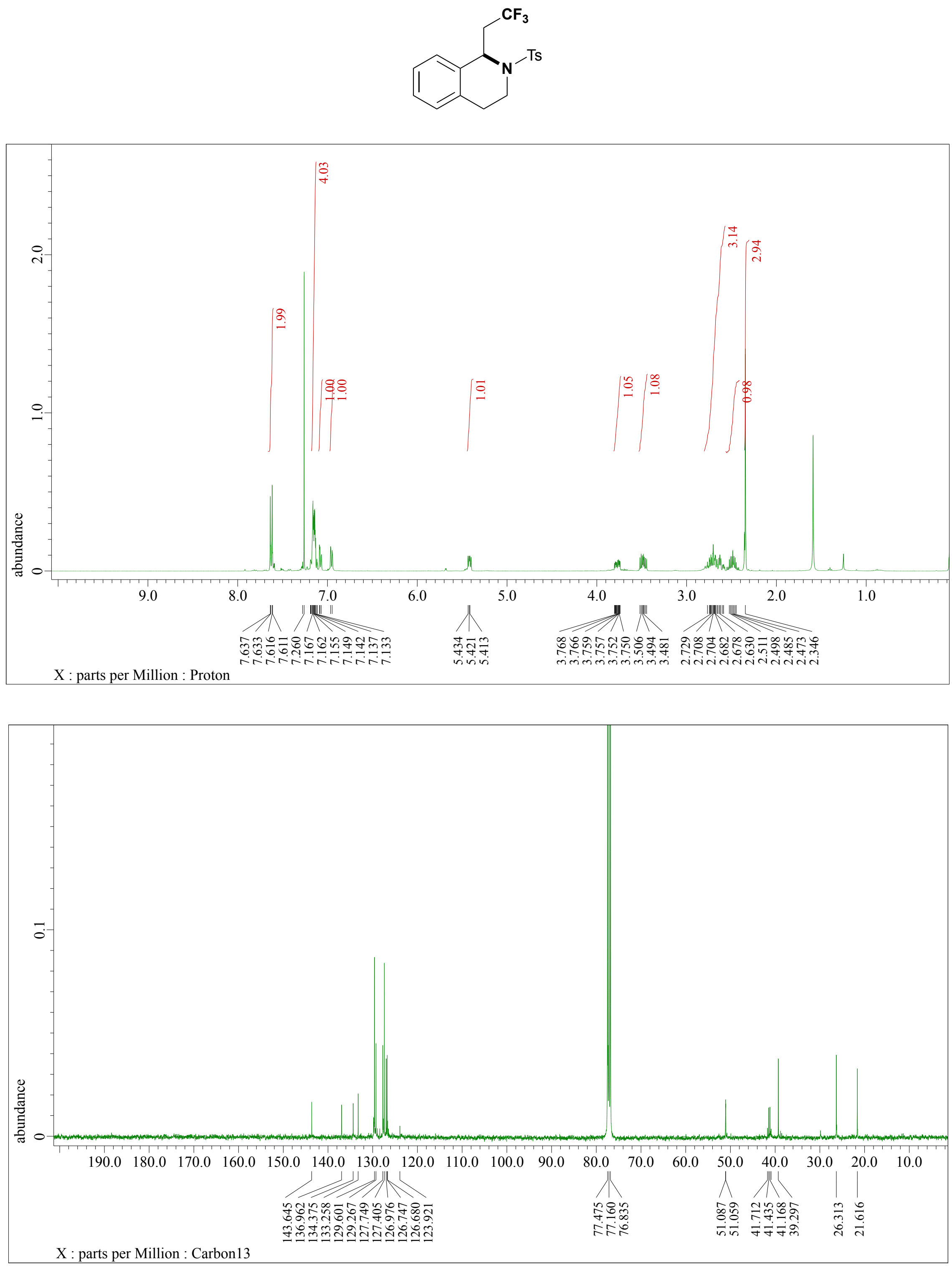

Figure S3. NMR spectra of *2-Tosyl-(2,2,2-trifluoroethyl)-1,2,3,4-tetrahydro-isoquinoline*

3. Experimental procedures

Preparation of aminoalkene 3b

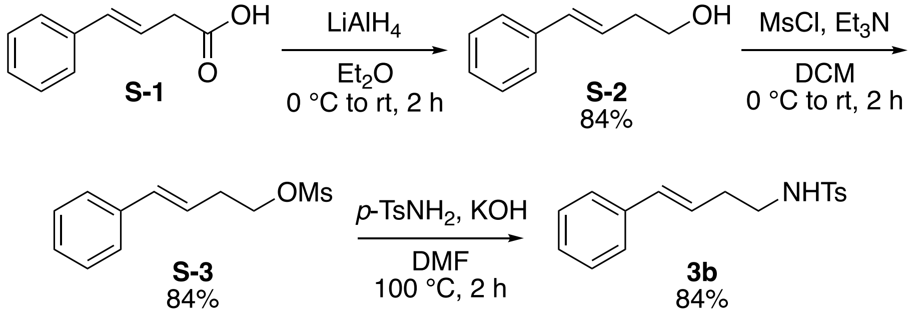

Substrate **3b** was synthesized according to the literature procedure for preparing **3a**.[2] A solution of carboxylic acid **S-1** (2.0 g, 12 mmol) in dry Et_2O (15 mL) was added dropwise to a suspension of lithium aluminum hydride (0.9 g, 24 mmol) in dry Et_2O (35 mL) at 0 °C. The mixture was stirred at room temperature for 2 h, and then the reaction was quenched by careful and sequential addition of H_2O (7 mL) and 2 M NaOH solution (7 mL) at 0 °C. The resulting suspension was filtered through a Celite pad and the filtrate was dried over Na_2SO_4, filtered and concentrated *in vacuo* to provide **S-2** (1.5 g, 84% yield) as a yellow oil. Methanesulfonyl chloride (0.97 mL, 13 mmol) was added dropwise to a solution of **S-2** (1.5 g, 10 mmol) and triethylamine (1.9 mL, 14 mmol) in dry DCM (30 mL) at 0 °C. The solution was stirred at 0 °C for 10 min, then warmed to room temperature, and stirred further for 1 h. The reaction mixture was diluted with DCM (10 mL) and washed with 1 M HCl aqueous solution (40 mL), saturated $NaHCO_3$ solution (40 mL) and brine (40 mL). The organic phase was dried over Na_2SO_4, filtered and concentrated *in vacuo*. Purification by means of column chromatography (SiO_2; EtOAc/hexane = 20/80) provided **S-3** (2.2 g, 93% yield) as a yellow oil. A solution of *p*-toluenesulfonamide (4.4 g, 26 mmol) and potassium hydroxide (1.4 g, 26 mmol) in dry DMF (70 mL) was heated to 100 °C for 0.5 h. Then a solution of **S-3** (2.1 g, 13 mmol) in dry DMF (40 mL) was added dropwise. The mixture was stirred at 100 °C for 2 h, cooled to room temperature, quenched with water (80 mL), and extracted with Et_2O (3 x 60 mL). The combined organic phase was washed with water (60 mL) and brine (60 mL), dried over Na_2SO_4, filtered and concentrated *in vacuo*. Purification of the residue by means of column chromatography (SiO_2; EtOAc/hexane = 20/80) provided **3b** (2.0 g, 64% yield) as a colorless oil, whose spectroscopic data matched reported values.[4]

[4]J. Ciesielski, G. Dequirez, P. Retailleau, V. Gandon, P. Dauban, *Chem. Eur, J.* 2016, **22**, 9338.

Preparation of aminoalkene 3c

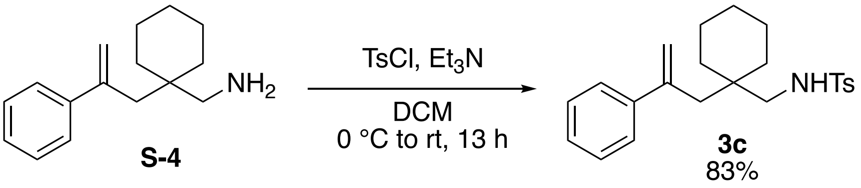

p-Toluenesulfonyl chloride (0.46 mg, 2.4 mmol) was added to a solution of **S-4**[5] (0.50 g, 2.2 mmol) and triethylamine (0.6 mL, 4.4 mmol) in dry DCM (9 mL) at 0 °C. The reaction mixture was stirred at room temperature overnight, then diluted with DCM (10 mL) and washed with 1 M HCl aqueous solution (2 x 15 mL). The organic phase was dried over Na_2SO_4, filtered and concentrated *in vacuo* to give a yellow oil. Purification by column chromatography (SiO_2; EtOAc/hexane = 10/90) provided **3c** (0.70 g, 83% yield) as a white solid.

^{1}H NMR (400 MHz, $CDCl_3$)

1.21–1.45 (overlap, 10H), 2.42 (s, 3H), 2.49 (s, 2H), 2.50 (d, *J* = 7.6 Hz, 2H), 3.93 (t, *J* = 7.6 Hz, 1H), 5.03 (d, *J* = 1.6 Hz, 1H), 5.17 (d, *J* = 1.6 Hz, 1H), 7.22 (d, *J* = 8.4 Hz, 2H), 7.27–7.33 (overlap, 5H), 7.39 (d, *J* = 8.4 Hz, 2H).

^{13}C NMR (100 MHz, $CDCl_3$)

21.5 (2C), 21.6, 26.1, 33.9 (2C), 37.6, 42.5, 48.6, 118.0, 126.5 (2C), 127.0 (2C), 127.7, 128.9 (2C), 129.6 (2C), 136.9, 143.1, 143.8, 146.0.

IR (neat, cm^{-1})

3284, 1454, 1415, 1325, 1161, 1093, 1071, 906, 813, 779, 705, 662.

HRMS-ESI (*m*/*z*)

$[M+Na]^+$ calcd. for $C_{23}H_{29}NO_2SNa$, 406.1817; found, 406.1818.

Oxy- and amino-perfluoroalkylation of alkenes: general procedure

To a suspension of urea·H_2O_2 (47 mg, 0.50 mmol) in DCM (1 mL) perfluoro acid anhydride (2.0 mmol) was slowly added at 0 °C. After stirring for 1 h, styrene (0.20 mmol) was added. Then, the mixture was immediately warmed to 40 °C, further stirred for 1 h, then diluted with Et_2O (5 mL), quenched with saturated K_2CO_3 solution at 0 °C, again stirred for 20 min. The phases were separated and the aqueous layer was extracted with Et_2O (2 x 5 mL).[6] The combined organic phase was dried over Na_2SO_4, filtered and

[5] J.-S. Lin, P. Yu, L. Huang, P. Zhang, B. Tan, X.-Y. Liu, *Angew. Chem. Int. Ed.*, 2015, **54**, 7847.
[6] The combined organic phase was checked with XploSens PS® to confirm the absence of peroxide, and the water phase was treated with saturated $Na_2S_2O_3$ to decompose H_2O_2.

concentrated *in vacuo*. Purification of the crude product by column chromatography on silica gel afforded the target compound.

Synthesis of 1-(4-chlorophenyl)-3,3,3-trifluoropropyl 2,2,2-trifluoroacetate (2a):

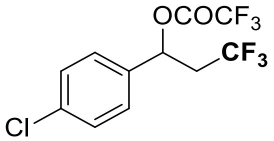

The reaction was carried out according to the general procedure. The target compound **2a** was obtained as a colorless oil (51 mg, 80% yield) after purification by column chromatography (SiO_2; 100% hexane).

^{1}H NMR (400 MHz, $CDCl_3$)

2.62 (dqd, *J* = 15.7, 10.1, 2.8 Hz, 1H), 2.95 (m, 1H), 6.18 (dd, *J* = 7.6, 2.8 Hz, 1H), 7.33 (d, *J* = 6.8 Hz, 2H), 7.41 (d, *J* = 6.8 Hz, 2H).

^{13}C NMR (100 MHz, $CDCl_3$)

40.1 (q, *J* = 29 Hz), 73.0 (q, *J* = 2.9 Hz), 114.4 (q, *J* = 285 Hz), 124.7 (q, *J* = 277 Hz), 128.0 (2C), 129.7 (2C), 134.5, 136.1, 156.2 (q, *J* = 43 Hz).

^{19}F NMR (376 MHz, $CDCl_3$)

–64.3 (t, *J* = 10.1 Hz, 3F), –75.2 (s, 3F).

IR (neat, cm^{-1})

1793, 1495, 1392, 1378, 1338, 1323, 1283, 1253, 1226, 1143, 1130, 1096, 1064, 1016, 828, 819, 668.

HRMS-EI (*m/z*)

[M] calcd. for $C_{11}H_7ClF_6O_2$, 320.0039; found, 320.0027.

Synthesis of 1-(4-fluorophenyl)-3,3,3-trifluoropropyl 2,2,2-trifluoroacetate (2b):

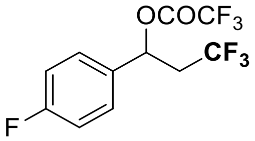

The reaction was carried out according to the general procedure. The target compound **2b** was obtained as a yellow oil (54 mg, 88% yield) after purification by column chromatography (SiO_2; 100% hexane).

Procedure for gram-scale synthesis: To a suspension of urea·H_2O_2 (3.9 g, 41 mmol) in DCM (82 mL), trifluoroacetic anhydride (23.1 mL, 164 mmol) was slowly added at 0 °C. After stirring for 1 h, 4-fluorostyrene (2.0 g, 16 mmol) was added. The mixture was immediately warmed to 40 °C and stirred for further 1 h. After dilution with DCM (50 mL), the reaction was quenched with saturated K_2CO_3 solution at 0 °C for 20 min. The phases were separated and the aqueous layer was extracted with DCM (2 x 50 mL).[6] The

combined organic phase was dried over Na_2SO_4, filtered and concentrated *in vacuo* to give pure **2b** (4.7 g, 93%) as a yellow oil.

^{1}H NMR (400 MHz, $CDCl_3$)

2.62 (dqd, J = 15.5, 10.1, 3.6 Hz, 1H), 2.96 (m, 1H), 6.20 (dd, J = 9.6, 3.6 Hz, 1H), 7.12 (m, 2H), 7.39 (m, 2H).

^{13}C NMR (100 MHz, $CDCl_3$)

40.2 (q, J = 29 Hz), 73.1 (q, J = 2.8 Hz), 114.4 (q, J = 285 Hz), 116.5 (d, J = 22 Hz, 2C), 124.7 (q, J = 277 Hz), 128.7 (d, J = 8.6 Hz, 2C), 132.0 (d, J = 3.9 Hz), 156.2 (q, J = 43 Hz), 163.5 (d, J = 249 Hz).

^{19}F NMR (376 MHz, $CDCl_3$)

–64.3 (t, J = 10.1 Hz, 3F), –75.2 (s, 3F), –110.6 (m, 1F).

IR (neat, cm^{-1})

1791, 1515, 1340, 1229, 1129, 1098, 1064, 831, 771, 735.

HRMS-EI (*m*/*z*)

[M] calcd. for $C_{11}H_7F_7O_2$, 304.0334; found, 304.0312.

Synthesis of 1-(4-bromophenyl)-3,3,3-trifluoropropyl 2,2,2-trifluoroacetate (2c):

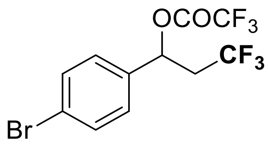

The reaction was carried out according to the general procedure. The target compound **2c** was obtained as a colorless oil (56 mg, 77% yield) after purification by column chromatography (SiO_2; 100% hexane).

^{1}H NMR (400 MHz, $CDCl_3$)

2.62 (dqd, J = 15.6, 10.1, 3.6 Hz, 1H), 2.95 (m, 1H), 6.17 (dd, J = 9.6, 3.6 Hz, 1H), 7.27 (d, J = 8.4 Hz, 2H), 7.57 (d, J = 8.4 Hz, 2H).

^{13}C NMR (100 MHz, $CDCl_3$)

40.1 (q, J = 29 Hz), 73.1 (q, J = 3.8 Hz), 114.4 (q, J = 285 Hz), 124.2, 124.7 (q, J = 277 Hz), 128.2 (2C), 132.7 (2C), 135.1, 156.2 (q, J = 43 Hz).

^{19}F NMR (376 MHz, $CDCl_3$)

–64.3 (t, J = 10.1 Hz, 3F), –75.2 (s, 3F).

IR (neat, cm^{-1})

1791, 1491, 1377, 1338, 1253, 1225, 1129, 1102, 1075, 1013, 816, 735, 668.

HRMS-EI (*m*/*z*)

[M] calcd. for $C_{11}H_7BrF_6O_2$, 363.9534; found, 363.9506.

Synthesis of 1-(4-acetoxyphenyl)-3,3,3-trifluoropropyl 2,2,2-trifluoroacetate (2d):

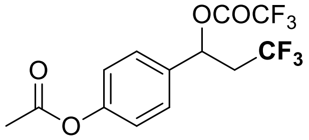

The reaction was carried out according to the general procedure. The target compound **2d** was obtained as a colorless oil (69 mg, quantitative yield) after purification by column chromatography (SiO_2; EtOAc/hexane = 5/95).

^{1}H NMR (400 MHz, $CDCl_3$)

2.31 (s, 3H), 2.62 (dqd, *J* = 15.6, 10.1, 3.2 Hz, 1H), 2.96 (m, 1H), 6.23 (dd, *J* = 10.0, 3.2 Hz, 1H), 7.16 (d, *J* = 8.4 Hz, 2H), 7.41 (d, *J* = 8.4 Hz, 2H).

^{13}C NMR (100 MHz, $CDCl_3$)

21.2, 40.2 (q, *J* = 29 Hz), 73.0 (q, *J* = 3.0 Hz), 114.4 (q, *J* = 285 Hz), 122.7 (2C), 124.8 (q, *J* = 277 Hz), 127.8 (2C), 133.6, 151.7, 156.2 (q, *J* = 43 Hz), 169.3.

^{19}F NMR (376 MHz, $CDCl_3$)

–64.5 (t, *J* = 10.1 Hz, 3F), –75.2 (s, 3F).

IR (neat, cm^{-1})

1791, 1770, 1374, 1341, 1258, 1216, 1199, 1130, 1104, 1064, 1018, 913, 800, 774, 631.

HRMS-EI (*m*/*z*)

[M] calcd. for $C_{13}H_{10}F_6O_4$, 344.0483; found, 344.0447.

Synthesis of 3,3,3-trifluoro-1-phenylpropyl 2,2,2-trifluoroacetate (2e):

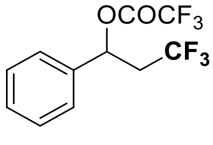

The reaction was carried out according to the general procedure. The target compound **2e** was obtained as a colorless oil (90% yield based on ^{1}H and ^{19}F NMR).[7] The crude product was purified by column chromatography (SiO_2; 100% hexane) to obtain an analytical sample (11 mg).

^{1}H NMR (400 MHz, $CDCl_3$)

2.63 (dqd, *J* = 15.5, 10.1, 3.2 Hz, 1H), 2.98 (m, 1H), 6.23 (dd, *J* = 9.6, 3.2 Hz, 1H), 7.37–7.45 (overlap, 5H).

^{13}C NMR (100 MHz, $CDCl_3$)

40.3 (q, *J* = 29 Hz), 73.7 (q, *J* = 2.9 Hz), 114.5 (q, *J* = 284 Hz), 124.8 (q, *J* =

[7]Compound **2e** appeared to be volatile, hampering full isolation.

276 Hz), 126.5 (2C), 129.4 (2C), 129.9, 136.2, 156.3 (q, *J* = 44 Hz).

^{19}F NMR (376 MHz, $CDCl_3$)

–64.4 (d, *J* = 10.1 Hz, 3F), –75.2 (s, 3F).

IR (neat, cm^{-1})

1791, 1347, 1332, 1254, 1226, 1129, 1077, 1062, 773, 763, 734, 698, 668, 609.

HRMS-EI (*m*/*z*)

[M] calcd. for $C_{11}H_8F_6O_2$, 286.0428; found, 286.0417.

Synthesis of 1-(4-methylphenyl)-3,3,3-trifluoropropyl 2,2,2-trifluoroacetate (2f):

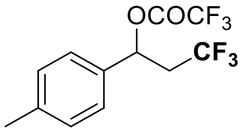

The reaction was carried out according to the general procedure. The target compound **2f** was obtained as a colorless oil (43 mg, 72% yield) after purification by column chromatography (SiO_2; 100% hexane).

^{1}H NMR (400 MHz, $CDCl_3$)

2.37 (s, 3H), 2.61 (dqd, *J* = 15.6, 10.1, 3.6 Hz, 1H), 2.96 (m, 1H), 6.19 (dd, *J* = 9.6, 3.6 Hz, 1H), 7.22 (m, 2H), 7.27 (m, 2H).

^{13}C NMR (100 MHz, $CDCl_3$)

21.4, 40.2 (q, *J* = 29 Hz), 73.7 (q, *J* = 3.8 Hz), 114.5 (q, *J* = 285 Hz), 124.9 (q, *J* = 277 Hz), 126.5 (2C), 130.0 (2C), 133.2, 140.1, 156.3 (q, *J* = 43 Hz).

^{19}F NMR (376 MHz, $CDCl_3$)

–64.4 (t, *J* = 10.1 Hz, 3F), –75.2 (s, 3F).

IR (neat, cm^{-1})

1791, 1378, 1324, 1254, 1227, 1130, 1058, 813, 733, 668, 652.

HRMS-EI (*m*/*z*)

[M] calcd. for $C_{12}H_{10}F_6O_2$, 300.0585; found, 300.0566.

Synthesis of 1-(2-(chloromethyl)phenyl)-3,3,3-trifluoropropyl 2,2,2-trifluoroacetate (2g):

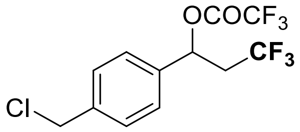

The reaction was carried out according to the general procedure. The target compound **2g** was obtained as a colorless oil (50 mg, 75% yield) after purification by column chromatography (SiO_2; 100% hexane).

^{1}H NMR (400 MHz, $CDCl_3$)

2.62 (dqd, *J* = 15.6, 10.1, 3.5 Hz, 1H), 2.96 (m, 1H), 4.59 (s, 2H), 6.22 (dd, *J* = 9.7, 3.5 Hz, 1H), 7.38 (d, *J* = 8.4 Hz, 2H), 7.46 (d, *J* = 8.4 Hz, 2H).

^{13}C NMR (100 MHz, $CDCl_3$)

40.2 (q, *J* = 29 Hz), 45.5, 73.3 (q, *J* = 2.9 Hz), 114.4 (q, *J* = 284 Hz), 124.8 (q, *J* = 277 Hz), 126.9 (2C), 129.6 (2C), 136.3, 139.4, 156.2 (q, *J* = 43 Hz).

^{19}F NMR (376 MHz, $CDCl_3$)

–64.3 (t, *J* = 10.1 Hz, 3F), –75.1 (s, 3F).

IR (neat, cm^{-1})

1791, 1340, 1314, 1275, 1254, 1227, 1130, 1063, 832, 789, 734, 684, 668, 658.

HRMS-EI (*m*/*z*)

[M] calcd. for $C_{12}H_9ClF_6O_2$, 334.0195; found, 334.0179.

Synthesis of 1-(4-(*tert*-butyl)phenyl)-3,3,3-trifluoropropyl 2,2,2-trifluoroacetate (2h):

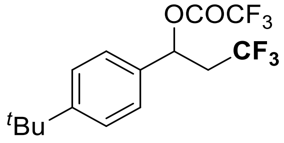

The reaction was carried out according to the general procedure. The target compound **2h** was obtained as a colorless oil (60 mg, 87% yield) after purification by column chromatography (SiO_2; 100% hexane).

^{1}H NMR (400 MHz, $CDCl_3$)

1.32 (s, 9H), 2.61 (dqd, *J* = 15.6, 10.1, 3.2 Hz, 1H), 2.97 (m, 1H), 6.23 (dd, *J* = 9.6, 3.2 Hz, 1H), 7.31 (d, *J* = 8.4 Hz, 2H), 7.43 (d, *J* = 8.4 Hz, 2H).

^{13}C NMR (100 MHz, $CDCl_3$)

31.3 (3C), 34.9, 40.2 (q, *J* = 29 Hz), 73.6 (q, *J* = 2.9 Hz), 114.5 (q, *J* = 285 Hz), 124.9 (q, *J* = 277 Hz), 126.2 (2C), 126.3 (2C), 133.2, 153.2, 156.3 (q, *J* = 43 Hz).

^{19}F NMR (376 MHz, $CDCl_3$)

–64.5 (t, *J* = 10.1 Hz, 3F), –75.2 (s, 3F).

IR (neat, cm^{-1})

1791, 1394, 1367, 1340, 1253, 1224, 1132, 1063, 830, 820, 774, 733, 639.

HRMS-EI (*m*/*z*)

[M] calcd. for $C_{15}H_{16}F_6O_2$, 342.1054; found, 342.1029.

Synthesis of 1-(4-methoxyphenyl)-3,3,3-trifluoropropyl 2,2,2-trifluoroacetate (2i):

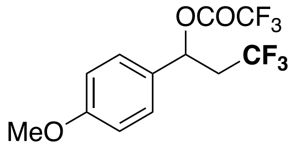

To a suspension of urea·H_2O_2 (47 mg, 0.50 mmol) in DCM (1 mL), trifluoroacetic anhydride (0.28 mL, 2.0 mmol) was slowly added at 0 °C. After stirring for 1 h, Cs_2CO_3 (326 mg, 1.0 mmol) was added, followed by styrene **1i** (27 mg, 0.20 mmol). The mixture was stirred for further 10 min. After addition of Et_2O (5 mL), the reaction was quenched with saturated K_2CO_3 solution at 0 °C for 20 min. The phases were separated and the aqueous layer was extracted with Et_2O (2 x 5 mL). The combined organic phase was dried over Na_2SO_4, filtered and concentrated *in vacuo*. Purification of the crude product by column chromatography on silica gel (SiO_2; EtOAc/hexane = 10/90) afforded the target product **2i** as a colorless oil (24 mg, 38% yield).

^{1}H NMR (400 MHz, $CDCl_3$)

2.61 (dqd, *J* = 15.5, 10.1, 3.6 Hz, 1H), 2.96 (m, 1H), 3.82 (s, 3H), 6.18 (dd, *J* = 9.5, 3.6 Hz, 1H), 6.93 (d, *J* = 8.8 Hz, 2H), 7.32 (d, *J* = 8.8 Hz, 2H).

^{13}C NMR (100 MHz, $CDCl_3$)

40.1 (q, *J* = 29 Hz), 55.5, 73.6 (q, *J* = 2.9 Hz), 114.5 (q, *J* = 286 Hz), 114.7 (2C), 124.9 (q, *J* = 277 Hz), 128.1, 128.3 (2C), 156.3 (q, *J* = 43 Hz), 160.8.

^{19}F NMR (376 MHz, $CDCl_3$)

–64.4 (t, *J* = 10.1 Hz, 3F), –75.2 (s, 3F).

IR (neat, cm^{-1})

2924, 2853, 1791, 1617, 1506, 1308, 1279, 1226, 1131, 1063, 1033, 829.

HRMS-EI (*m*/*z*)

[M] calcd. for $C_{12}H_{10}F_6O_3$, 316.0534; found, 316.0544.

Synthesis of 1-(3-(trifluoromethyl)phenyl)-3,3,3-trifluoropropyl 2,2,2-trifluoroacetate (2j):

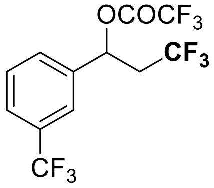

The reaction was carried out in DCE at 60 °C for the second step, but otherwise according to the general procedure. The target compound **2j** was obtained as a colorless oil (52 mg, 74% yield) after purification by column chromatography (SiO_2; 100% hexane).

^{1}H NMR (400 MHz, $CDCl_3$)

2.66 (dqd, *J* = 15.5, 10.1, 3.6 Hz, 1H), 2.99 (m, 1H), 6.27 (dd, *J* = 9.6, 3.6 Hz, 1H), 7.59 (m, 2H), 7.64 (br, 1H), 7.69 (m, 1H).

^{13}C NMR (100 MHz, $CDCl_3$)

40.2 (q, *J* = 29 Hz), 72.9 (q, *J* = 2.9 Hz), 114.4 (q, *J* = 285 Hz), 123.6 (q, *J* = 272 Hz), 123.3 (q, *J* = 3.8 Hz), 124.6 (q, *J* = 277 Hz), 126.9 (q, *J* = 3.7 Hz), 129.9, 130.2, 132.0 (q, *J* = 32 Hz), 137.1, 156.2 (q, *J* = 43 Hz).

^{19}F NMR (376 MHz, $CDCl_3$)

–62.8 (s, 3F), –64.2 (t, *J* = 10.1 Hz, 3F), –75.1 (s, 3F).

IR (neat, cm^{-1})

1793, 1330, 1252, 1229, 1203, 1128, 1094, 1076, 805, 777, 736, 702, 668.

HRMS-EI (*m*/*z*)

[M] calcd. for $C_{12}H_7F_9O_2$, 354.0302; found, 354.0283.

Synthesis of 1-(3-fluorophenyl)-3,3,3-trifluoropropyl 2,2,2-trifluoroacetate (2k):

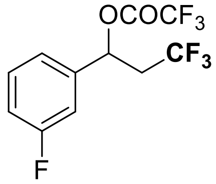

The reaction was carried out in DCE at 60 °C for the second step, but otherwise according to the general procedure. The target compound **2k** was obtained as a colorless oil (47 mg, 78% yield) after purification by column chromatography (SiO_2; 100% hexane).

^{1}H NMR (400 MHz, $CDCl_3$)

2.63 (dqd, *J* = 15.6, 10.1, 3.2 Hz, 1H), 2.95 (m, 1H), 6.20 (dd, *J* = 9.6, 3.2 Hz, 1H), 7.11 (m, 2H), 7.17 (m, 1H), 7.41 (m, 1H).

^{13}C NMR (100 MHz, $CDCl_3$)

40.2 (q, *J* = 29 Hz), 72.9, 113.6 (d, *J* = 22 Hz), 114.4 (q, *J* = 285 Hz), 117.1 (d, *J* = 21 Hz), 122.2 (d, *J* = 2.8 Hz), 124.7 (q, *J* = 277 Hz), 131.2 (d, *J* = 8.7 Hz), 138.4 (d, *J* = 7.7 Hz), 156.2 (q, *J* = 43 Hz), 163.1 (d, *J* = 248 Hz).

^{19}F NMR (376 MHz, $CDCl_3$)

–64.3 (t, *J* = 10.1 Hz, 3F), –75.1 (s, 3F), –110.6 (m, 1F).

IR (neat, cm^{-1})

1793, 1596, 1491, 1378, 1341, 1258, 1227, 1144, 1127, 1064, 876, 789, 779, 735, 696, 668.

HRMS-EI (*m*/*z*)

[M] calcd. for $C_{11}H_7F_7O_2$, 304.0334; found, 304.0314.

Synthesis of 1-(2-chlorophenyl)-3,3,3-trifluoropropyl 2,2,2-trifluoroacetate (2l):

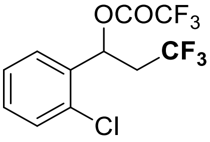

The reaction was carried out according to the general procedure. The target compound **2l** was obtained as a colorless oil (43 mg, 67% yield) after purification by column chromatography (SiO_2; 100% hexane).

^{1}H NMR (400 MHz, $CDCl_3$)

2.64–2.90 (overlap, 2H), 6.64 (dd, *J* = 9.6, 2.8 Hz, 1H), 7.33–7.38 (overlap, 2H), 7.40–7.46 (overlap, 2H).

^{13}C NMR (100 MHz, $CDCl_3$)

39.2 (q, *J* = 29 Hz), 70.4 (q, *J* = 3.9 Hz), 114.5 (q, *J* = 285 Hz), 124.8 (q, *J* = 277 Hz), 126.6, 128.0, 130.4, 130.7, 132.0, 134.2, 156.0 (q, *J* = 43 Hz).

^{19}F NMR (376 MHz, $CDCl_3$)

–64.5 (t, *J* = 10.1 Hz, 3F), –75.1 (s, 3F).

IR (neat, cm^{-1})

1793, 1379, 1344, 1252, 1225, 1134, 1054, 1037, 757, 736, 711, 668, 612.

HRMS-EI (*m*/*z*)

[M] calcd. for $C_{11}H_7ClF_6O_2$, 320.0039; found, 320.0017.

Synthesis of 1-(2-bromophenyl)-3,3,3-trifluoropropyl 2,2,2-trifluoroacetate (2m):

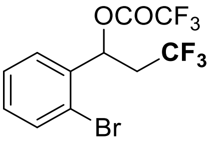

The reaction was carried out according to the general procedure. The target compound **2m** was obtained as a colorless oil (54 mg, 74% yield) after purification by column chromatography (SiO_2; 100% hexane).

^{1}H NMR (400 MHz, $CDCl_3$)

2.63–2.88 (overlap, 2H), 6.60 (dd, *J* = 9.6, 2.8 Hz, 1H), 7.22–7.29 (m, 1H), 7.39–7.42 (overlap, 2H), 7.62 (d, *J* = 7.6 Hz, 1H).

^{13}C NMR (100 MHz, $CDCl_3$)

39.3 (q, *J* = 29 Hz), 72.6 (q, *J* = 2.8 Hz), 114.5 (q, *J* = 285 Hz), 121.6, 124.7 (q, *J* = 277 Hz), 126.7, 128.6, 131.0, 133.7, 135.9, 155.9 (q, *J* = 43 Hz).

^{19}F NMR (376 MHz, $CDCl_3$)

–64.5 (t, *J* = 10.1 Hz, 3F), –75.1 (s, 3F).

IR (neat, cm^{-1})

1793, 1474, 1438, 1392, 1343, 1314, 1284, 1251, 1225, 1205, 1131, 1065, 1026, 843, 756, 736, 722, 691, 668, 611.

HRMS-EI (*m*/*z*)

[M] calcd. for $C_{11}H_7BrF_6O_2$, 363.9534; found, 363.9505.

Synthesis of 1-(2-methylphenyl)-3,3,3-trifluoropropyl 2,2,2-trifluoroacetate (2n):

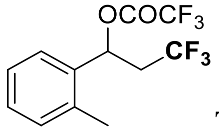

The reaction was carried out according to the general procedure. The target compound **2n** was obtained as a colorless oil (50 mg, 83% yield) after purification by column chromatography (SiO_2; 100% hexane).

^{1}H NMR (400 MHz, $CDCl_3$)

2.45 (s, 3H), 2.54 (dqd, *J* = 15.7, 10.1, 3.2, 1H), 2.92 (m, 1H), 6.43 (dd, *J* = 9.6, 3.2 Hz, 1H), 7.21 (m, 1H), 7.25-7.29 (overlap, 2H), 7.33 (m, 1H).

^{13}C NMR (100 MHz, $CDCl_3$)

19.0, 39.9 (q, *J* = 29 Hz), 70.7 (q, *J* = 2.8 Hz), 114.5 (q, *J* = 285 Hz), 124.9 (q, *J* = 277 Hz), 125.5, 127.2, 129.6, 131.2, 134.9, 135.1, 156.3 (q, *J* = 43 Hz).

^{19}F NMR (376 MHz, $CDCl_3$)

–64.8 (t, *J* = 10.1 Hz, 3F), –75.2 (s, 3F).

IR (neat, cm^{-1})

1792, 1333, 1252, 1222, 1133, 1099, 1064, 833, 761, 725, 615.

HRMS-EI (*m*/*z*)

[M] calcd. for $C_{12}H_{10}F_6O_2$, 300.0585; found, 300.0558.

Synthesis of 1-(2,6-dichlorophenyl)-3,3,3-trifluoropropyl 2,2,2-trifluoroacetate (2o):

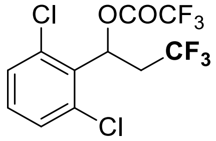

The reaction was carried out in DCE at 60 °C for the second step, but otherwise according to the general procedure. The desired compound **2o** was obtained as a colorless oil (53 mg, 75% yield) after purification by column chromatography (SiO_2; 100% hexane).

^{1}H NMR (400 MHz, $CDCl_3$)

2.77 (dqd, *J* = 15.5, 10.1, 4.0 Hz, 1H), 3.40 (m, 1H), 6.91 (dd, *J* = 8.8, 4.0 Hz, 1H), 7.27 (m, 1H), 7.37 (m, 2H).

^{13}C NMR (100 MHz, $CDCl_3$)

36.7 (q, *J* = 29 Hz), 70.1 (q, *J* = 2.9 Hz), 114.4 (q, *J* = 285 Hz), 124.9 (q, *J* = 277 Hz), 128.5, 130.7 (2C), 131.3 (2C), 135.6, 156.2 (q, *J* = 43 Hz).

^{19}F NMR (376 MHz, $CDCl_3$)

–64.8 (d, *J* = 10.1 Hz, 3F), –74.7 (s, 3F).

IR (neat, cm^{-1})

1793, 1566, 1441, 1395, 1352, 1318, 1284, 1249, 1226, 1196, 1139, 1093, 1076, 832, 782, 774, 740, 622.

HRMS-EI (*m*/*z*)

[M] calcd. for $C_{11}H_6Cl_2F_6O_2$, 353.9649; found, 353.9632.

Synthesis of 3,3,3-trifluoro-2-methyl-1-phenylpropyl 2,2,2-trifluoroacetate (2p):

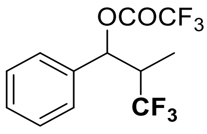

The reaction was carried out according to the general procedure. The target compound **2p** was obtained as a colorless oil (28 mg, 47% yield, *anti*:*syn* = 2:1) after purification by column chromatography (SiO_2; 100% hexane).[8]

^{1}H NMR (400 MHz, $CDCl_3$)

anti-isomer: 1.22 (d, *J* = 7.2 Hz, 3H), 2.68 (m, 1H), 6.36 (d, *J* = 3.2 Hz, 1H), 7.29 (m, 2H), 7.35–7.44 (overlap, 3H).

syn-isomer: 0.94 (d, *J* = 7.2 Hz, 3H), 2.93 (m, 1H), 5.97 (d, *J* = 9.6 Hz, 1H), 7.35–7.44 (overlap, 5H).

^{13}C NMR (100 MHz, $CDCl_3$)

anti-isomer: 7.0, 44.3 (q, *J* = 26 Hz), 75.6 (q, *J* = 2.8 Hz), 114.5 (q, *J* = 284 Hz), 125.7 (2C), 126.5 (q, *J* = 279 Hz), 129.1 (2C), 129.2, 135.8, 156.1 (q, *J* = 43 Hz).

syn-isomer: 10.7, 42.7 (q, *J* = 26 Hz), 78.1, 114.5 (q, *J* = 284 Hz), 126.7 (q, *J* = 279 Hz), 127.6 (2C), 129.2 (2C), 129.9, 135.0, 156.1 (q, *J* = 43 Hz).

---

[8]The stereochemistry was determined by comparison of ^{19}F NMR signals to those reported for the alcohols after hydrolysis of the products. For **2p**, **2r**, **2t** (a) Y. Yasu, T. Koike, M. Akita, *Angew. Chem. Int. Ed.*, 2012, **51**, 9567: For **2s**, (b) Y. Yang, Y. Liu, Y. Jiang, Y. Zhang, D. A. Vicic, *J. Org. Chem.*, 2015, **80**, 6639.

^{19}F NMR (376 MHz, $CDCl_3$)

anti-isomer: –70.7 (d, *J* = 8.6 Hz, 3F), –75.1 (s, 3F).

syn-isomer: –69.6 (d, *J* = 8.6 Hz, 3F), –75.3 (s, 3F).

IR (neat, cm^{-1})

1791, 1378, 1348, 1328, 1263, 1226, 1156, 1133, 1076, 1019, 773, 753, 732, 700, 668, 612.

HRMS-EI (*m/z*)

[M] calcd. for $C_{12}H_{10}F_6O_2$, 300.0585; found, 300.0566.

Synthesis of 3-oxo-1-phenyl-2-(trifluoromethyl)butyl 2,2,2-trifluoroacetate (2q):

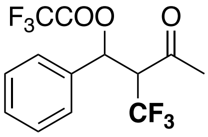

The reaction was carried out in DCE at 60 °C for the second step, but otherwise according to the general procedure. The target compound **2q** was obtained as a yellow oil (26 mg, 38% yield, *anti/syn* = 2:1) after purification by column chromatography (SiO_2; EtOAc/hexane = 5/95).[9]

^{1}H NMR (400 MHz, $CDCl_3$)

anti-isomer: 2.41 (s, 3H), 4.00 (m, 1H), 6.37 (d, *J* = 9.6 Hz, 1H), 7.42 (overlap, 5H).

syn-isomer: 1.95 (s, 3H), 4.07 (m, 1H), 6.36 (d, *J* = 10.8 Hz, 1H), 7.42 (overlap, 5H).

^{13}C NMR (100 MHz, $CDCl_3$)

anti-isomer: 32.3, 59.9 (q, *J* = 25 Hz), 76.5 (q, *J* = 1.9 Hz), 114.3 (q, *J* = 285 Hz), 122.6 (q, *J* = 279 Hz), 127.4 (2C), 129.3 (2C), 130.3, 133.8, 155.3 (q, *J* = 43 Hz), 198.3.

syn-isomer: 32.7, 60.0 (q, *J* = 25 Hz), 75.4, 114.3 (q, *J* = 285 Hz), 122.6 (q, *J*

[9]The stereochemistry was determined by comparison of ^{1}H NMR chemical shifts of the methyl groups due to magnetic shielding by the phenyl groups of each diastereomers, where the conformations were confirmed by NOESY correction as shown in the following scheme. In addition, relative chemical shifts of diastereomers of **2q** are similar to those of diastereomers of 3,3,3-trifluoro-2-methyl-1-phenylpropanol (ref 8a).

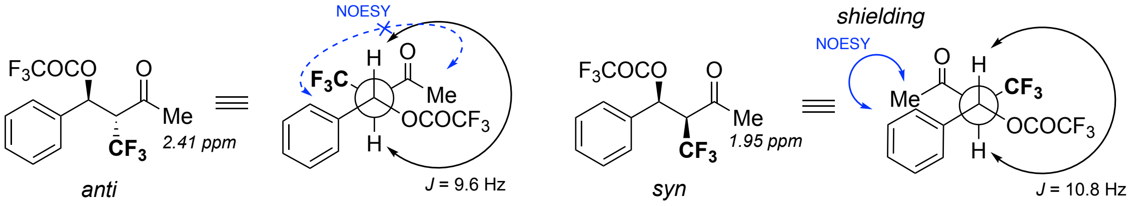

= 279 Hz), 127.7 (2C), 128.5, 129.5 (2C), 130.5, 155.3 (q, *J* = 43 Hz), 198.1.

^{19}F NMR (376 MHz, $CDCl_3$)

anti-isomer: –63.0 (d, *J* = 7.2 Hz, 3F), –75.1 (s, 3F).

syn-isomer: –63.9 (d, *J* = 7.2 Hz, 3F), –75.2 (s, 3F).

IR (neat, cm^{-1})

1799, 1734, 1363, 1344, 1328, 1260, 1231, 1207, 1171, 1143, 1017, 798, 698, 668.

HRMS-EI (*m*/*z*)

[M] calcd. for $C_{13}H_{10}F_6O_3$, 328.0534; found, 328.0502.

Synthesis of 2-(trifluoromethyl)-2,3-dihydro-1*H*-inden-1-yl 2,2,2-trifluoroacetate (2r):

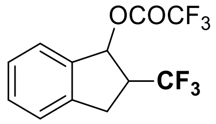

The reaction was carried out according to the general procedure. The target compound **2r** was obtained as a colorless oil (38 mg, 60% yield, *trans*:*cis* = 8:1) after purification by column chromatography (SiO_2; 100% hexane).[8]

^{1}H NMR (400 MHz, $CDCl_3$)

trans-isomer: 3.16 (dd, *J* = 16.0, 6.4 Hz, 1H), 3.35 (m, 1H), 3.43 (dd, *J* = 16.0, 8.8 Hz, 1H), 6.68 (d, *J* = 4.8 Hz, 1H), 7.30–7.37 (overlap, 3H), 7.41 (m, 1H).

cis-isomer: 3.16 (dd, *J* = 15.3, 7.8 Hz, 1H), 3.35 (m, 1H), 3.47 (dd, *J* = 15.3, 9.0 Hz, 1H), 6.53 (d, *J* = 6.0 Hz, 1H), 7.32–7.36 (overlap, 2H),7.43 (m, 1H), 7.50 (m, 1H).

^{13}C NMR (100 MHz, $CDCl_3$)

trans-isomer: 30.8 (q, *J* = 1.9 Hz), 49.1 (q, *J* = 28 Hz), 80.8 (q, *J* = 2.9 Hz), 114.6 (q, *J* = 285 Hz), 125.2, 125.4, 126.6 (q, *J* = 277 Hz), 128.3, 130.7, 137.0, 140.8, 157.2 (q, *J* = 43 Hz).

cis-isomer: 30.8 (q, *J* = 1.9 Hz), 46.7 (q, *J* = 29 Hz), 78.4 (q, *J* = 1.9 Hz), 114.5 (q, *J* = 286 Hz), 125.3, 125.5 (q, *J* = 277 Hz), 126.5, 128.2, 131.1, 136.8, 142.3, 157.0 (q, *J* = 43 Hz).

^{19}F NMR (376 MHz, $CDCl_3$)

trans-isomer: –70.7 (d, *J* = 8.6 Hz, 3F), –74.9 (s, 3F).

cis-isomer: –66.0 (d, *J* = 8.6 Hz, 3F), –75.1 (s, 3F).

IR (neat, cm^{-1})

1788, 1375, 1332, 1269, 1226, 1145, 1118, 927, 774, 751, 692, 617.

HRMS-EI (*m*/*z*)

[M] calcd. for $C_{12}H_8F_6O_2$, 298.0428; found, 298.0401.

Synthesis of 2-(trifluoromethyl)-1,2,3,4-tetrahydronaphthalene-1-yl 2,2,2-trifluoroacetate (2s):

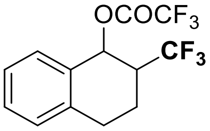

The reaction was carried out according to the general procedure. The target compound **2s** was obtained as a colorless oil (46 mg, 73% yield, *trans*:*cis* = 2:1) after purification by column chromatography (SiO_2; 100% hexane).[8]

^{1}H NMR (400 MHz, $CDCl_3$)

trans-isomer: 1.90 (m, 1H), 2.31 (m, 1H), 2.81–3.02 (overlap, 3H), 6.50 (d, *J* = 8.0 Hz, 1H), 7.17–7.20 (overlap, 2H), 7.24–7.32 (overlap, 2H).

cis-isomer: 2.16 (m. 1H), 2.26 (m, 1H), 2.70 (m, 1H), 2.96 (m, 1H), 3.13 (dd, *J* = 17.6, 6.0 Hz, 1H), 6.50 (d, *J* = 8.0 Hz, 1H), 7.23 (d, *J* = 6.8 Hz, 2H), 7.36 (m, 1H), 7.42 (d, *J* = 8.0 Hz, 1H).

^{13}C NMR (100 MHz, $CDCl_3$)

trans-isomer: 21.3 (q, *J* = 2.9 Hz), 27.3, 44.3 (q, *J* = 27 Hz), 72.6 (q, *J* = 1.9 Hz), 114.6 (q, *J* = 285 Hz), 126.5 (q, *J* = 278 Hz), 127.4, 128.2, 129.0, 129.4, 131.0, 137.3, 157.4 (q, *J* = 42 Hz).

cis-isomer: 17.1, 27.8, 43.4 (q, *J* = 28 Hz), 70.8 (q, *J* = 2.8 Hz), 114.6 (q, *J* = 285 Hz), 126.5 (q, *J* = 278 Hz), 127.2, 129.5, 130.4, 130.8 (2C), 136.6, 157.4 (q, *J* = 42 Hz).

^{19}F NMR (376 MHz, $CDCl_3$)

trans-isomer: –71.0 (d, *J* = 8.6 Hz, 3F), –75.1 (s, 3F).

cis-isomer: –69.5 (d, *J* = 8.6 Hz, 3F), –75.2 (s, 3F).

IR (neat, cm^{-1})

1787, 1324, 1263, 1224, 1148, 1128, 902, 830, 771, 750, 668.

HRMS-EI (*m*/*z*)

[M] calcd. for $C_{13}H_{10}F_6O_2$, 312.0585; found, 312.0563.

Synthesis of 1-phenyl-2-(trifluoromethyl)cyclohexyl 2,2,2-trifluoroacetate (2t):

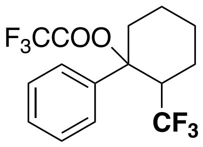

The reaction was carried out according to the general procedure. The desired compound **2t** was obtained as a colorless oil (40 mg, 59% yield, *trans*:*cis* = 1:2) after purification by column chromatography (SiO_2; 100% hexane).[8]

^{1}H NMR (400 MHz, $CDCl_3$)

cis-isomer: 1.78 (m, 2H), 2.13–2.39 (overlap, 5H), 3.87–3.97 (overlap, 2H), 7.36–7.47 (overlap, 3H), 7.69 (m, 2H).

trans-isomer: 1.97 (m, 2H), 2.13-2.39 (overlap, 3H), 2.51 (m, 1H), 2.98 (m, 1H), 3.64 (m, 2H), 7.36–7.47 (overlap, 5H).

^{13}C NMR (100 MHz, $CDCl_3$)

cis-isomer: 19.5, 21.6, 22.3, 23.1, 46.8 (q, *J* = 26 Hz), 87.1, 114.3 (q, *J* = 286 Hz), 125.4 (q, *J* = 278 Hz), 126.8 (2C), 128.2 (2C), 129.0, 134.4, 156.1 (q, *J* = 43 Hz).

trans-isomer: 21.0, 24.1, 27.5, 34.5, 46.1 (q, *J* = 24 Hz), 87.9, 114.4 (q, *J* = 286 Hz), 125.4 (q, *J* = 278 Hz), 126.4 (2C), 128.6 (2C), 129.2, 136.1, 156.1 (q, *J* = 43 Hz).

^{19}F NMR (376 MHz, $CDCl_3$)

cis-isomer: –65.2 (d, *J* = 8.6 Hz, 3F), –75.6 (s, 3F).

trans-isomer: –64.2 (d, *J* = 7.1 Hz, 3F), –75.2 (s, 3F).

IR (neat, cm^{-1})

1778, 1364, 1335, 1290, 1264, 1227, 1151, 1131, 1076, 986, 891, 838, 774, 764, 744, 706, 686, 638.

HRMS-EI (*m*/*z*)

[M] calcd. for $C_{15}H_{14}F_6O_2$, 340.0898; found, 340.0881.

Synthesis of 1-bromo-3,3,3-trifluoro-1-phenylpropyl 2,2,2-trifluoroacetate (2u):

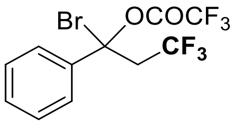

The reaction was carried out according to the general procedure. The target compound **2u** was obtained as a colorless oil (58 mg, 80% yield) after purification by column chromatography (SiO_2; 100% hexane).

^{1}H NMR (400 MHz, $CDCl_3$)

3.53 (dq, J = 15.6, 9.3 Hz, 1H), 4.18 (dq, J = 15.6, 9.3 Hz, 1H), 7.40–7.48 (overlap, 3H), 7.60 (m, 2H).

^{13}C NMR (100 MHz, $CDCl_3$)

47.7 (q, J = 29 Hz), 87.0 (q, J = 1.9 Hz), 114.0 (q, J = 285 Hz), 123.2 (q, J = 278 Hz), 125.0 (2C), 129.0 (2C), 130.2, 139.2, 153.9 (q, J = 44 Hz).

^{19}F NMR (376 MHz, $CDCl_3$)

–61.7 (t, J = 9.3 Hz, 3F), –75.2 (s, 3F).

IR (neat, cm^{-1})

1805, 1365, 1349, 1257, 1226, 1178, 1120, 874, 838, 799, 767, 735, 721, 690, 668, 624, 617.

HRMS-EI (*m*/*z*)

[M] calcd. for $C_9H_7F_3O$ (degradation product), 188.0449; found, 188.0432.

Synthesis of 1-(4-fluorophenyl)-3,3,4,4,4-pentafluorobutyl 2,2,3,3,3-pentafluoropropanoate (2b'):

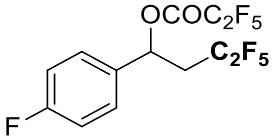

The reaction was carried out according to the general procedure. The target compound **2b'** was obtained as a colorless oil (75 mg, 93% yield) after purification by column chromatography (SiO_2; 100% hexane).

^{1}H NMR (400 MHz, $CDCl_3$)

2.53 (m, 1H), 2.91 (m, 1H), 6.32 (dd, J = 9.6, 3.2 Hz, 1H), 7.12 (m, 2H), 7.39 (m, 2H).

^{13}C NMR (100 MHz, $CDCl_3$)

37.1 (t, J = 21 Hz), 72.6 (br s), 100.0–130.0 (m, 4C),[10] 116.5 (d, J = 22 Hz, 2C), 128.5 (d, J = 8.6 Hz, 2C), 132.4 (d, J = 3.8 Hz), 157.1 (t, J = 30 Hz), 163.5 (d, J = 249 Hz).

^{19}F NMR (376 MHz, $CDCl_3$)

–82.6 (m, 3F), –85.7 (m, 3F), –110.4 (m, 1F), –117.4 (m, 2F), –121.7 (m, 2F).

IR (neat, cm^{-1})

1785, 1516, 1300, 1194, 1152, 1130, 1098, 1071, 1032, 838, 737, 668.

HRMS-EI (*m*/*z*)

[10]The carbons of perfluoroalkyl groups could not be assigned because of low intensity of signals, their complex coupling, and overlap due to large J values.

[M] calcd. for $C_{13}H_7F_{11}O_2$, 404.0270; found, 404.0248.

Synthesis of 1-(4-acetoxyphenyl)-3,3,4,4,5,5,5-heptafluoropentyl 2,2,3,3,4,4,4-heptafluorobutyrate (2d"):

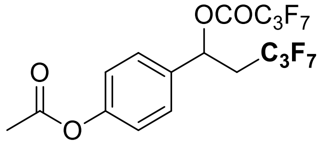

The reaction was carried out according to the general procedure. The desired compound **2d"** was obtained as a white solid (105 mg, quantitative yield) after purification by column chromatography (SiO_2; EtOAc/hexane = 5/95).

^{1}H NMR (400 MHz, $CDCl_3$)

2.31 (s, 3H), 2.57 (m, 1H), 2.94 (m, 1H), 6.36 (dd, J = 9.6, 2.8 Hz, 1H), 7.17 (d, J = 8.8 Hz, 2H), 7.42 (d, J = 8.8 Hz, 2H).

^{13}C NMR (100 MHz, $CDCl_3$)[10]

21.2, 37.1 (t, J = 22 Hz), 72.7, 100.0–130.0 (m, 6C),[10] 122.7 (2C), 127.8 (2C), 133.9, 151.8, 157.1 (t, J = 30 Hz), 169.4.

^{19}F NMR (376 MHz, $CDCl_3$)

–80.2 (m, 3F), –80.6 (m, 3F), –114.4 (m, 2F), –119.2 (m, 2F), –126.7 (m, 2F), –127.7 (m, 2F).

IR (neat, cm^{-1})

1783, 1354, 1301, 1217, 1147, 1117, 1083, 970, 939, 919, 725.

HRMS-EI (*m*/*z*)

[M] calcd. for $C_{17}H_{10}F_{14}O_4$, 544.0356; found, 544.0310.

Synthesis of 2-phenyl-1-tosyl-2-(2,2,2-trifluoroethyl)pyrrolidine (4a):

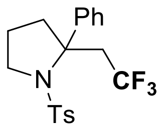

The reaction was carried out for 3 h at the second step, but otherwise according to the general procedure. The target compound **4a** was obtained as a white solid (58 mg, 76% yield) after purification by column chromatography (SiO_2; EtOAc/hexane/Et_3N = 3/96/1).

^{1}H NMR (400 MHz, $CDCl_3$)

1.96 (m, 1H), 2.06 (m, 1H), 2.38 (s, 3H), 2.44 (m, 2H), 3.44 (m, 2H), 3.55 (m, 1H), 3.71 (m, 1H), 7.10 (d, J = 8.4 Hz, 2H), 7.19–7.26 (overlap, 5H), 7.30 (m, 2H).

^{13}C NMR (100 MHz, $CDCl_3$)

21.6, 23.0, 41.1, 41.9 (q, J = 27 Hz), 49.6, 68.7 (q, J = 1.9 Hz), 126.1 (q, J = 277 Hz), 126.7 (2C), 127.0 (2C), 127.6, 128.3 (2C), 129.3 (2C), 137.5, 142.8, 142.9.

^{19}F NMR (376 MHz, $CDCl_3$)

–58.3 (t, J = 11.6 Hz).

IR (neat, cm^{-1})

1379, 1338, 1304, 1262, 1223, 1154, 1136, 1092, 1040, 974, 912, 813, 757, 733, 699, 659, 614.

HRMS-ESI (*m*/*z*)

[M+Na]$^+$ calcd. for $C_{19}H_{20}F_3NO_2SNa$, 406.1065; found, 406.1067.

Synthesis of (2*S*,3*R*)-2-phenyl-1-tosyl-3-(trifluoromethyl)pyrrolidine (4b):

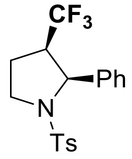

The reaction was carried out according to the general procedure. The target compound **4b** was obtained as a colorless oil (28 mg, 37% yield) after purification by column chromatography (SiO_2; EtOAc/hexane/Et_3N = 10/89/1).[11]

^{1}H NMR (400 MHz, $CDCl_3$)

1.96 (dddd, J = 13.6, 7.4, 4.5, 4.5 Hz, 1H), 2.22 (dddd, J = 13.6, 8.0, 8.0, 8.0 Hz, 1H), 2.43 (s, 3H), 2.76 (m, 1H), 3.52 (m, 1H), 3.74 (m, 1H), 4.87 (d, J = 3.6 Hz, 1H), 7.27–7.30 (overlap, 3H), 7.31–7.35 (overlap, 4H), 7.62 (d, J = 8.4 Hz, 2H).

^{13}C NMR (100 MHz, $CDCl_3$)

21.7, 24.5, 48.4, 52.7 (q, J = 27 Hz), 63.0 (q, J = 1.9 Hz), 126.2 (2C), 126.6 (q, J = 274 Hz), 127.7 (2C), 128.0, 128.9 (2C), 129.7 (2C), 134.4, 141.6, 143.9.

^{19}F NMR (376 MHz, $CDCl_3$)

–70.7 (d, J = 8.7 Hz).

IR (neat, cm^{-1})

1394, 1351, 1268, 1236, 1161, 1128, 1097, 1024, 1012, 815, 801, 783, 756, 700, 668, 619.

HRMS-ESI (*m*/*z*)

[11]The analytic and spectroscopic data matched reported values: Y. Wang, M. Jiang, J.-T. Liu, *Adv. Synth. Catal.*, 2016, **358**, 1322.

[M+Na]+ calcd. for $C_{18}H_{18}F_3NNaO_2S$, 392.0908; found, 392.0910.

Synthesis of 3-phenyl-2-tosyl-3-(2,2,2-trifluoroethyl)-2-azaspiro[4.5]decane (4c):

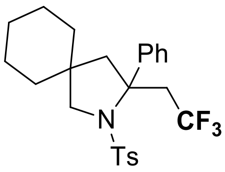

The reaction was carried out for 3 h for the second step, but otherwise according to the general procedure, on a 0.10 mmol scale. The target compound **4c** was obtained as a white solid (39 mg, 86% yield) after purification by column chromatography (SiO_2; EtOAc/hexane/Et_3N = 5/94/1). An analytical sample was obtained by crystallization from EtOAc.

^{1}H NMR (400 MHz, $CDCl_3$)

1.27–1.61 (overlap, 10H), 2.36 (s, 3H), 2.39 (d, J = 14.4 Hz, 1H), 2.46 (d, J = 14.4 Hz, 1H), 3.07 (dq, J = 16.1, 11.5 Hz, 1H), 3.14 (d, J = 9.6 Hz, 1H), 3.56 (d, J = 9.6 Hz, 1H), 4.01 (dq, J = 16.0, 11.2 Hz, 1H), 7.06 (d, J = 8.4 Hz, 2H), 7.14–7.22 (overlap, 5H), 7.32 (d, J = 8.4 Hz, 2H).

^{13}C NMR (100 MHz, $CDCl_3$)

21.6, 23.3, 23.8, 25.8, 37.1, 37.5, 40.6, 44.1 (q, J = 27 Hz), 51.6, 59.6, 69.0 (q, J = 1.9 Hz), 125.7 (q, J = 277 Hz), 127.0 (2C), 127.3 (2C), 127.6, 128.1 (2C), 129.2 (2C), 136.8, 142.2, 142.8.

^{19}F NMR (376 MHz, $CDCl_3$)

–56.3 (t, J = 11.5 Hz).

IR (neat, cm^{-1})

1448, 1340, 1257, 1212, 1156, 1121, 1090, 1054, 1034, 984, 925, 906, 813, 762, 732, 698, 661.

HRMS-ESI (*m/z*)

[M+Na]+ calcd. for $C_{24}H_{28}F_3NNaO_2S$, 474.1691; found, 474.1689.

Synthesis of 2-phenyl-1-tosyl-2-(2,2,3,3,3-pentafluoropropyl)pyrrolidine (4a'):

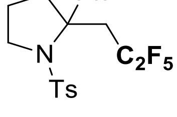

The reaction was carried out for 3 h for the second step, but otherwise according to the general procedure. The target compound **4a'** was obtained as a white solid (55 mg, 63% yield) after purification by column chromatography (SiO_2; EtOAc/hexane/Et_3N = 3/96/1).

^{1}H NMR (400 MHz, $CDCl_3$)

1.99 (m, 1H), 2.10 (m, 1H), 2.37 (s, 3H), 2.53 (m, 2H), 3.19 (m, 1H), 3.49-3.64 (overlap, 2H), 3.74 (m, 1H), 7.08 (d, *J* = 8.4 Hz, 2H), 7.17–7.24 (overlap, 5H), 7.30 (m, 2H).

^{13}C NMR (100 MHz, $CDCl_3$)[10]

21.6, 23.3, 37.8 (t, *J* = 19 Hz), 41.2, 49.5, 69.2, 110.0–130.0 (m, 2C),[10] 126.8 (2C), 126.9 (2C), 127.6, 128.3 (2C), 129.2 (2C), 137.5, 142.5, 142.9.

^{19}F NMR (376 MHz, $CDCl_3$)

–86.2 (m, 3F), –113.6 (m, 2F).

IR (neat, cm^{-1})

1341, 1198, 1155, 1134, 1093, 1056, 1020, 1008, 814, 755, 727, 699, 688, 659.

HRMS-ESI (*m*/*z*)

$[M+Na]^+$ calcd. for $C_{20}H_{20}F_5NO_2SNa$, 456.1033; found, 456.1032.

Synthesis of 2-phenyl-1-tosyl-2-(2,2,3,3,3-pentafluoropropyl)pyrrolidine (4a"):

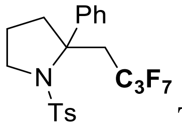

The reaction was carried out for 3 h for the second step, but otherwise according to the general procedure, on a 0.10 mmol scale. The target compound **4a"** was obtained as a white solid (32 mg, 65% yield) after purification by column chromatography (SiO_2; EtOAc/hexane/Et_3N = 5/94/1).

^{1}H NMR (400 MHz, $CDCl_3$)

1.99 (m, 1H), 2.09 (m, 1H), 2.37 (s, 3H), 2.53 (m, 2H), 3.20 (m, 1H), 3.52 (m, 1H), 3.63 (m, 1H), 3.73 (m, 1H), 7.08 (d, *J* = 8.4 Hz, 2H), 7.18–7.24 (overlap, 5H), 7.32 (m, 2H).

^{13}C NMR (100 MHz, $CDCl_3$)[10]

21.6, 23.3, 37.7 (t, *J* = 19 Hz), 41.1, 49.5, 69.3, 100.0–130.0 (m, 3C),[10] 126.8 (2C), 126.9 (2C), 127.6, 128.3 (2C), 129.2 (2C), 137.5, 142.4, 142.9.

^{19}F NMR (376 MHz, $CDCl_3$)

–79.9 (m, 3F), –110.2 (m, 2F), –127.4 (m, 2F).

IR (neat, cm^{-1})

1340, 1224, 1174, 1155, 1133, 1112, 1092, 1035, 911, 813, 755, 735, 699, 687, 666.

HRMS-ESI (*m*/*z*)

[M+Na]$^+$ calcd. for $C_{21}H_{20}F_7NNaO_2S$, 506.1001; found, 506.0999.

Synthesis of 1-(4-fluorophenyl)-3,3,3-trifluoropropan-1-ol (5b):

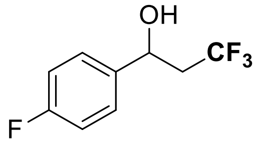

A solution of **2b** (100 mg, 0.33 mmol) in DME (1 mL) was cooled to 0 °C. 1,8-Diazabicyclo[5.4.0]undec-5-ene (DBU) (54 μL, 0.36 mmol) was added dropwise and the solution was stirred for 10 min at 0 °C.[12] The reaction mixture was then quenched with saturated NH_4Cl solution (2 mL) and extracted with Et_2O (3 x 5 mL). Filtration of the combined organic phase through a silica pad, followed by evaporation *in vacuo* gave a colorless oil. The crude product was purified by column chromatography (SiO_2; EtOAc/hexane = 10/90), providing the target compound **5b** as a colorless oil (41 mg, 96% yield).

^{1}H NMR (400 MHz, $CDCl_3$)

2.14 (br s, 1H), 2.43 (m, 1H), 2.62 (m, 1H), 5.08 (dd, J = 8.8, 3.6 Hz, 1H), 7.07 (t, J = 8.8 Hz, 2H), 7.36 (m, 2H).

^{13}C NMR (100 MHz, $CDCl_3$)

43.1 (q, J = 27 Hz), 68.3 (q, J = 2.9 Hz), 115.9 (d, J = 22 Hz, 2C), 125.9 (q, J = 277 Hz), 127.6 (d, J = 8.6 Hz, 2C), 138.2 (d, J = 2.9 Hz), 162.7 (d, J = 246 Hz).

^{19}F NMR (376 MHz, $CDCl_3$)

–63.6 (t, J = 10.1 Hz, 3F), –113.5 (m, 1F).

IR (neat, cm^{-1})

3404, 1607, 1511, 1431, 1375, 1326, 1259, 1227, 1202, 1131, 1093, 1015, 860, 841, 827, 800, 669, 652.

HRMS-EI (*m*/*z*)

[M] calcd. for $C_9H_8F_4O$, 208.0511; found, 208.0503.

[12]Moisture in DBU and/or DME may participate in the reaction.

Synthesis of (*E*)-β-trifluoromethyl-3-fluorostyrene (6b):

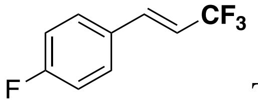

To a 0.07 M THF solution of KHMDS (0.4 mmol) was added **2b** (30.4 mg, 0.1 mmol) at –78 °C. The reaction mixture was stirred for 3.5 h, then quenched with saturated NH_4Cl solution (2 mL), and extracted with Et_2O (3 x 5 mL). The combined organic phase was dried over Na_2SO_4 and gently evaporated under vacuum (200 mmHg, 20 °C). The crude product was purified by means of column chromatography (SiO_2; 100% hexane) providing the desired compound **6b** as a colorless oil (12 mg, 63% yield).[13]

^{1}H NMR (400 MHz, $CDCl_3$)

6.13 (dq, *J* = 16.1, 6.5 Hz, 1H), 7.05–7.16 (m, 3H), 7.42–7.47 (m, 2H).

^{13}C NMR (100 MHz, $CDCl_3$)

115.8 (q, *J* = 32 Hz), 116.2 (d, *J* = 34.6 Hz, 2C), 123.7 (q, *J* = 269 Hz), 129.5 (d, *J* = 8.7 Hz, 2C), 129.8 (d, *J* = 3.9 Hz), 136.6 (q, *J* = 6.7 Hz), 163.9 (d, *J* = 250 Hz).

^{19}F NMR (376 MHz, $CDCl_3$)

–63.2 (d, *J* = 6.5 Hz, 3F), –110.2 (m, 1F).

Synthesis of 1-(3,3,3-trifluoro-1-(4-fluorophenyl)propyl)naphthalene-2-ol (7b):

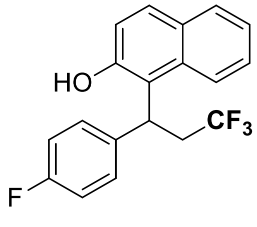

2-Naphthol (47 mg, 0.33 mmol) and trifluoromethanesulfonic acid (3 μL, 20 mol%) were added to a solution of **2b** (50 mg, 0.16 mmol) in HFIP (1.6 mL) under nitrogen in a Schlenk tube. The resulting solution was stirred at room temperature for 4 h, and then evaporated *in vacuo*. Purification by column chromatography (SiO_2; EtOAc/hexane = 10/90) provided the target compound **7b** as a white solid (37 mg, 68% yield).

^{1}H NMR (400 MHz, $CDCl_3$)

2.96 (m, 2H), 4.44 (t, *J* = 7.2 Hz, 1H), 4.91 (br, 1H), 6.99 (t, *J* = 8.8 Hz, 2H), 7.08–7.11 (overlap, 2H), 7.22-7.26 (overlap, 3H), 7.60–7.62 (overlap, 2H),

[13]The spectroscopic data obtained were in agreement with literature data: L. He, X. Yang, G. C. Tsui, *J. Org. Chem.*, 2017, *82*, 6192.

7.71 (m, 1H).

^{13}C NMR (100 MHz, $CDCl_3$)

39.7 (q, *J* = 27 Hz), 44.3 (q, *J* = 2.8 Hz), 109.5, 115.7 (d, *J* = 22 Hz, 2C), 118.3, 125.6, 126.4 (q, *J* = 277 Hz), 126.7, 127.3, 129.0, 129.3 (d, *J* = 8.7 Hz, 2C), 129.9, 133.6, 137.8, 138.5 (d, *J* = 2.8 Hz), 153.6, 161.8 (d, *J* = 245 Hz).

^{19}F NMR (376 MHz, $CDCl_3$)

–63.4 (t, *J* = 10.1 Hz, 3F), –115.9 (m, 1F).

IR (neat, cm^{-1})

3340, 1607, 1508, 1379, 1265, 1226, 1174, 1160, 1133, 1107, 1088, 861, 833, 820, 668, 653.

HRMS-EI (*m*/*z*)

[M] calcd. for $C_{19}H_{14}F_4O$, 334.0981; found, 334.0969.

Synthesis of 1,4-dimethyl-2-(3,3,3-trifluoro-1-(4-fluorophenyl)propyl)benzene (8b):

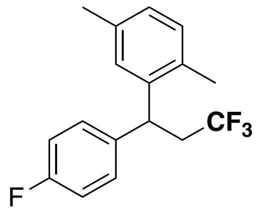

p-Xylene (31 μL, 0.25 mmol) and trifluoromethanesulfonic acid (1.5 μL, 20 mol%) were added to a solution of **2b** (25 mg, 0.08 mmol) in HFIP (0.8 mL) under nitrogen in a Schlenk tube. The resulting solution was stirred at room temperature for 3 h, and then evaporated *in vacuo*. Purification by column chromatography (SiO_2; 100% hexane) provided the target compound **8b** as a colorless oil (22 mg, 92% yield).

^{1}H NMR (400 MHz, $CDCl_3$)

2.27 (s, 3H), 2.34 (s, 3H), 2.84 (m, 2H), 4.51 (t, *J* = 7.2 Hz, 1H), 6.95–7.01 (overlap, 3H), 7.03–7.05 (overlap, 2H), 7.20 (m, 2H).

^{13}C NMR (100 MHz, $CDCl_3$)

19.4, 21.4, 39.9, 40.0 (q, *J* = 27 Hz), 115.5 (d, *J* = 21 Hz, 2C), 126.6 (q, *J* = 277 Hz), 127.0, 127.7, 129.6 (d, *J* = 7.7 Hz, 2C), 131.0, 132.7, 135.9, 138.0 (d, *J* = 2.9 Hz), 140.5, 161.6 (d, *J* = 245 Hz).

^{19}F NMR (376 MHz, $CDCl_3$)

–63.6 (t, *J* = 10.1 Hz, 3F), –116.1 (m, 1F).

IR (neat, cm^{-1})

1605, 1509, 1441, 1376, 1318, 1290, 1263, 1229, 1159, 1133, 1085, 1015, 837,

813, 784, 667, 620.

HRMS-EI (*m*/*z*)

[M] calcd. for $C_{17}H_{16}F_4$, 296.1188; found, 296.1176.

Synthesis of 1-fluoro-4-(1,1,1-trifluorohex-5-en-3-yl)benzene (9b):

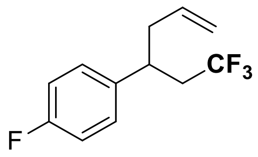

Allyltrimethylsilane (78 μL, 0.49 mmol) and **2b** (100 mg, 0.33 mmol) were added to a solution of tris(pentafluorophenyl)borane (17 mg, 10 mol%) in DCM (1 mL) under nitrogen. The resulting solution was stirred at room temperature for 48 h, then passed through a silica gel pad and concentrated under *vacuo*. Purification of the residue by column chromatography (SiO_2; 100% hexane) provided the target compound **9b** as a colorless oil (48 mg, 63% yield).

^{1}H NMR (400 MHz, $CDCl_3$)

2.29–2.55 (overlap, 4H), 3.02 (m, 1H), 5.02 (overlap, 2H), 5.60 (m, 1H), 7.00 (t, *J* = 8.8 Hz, 2H), 7.13 (m, 2H).

^{13}C NMR (100 MHz, $CDCl_3$)

39.0 (q, *J* = 1.9 Hz), 39.5 (q, *J* = 27 Hz), 41.1, 115.5 (d, *J* = 21 Hz, 2C), 117.8, 126.7 (q, *J* = 277 Hz), 128.9 (d, *J* = 7.6 Hz, 2C), 135.1, 138.6 (d, *J* = 2.9 Hz), 161.8 (d, *J* = 244 Hz).

^{19}F NMR (376 MHz, $CDCl_3$)

–63.5 (t, *J* = 10.1 Hz, 3F), –116.0 (m, 1F).

IR (neat, cm^{-1})

1606, 1512, 1378, 1256, 1226, 1161, 1143, 1122, 1093, 1053, 1015, 994, 920, 830, 742, 723, 668, 644, 626.

HRMS-EI (*m*/*z*)

[M] calcd. for $C_{12}H_{12}F_4$, 232.0875; found, 232.0866.

Synthesis of methyl 5,5,5-trifluoro-3-(4-fluorophenyl)-2,2-dimethylpentanoate (10b):

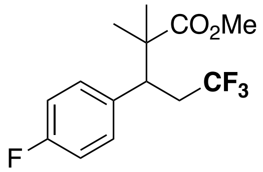

Methyl trimethylsilyl dimethylketene acetal (100 µL, 0.49 mmol) and **2b** (50 mg, 0.16 mmol) were added to a solution of tris(pentafluorophenyl)borane (8.4 mg, 10 mol%) in DCM (0.6 mL). The resulting solution was stirred at reflux for 6 h, passed through a silica gel pad and concentrated *in vacuo*. Purification of the residue by column chromatography (SiO_2; EtOAc/hexane = 1/99) provided the target compound **10b** as a colorless oil (24 mg, 52% yield).

^{1}H NMR (400 MHz, $CDCl_3$)

1.08 (s, 3H), 1.15 (s, 3H), 2.42 (m, 1H), 2.62 (m, 1H), 3.27 (dd, *J* = 11.2, 2.0 Hz, 1H), 3.67 (s, 3H), 7.00 (t, *J* = 8.8 Hz, 2H), 7.14 (m, 2H).

^{13}C NMR (100 MHz, $CDCl_3$)

21.5, 24.4, 35.7 (q, *J* = 27 Hz), 46.3 (q, *J* = 1.9 Hz), 46.6, 52.2, 115.2 (d, *J* = 21 Hz, 2C), 126.8 (q, *J* = 277 Hz), 130.7 (d, *J* = 7.7 Hz, 2C), 134.5 (d, *J* = 3.8 Hz), 162.2 (d, *J* = 245 Hz), 177.0.

^{19}F NMR (376 MHz, $CDCl_3$)

–64.0 (t, *J* = 10.1 Hz, 3F), –115.3 (m, 1F).

IR (neat, cm^{-1})

1729, 1512, 1436, 1392, 1325, 1303, 1294, 1256, 1227, 1192, 1138, 1125, 1113, 1090, 1051, 1015, 840, 827, 799, 633.

HRMS-EI (*m*/*z*)

[M] calcd. for $C_{14}H_{16}F_4O_2$, 292.1086; found, 292.1071.

TEMPO trapping test (Scheme 7b)

The reaction was carried out according to the general procedure with the addition of TEMPO (1 equiv. versus the styrene) before the substrate. For neutralization, saturated K_2CO_3 solution (1.5 mL) was added in addition to 0.5 M $NaHCO_3$ solution in order to recover TEMPO derivatives completely. The yields of the oxytrifluoromethylated products shown in Scheme 7b were estimated based on ^{19}F NMR analysis of the crude product.[14]

[14]The structures of **11** and **5a** were identified by comparison of the spectral data with literature values after rough isolation by means of column chromatography: (a) Y. Li, A. Studer, *Angew. Chem. Int. Ed.*, 2012, **51**, 8221. (b) Y. Yasu, T. Koike, M. Akita, *Angew. Chem. Int. Ed.*, 2012, **51**, 9567.

***Radical probe test using 12 (Scheme 8)*:**

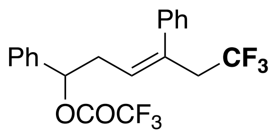

The reaction of **12** was carried out according to the general procedure (the ^{19}F NMR spectrum of the crude product is shown in Figure S4). Compound **13** was isolated as a colorless oil (25 mg, 31% yield, *E*/*Z* = 4/96) after purification by column chromatography (SiO_2; EtOAc/hexane = 4/96). Stereochemistry of isomers was determined by means of a NOESY experiment.

^1H NMR (400 MHz, $CDCl_3$)

Z-isomer: 2.84 (m, 1H), 3.01 (m, 1H), 3.16–3.36 (m, 2H), 5.87 (t, *J* = 7.9 Hz, 1H), 6.00 (dd, *J* = 7.9, 5.8 Hz, 1H), 7.20–7.48 (m, 10H).

E-isomer: 2.52–2.63 (m, 1H), 2.65–2.77 (m, 1H), 3.08 (overlap, 2H), 5.68 (t, *J* = 7.3 Hz, 1H), 5.90 (overlap, 1H), 7.20–7.48 (m, 10H).

^{13}C NMR (100 MHz, $CDCl_3$; *Z*-isomer)

35.0 (q, *J* = 29.9 Hz), 36.0, 79.6, 114.6 (q, *J* = 286 Hz), 125.8 (q, *J* = 278 Hz), 126.5 (2C), 126.6 (2C), 127.9, 128.6, 128.7 (2C), 129.1 (2C), 129.3, 133.9 (q, *J* = 2.9 Hz), 137.4, 141.5, 156.8 (q, *J* = 43 Hz).

^{19}F NMR (376 MHz, $CDCl_3$)

Z-isomer: –63.4 (t, *J* = 10.1 Hz, 3F), –75.0 (s, 3F).

E-isomer: –64.4 (t, *J* = 10.1 Hz, 3F), –175.0 (s, 3F).

IR (neat, cm^{-1})

3067, 1784, 1496, 1457, 1383, 1331, 1253, 1223, 1153, 1113, 759, 698.

HRMS-EI (*m*/*z*)

[M] calcd. for $C_{20}H_{16}F_6O_2$, 402.1054; found, 402.1061.

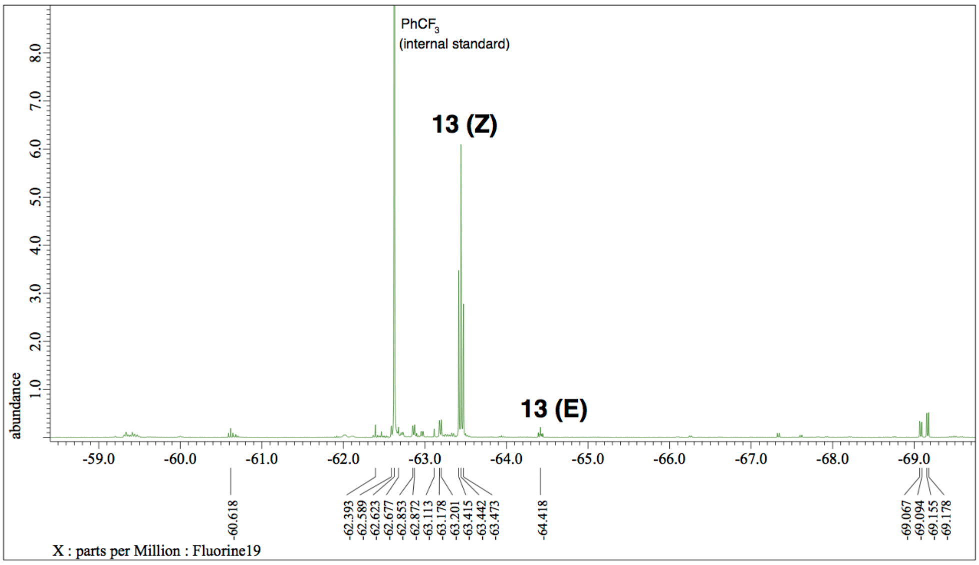

Figure S4. **^{19}F NMR spectrum of the crude product of the radical probe experiment using 12**

4. ^{1}H and ^{13}C NMR spectra of new compounds

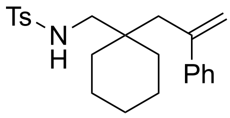

(**3c**)

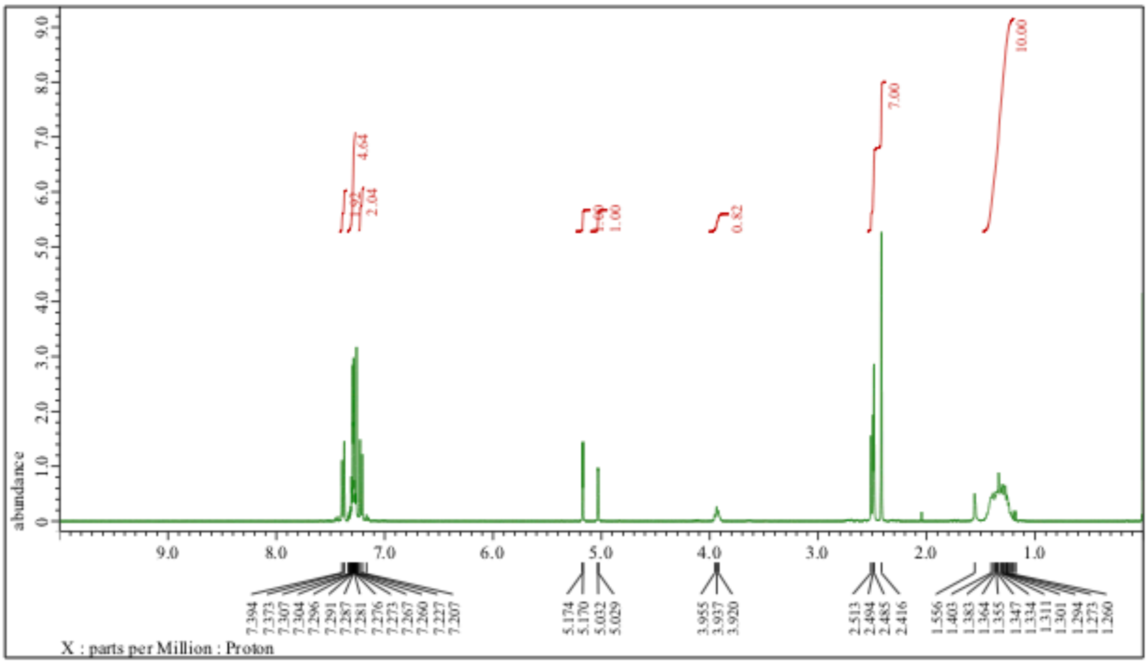

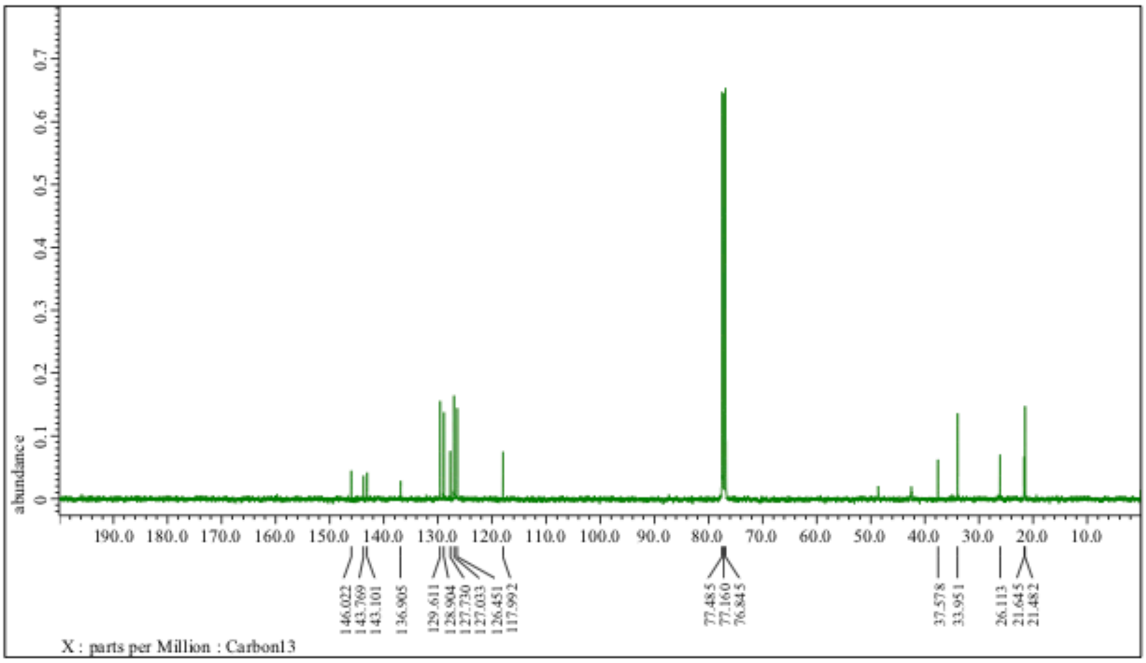

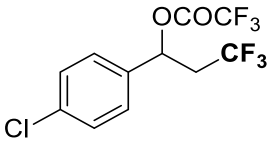

OCOCF3
CF3
Cl

(**2a**)

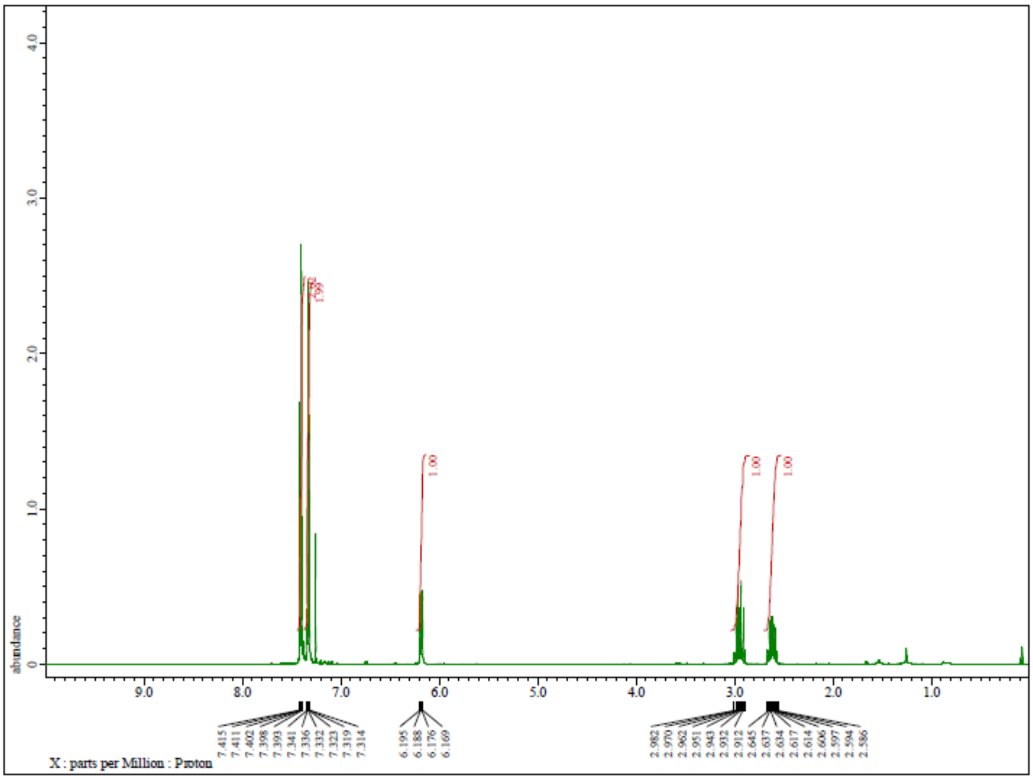

abundance
X : parts per Million : Proton
7.415 7.411 7.402 7.398 7.393 7.341 7.336 7.332 7.323 7.319 7.314
6.195 6.188 6.176 6.169
2.982 2.970 2.962 2.951 2.943 2.932 2.912 2.645 2.637 2.634 2.617 2.614 2.606 2.597 2.594 2.586

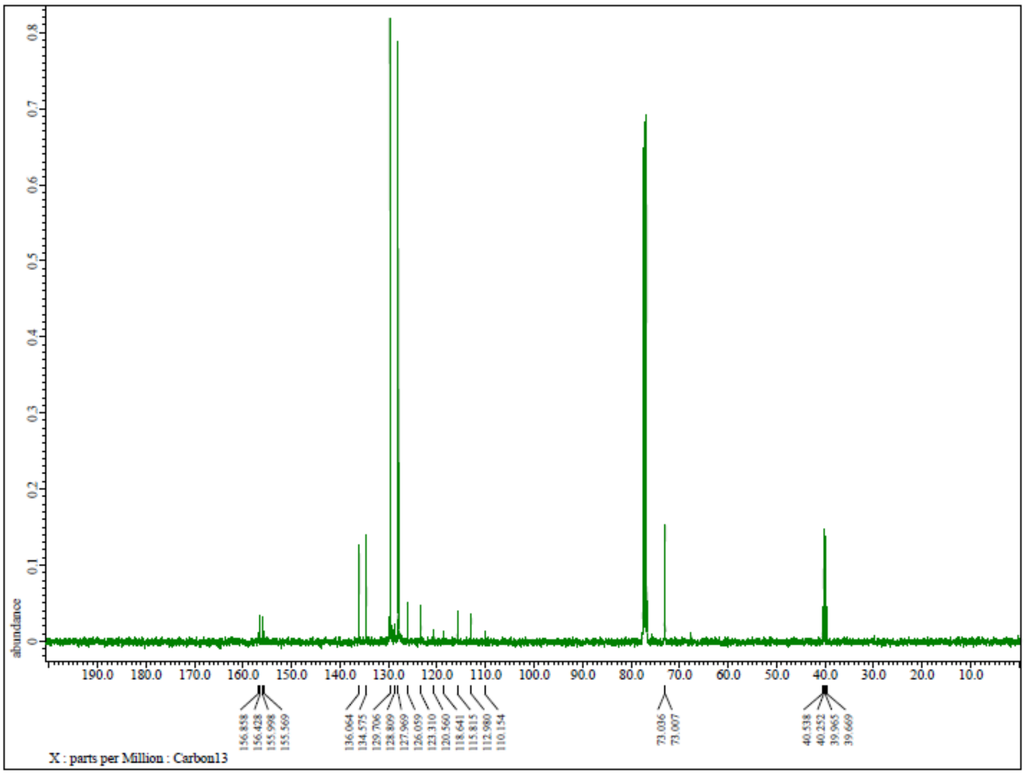

abundance
X : parts per Million : Carbon13
156.858 156.428 155.998 155.569
136.064 134.575 129.706 128.809 127.969 126.059 123.310 120.560 118.641 115.815 112.980 110.154
73.036 73.007
40.538 40.252 39.965 39.669

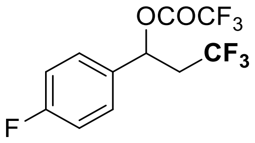
OCOCF3
CF3
F

(**2b**)

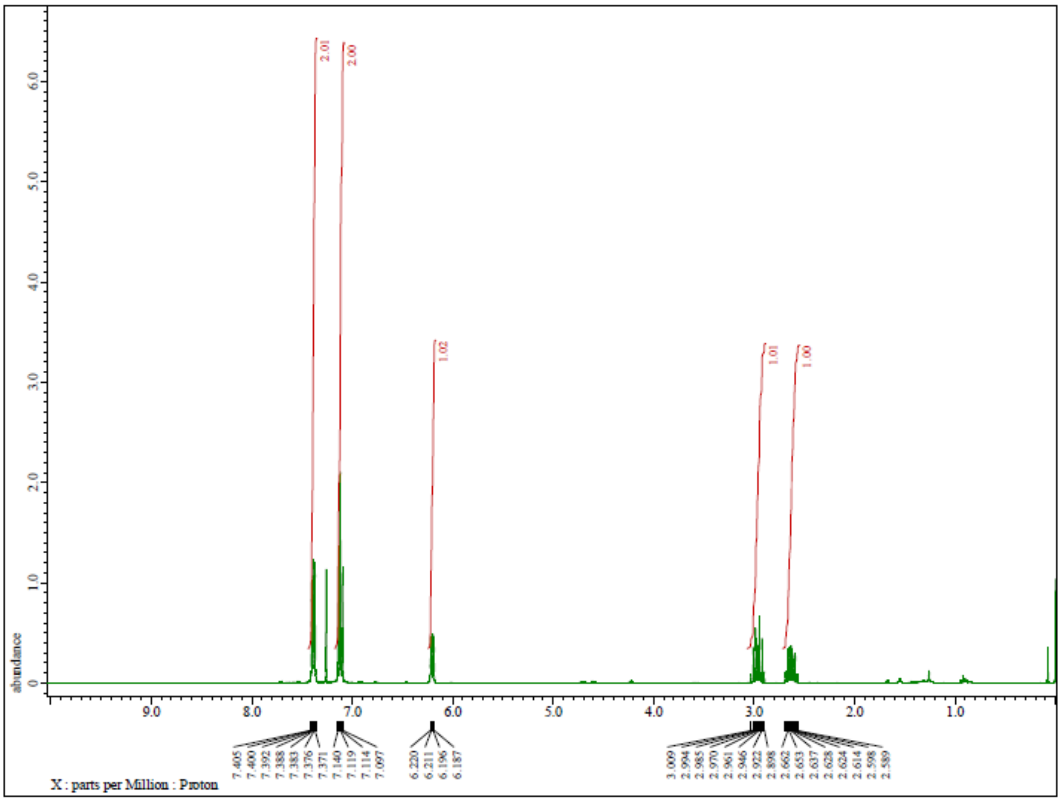
2.01
2.00
1.02
1.01
1.00
abundance
9.0 8.0 7.0 6.0 5.0 4.0 3.0 2.0 1.0
7.405 7.400 7.392 7.388 7.383 7.376 7.371 7.140 7.119 7.114 7.097
6.220 6.211 6.196 6.187
3.009 2.994 2.985 2.970 2.961 2.946 2.922 2.898 2.662 2.653 2.637 2.628 2.624 2.614 2.598 2.589
X : parts per Million : Proton

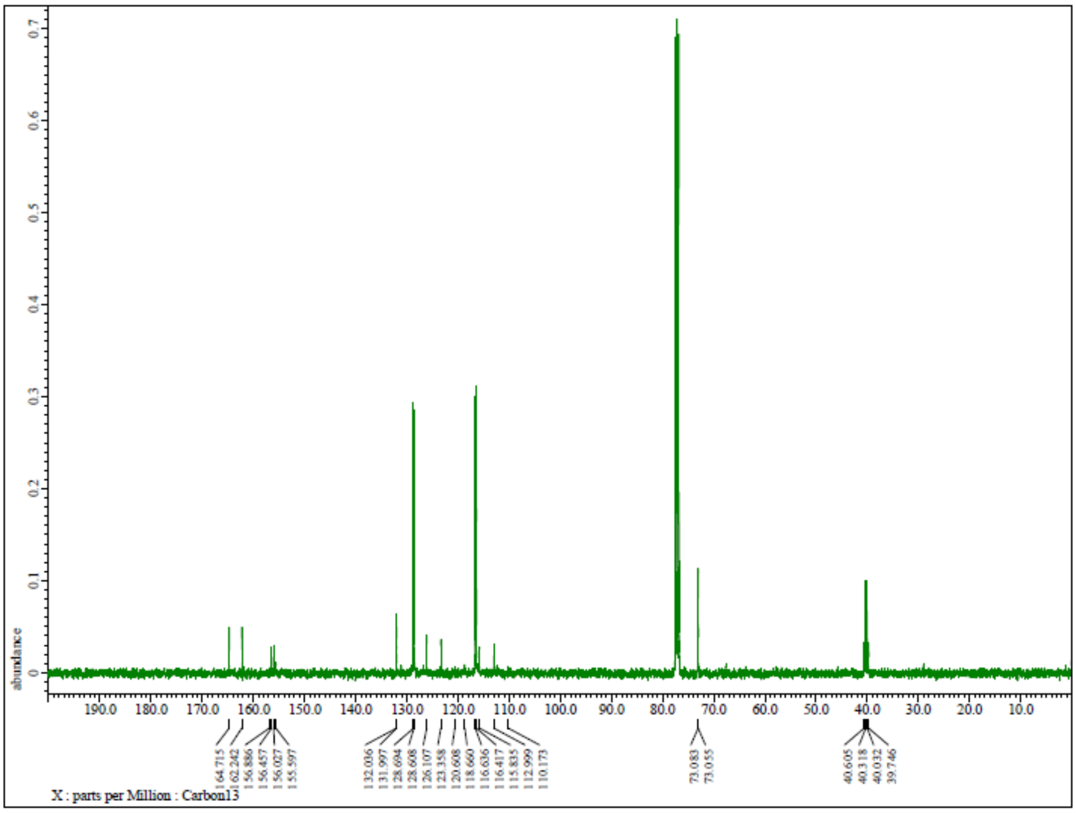
abundance
190.0 180.0 170.0 160.0 150.0 140.0 130.0 120.0 110.0 100.0 90.0 80.0 70.0 60.0 50.0 40.0 30.0 20.0 10.0
164.715 162.242 156.886 156.457 156.027 155.597
132.036 131.997 128.694 128.608 126.107 123.358 120.608 118.660 116.636 116.417 115.835 112.999 110.173
73.083 73.055
40.605 40.318 40.032 39.746
X : parts per Million : Carbon13

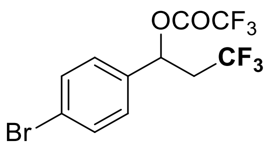
OCOCF3
CF3
Br

(**2c**)

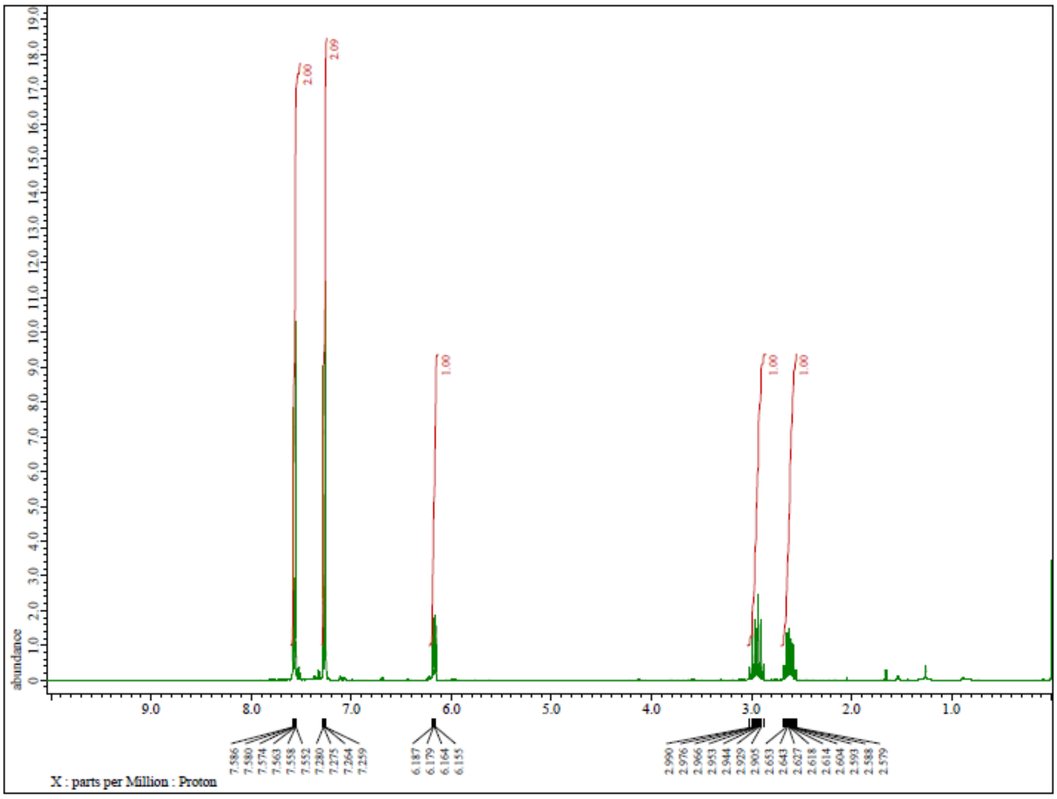
abundance
2.00
2.09
1.00
1.00
1.00
9.0
8.0
7.0
6.0
5.0
4.0
3.0
2.0
1.0
7.586
7.580
7.574
7.563
7.558
7.552
7.280
7.275
7.264
7.259
6.187
6.179
6.164
6.155
2.990
2.976
2.966
2.953
2.944
2.929
2.905
2.653
2.643
2.627
2.618
2.614
2.604
2.593
2.588
2.579
X : parts per Million : Proton

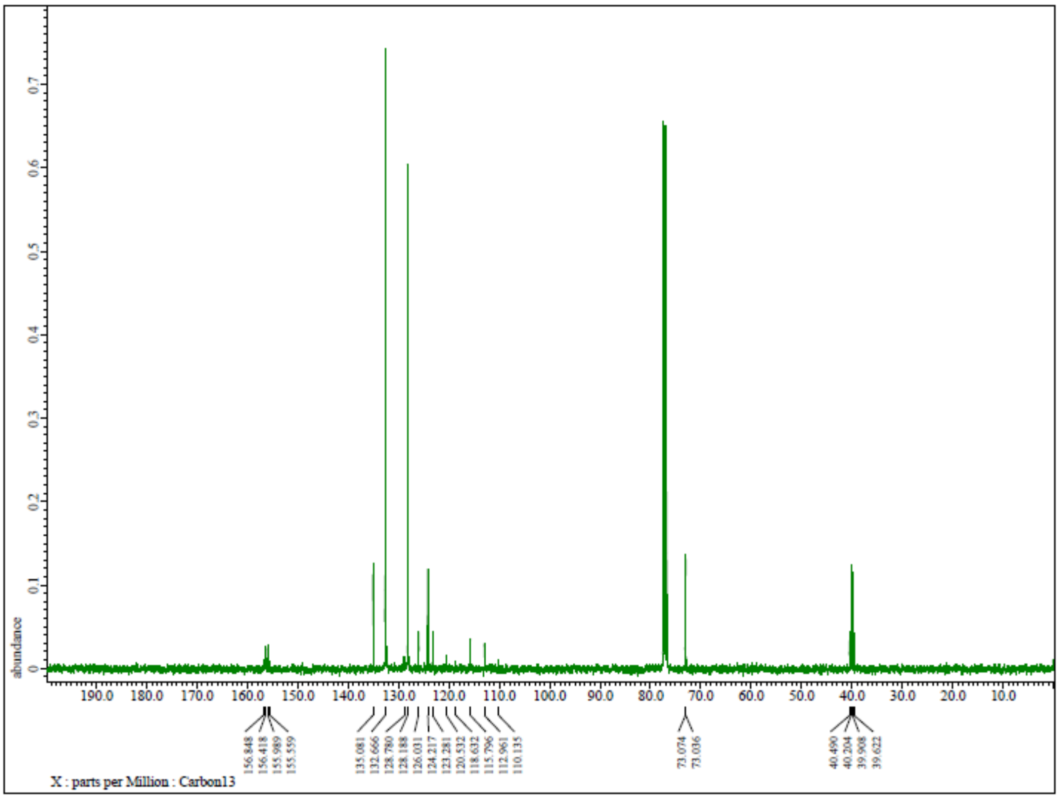
abundance
156.848
156.418
155.989
155.559
135.081
132.666
128.780
128.188
126.031
124.217
123.281
120.532
118.632
115.796
112.961
110.135
73.074
73.036
40.490
40.204
39.908
39.622
X : parts per Million : Carbon13

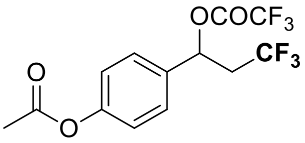

OCOCF3
CF3
O
O

(**2d**)

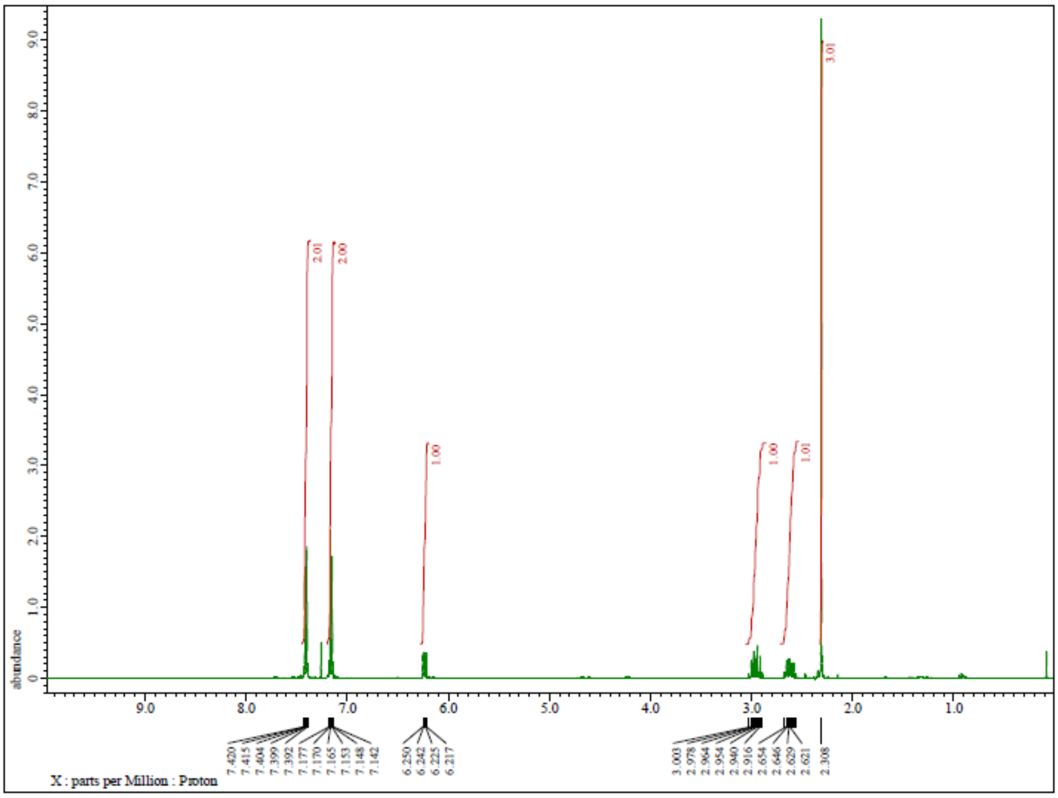

abundance
2.01
2.00
1.00
1.00
1.01
3.01
7.420
7.415
7.404
7.399
7.392
7.177
7.170
7.165
7.153
7.148
7.142
6.250
6.242
6.225
6.217
3.003
2.978
2.964
2.954
2.940
2.916
2.654
2.646
2.629
2.621
2.308
X : parts per Million : Proton

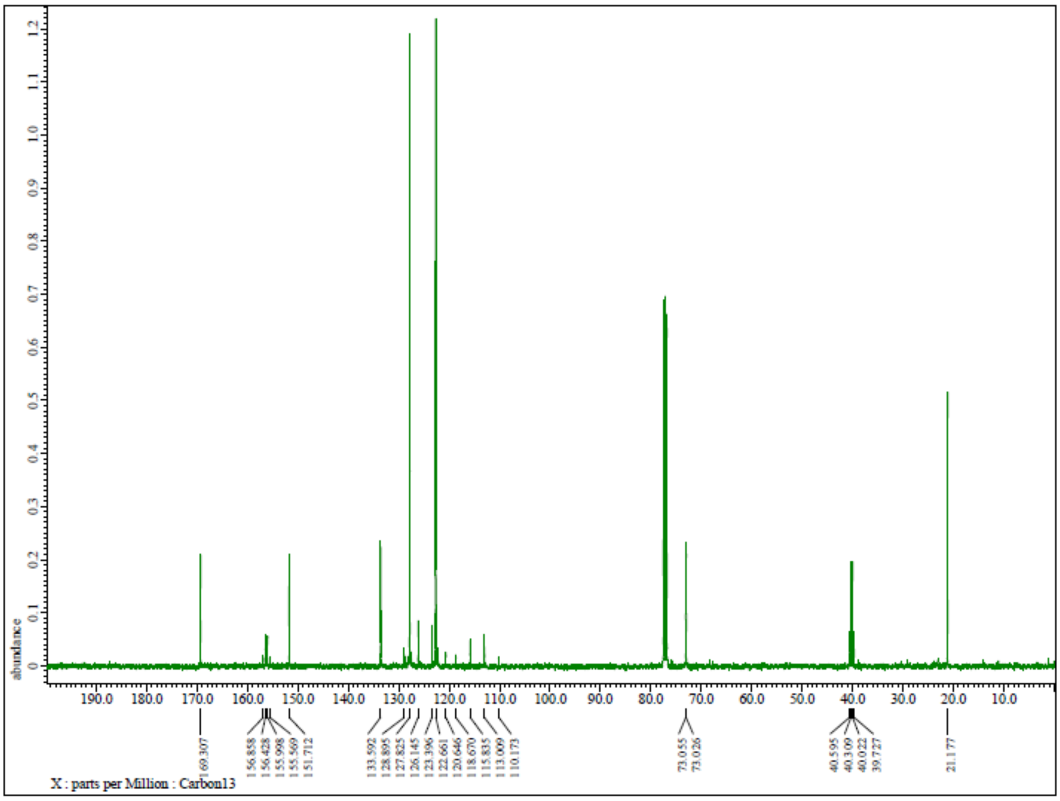

abundance
169.307
156.858
156.428
155.998
155.569
151.712
133.592
128.895
127.825
126.145
123.396
122.661
120.646
118.670
115.835
113.009
110.173
73.055
73.026
40.595
40.309
40.022
39.727
21.177
X : parts per Million : Carbon13

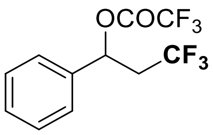
OCOCF3
CF3

(**2e**)

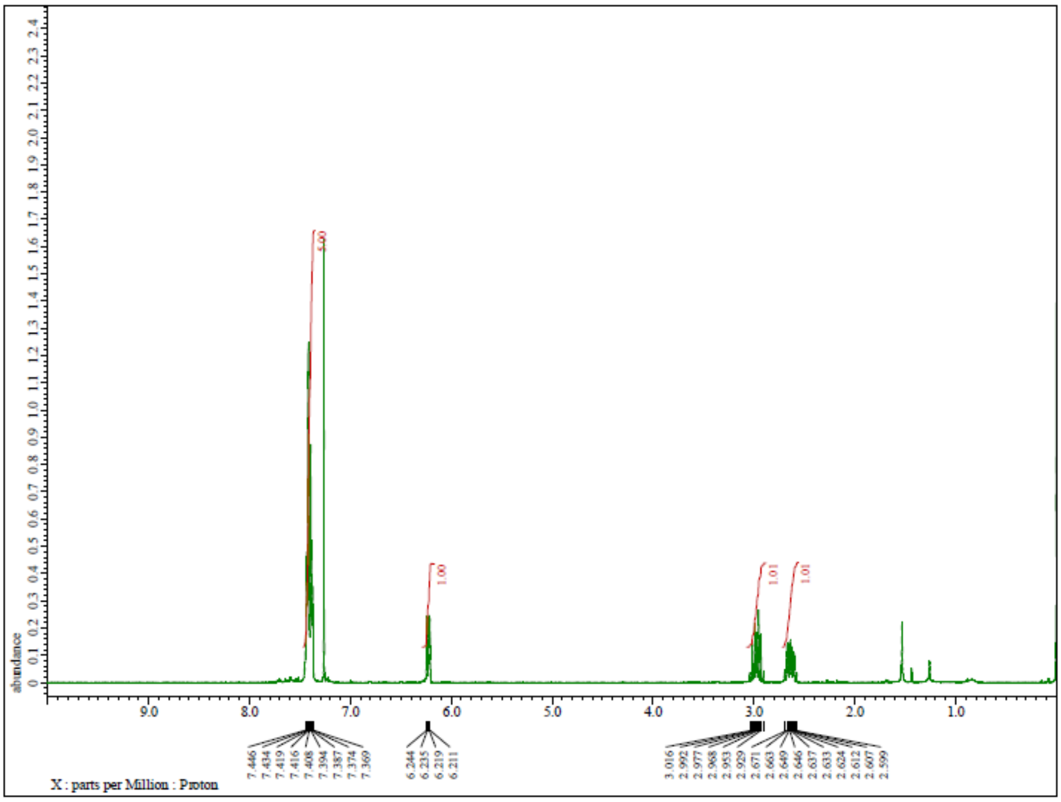
abundance
9.0
8.0
7.0
6.0
5.0
4.0
3.0
2.0
1.0
5.00
1.00
1.01
1.01
7.446 7.434 7.419 7.416 7.408 7.394 7.387 7.374 7.369
6.244 6.235 6.219 6.211
3.016 2.992 2.977 2.968 2.953 2.929 2.671 2.663 2.649 2.646 2.637 2.633 2.624 2.612 2.607 2.599
X : parts per Million : Proton

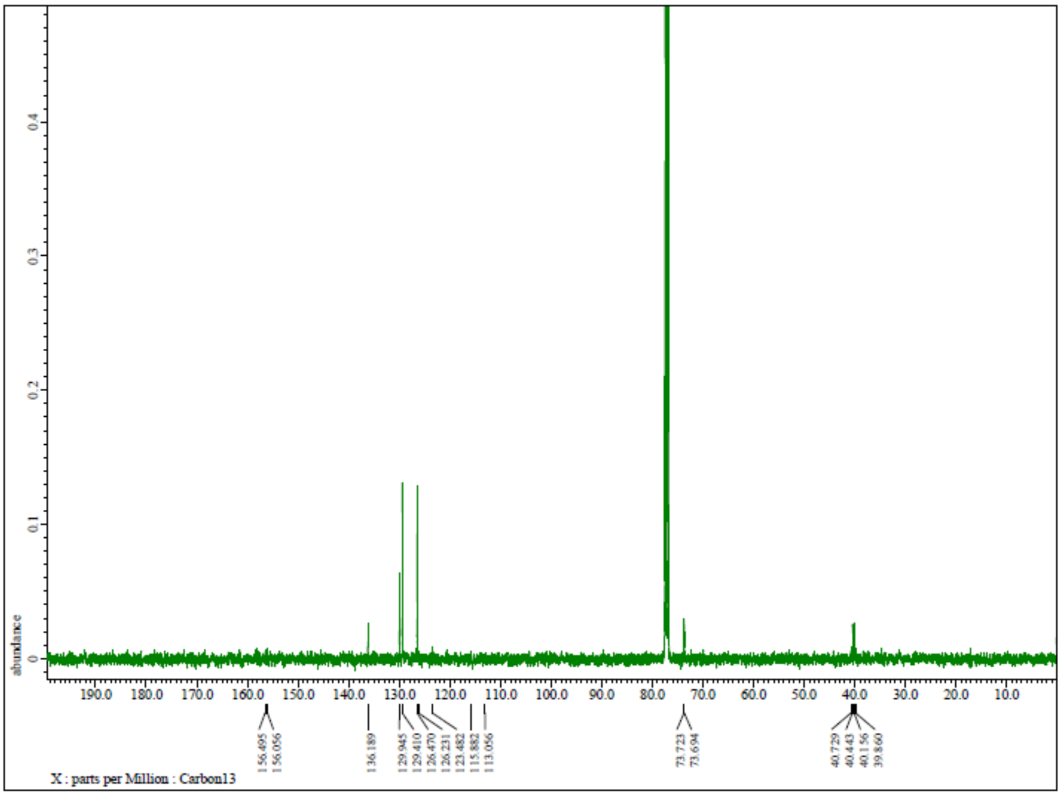
abundance
190.0
180.0
170.0
160.0
150.0
140.0
130.0
120.0
110.0
100.0
90.0
80.0
70.0
60.0
50.0
40.0
30.0
20.0
10.0
156.495 156.056
136.189
129.945 129.410 126.470 126.231 123.482 115.882 113.056
73.723 73.694
40.729 40.443 40.156 39.860
X : parts per Million : Carbon13

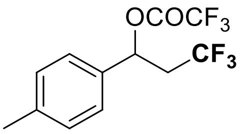
OCOCF3
CF3

(**2f**)

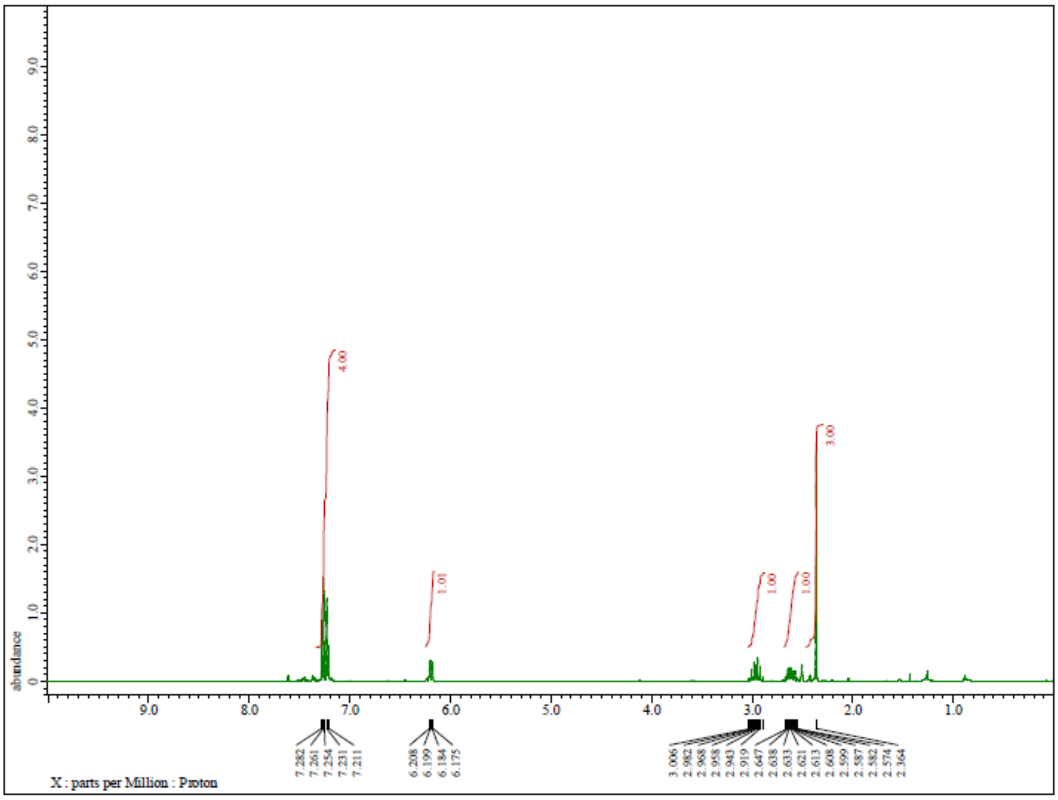
abundance
4.00
1.01
1.00
1.00
3.00
7.282
7.261
7.254
7.231
7.211
6.208
6.199
6.184
6.175
3.006
2.982
2.968
2.958
2.943
2.919
2.647
2.638
2.633
2.621
2.613
2.608
2.599
2.587
2.582
2.574
2.364
X : parts per Million : Proton

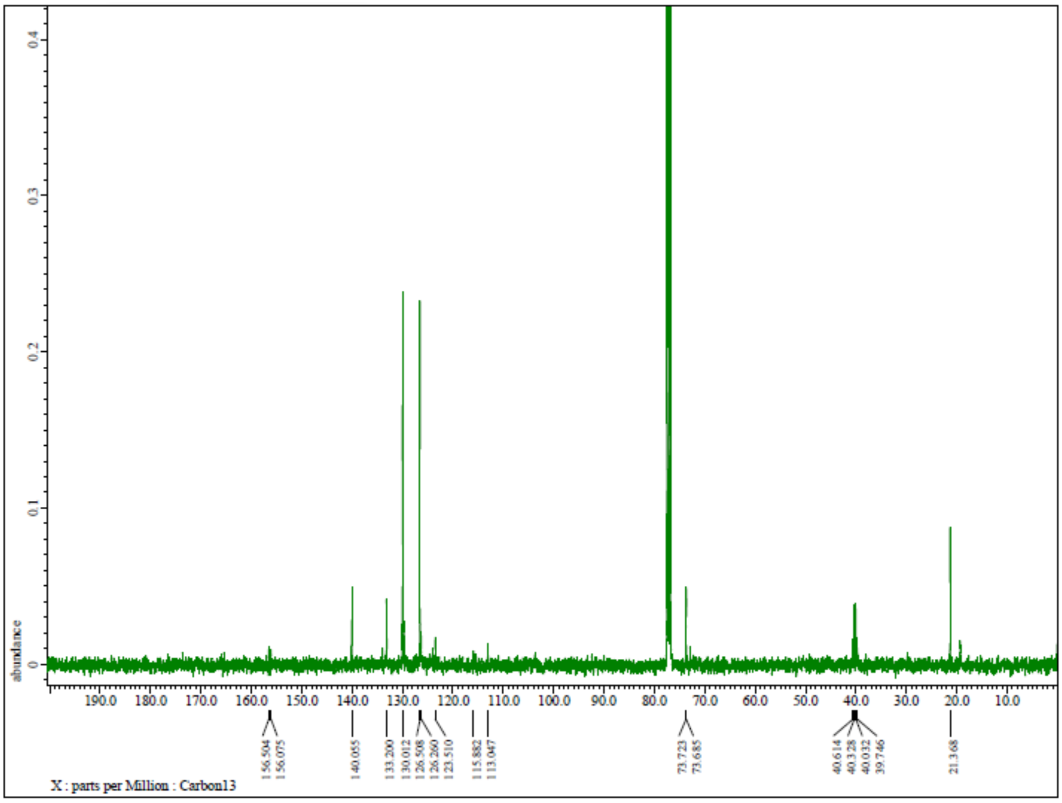
abundance
156.504
156.075
140.055
133.200
130.012
126.508
126.260
123.510
115.882
113.047
73.723
73.685
40.614
40.328
40.032
39.746
21.368
X : parts per Million : Carbon13

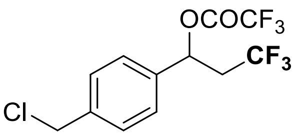
OCOCF3
CF3
Cl

(**2g**)

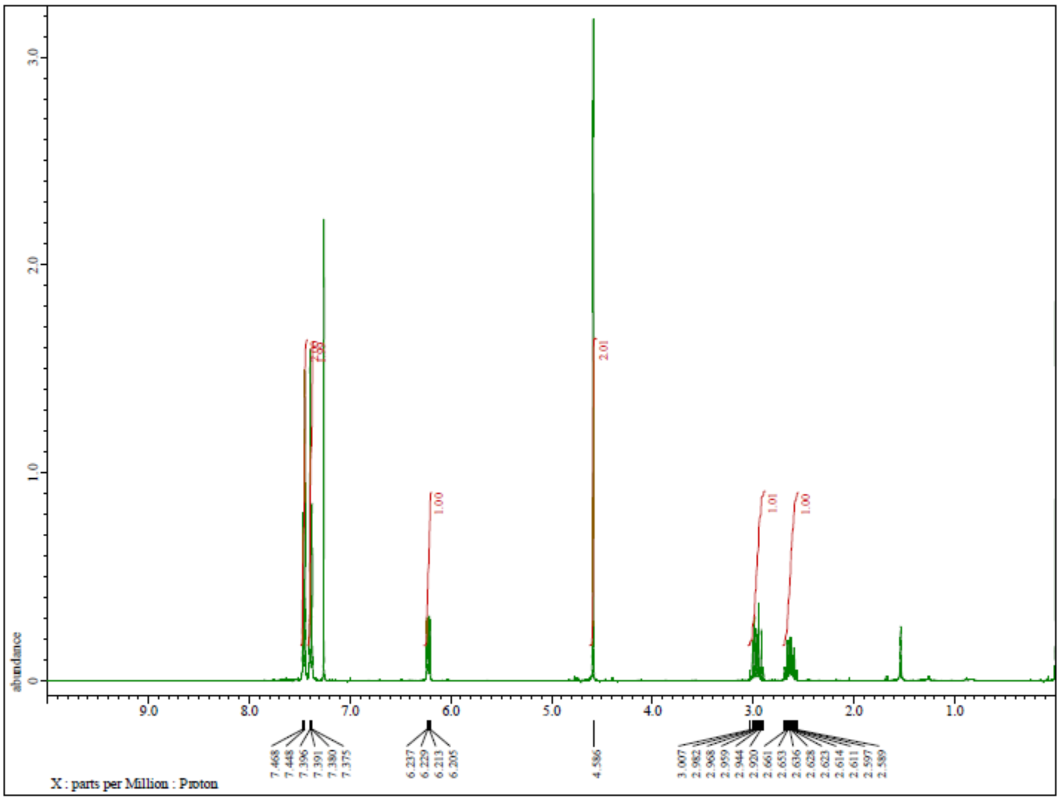
abundance
X : parts per Million : Proton
7.468 7.448 7.396 7.391 7.380 7.375
6.237 6.229 6.213 6.205
4.586
3.007 2.982 2.968 2.959 2.944 2.920 2.661 2.653 2.636 2.628 2.623 2.614 2.611 2.597 2.589
1.00
2.01
1.01
1.00

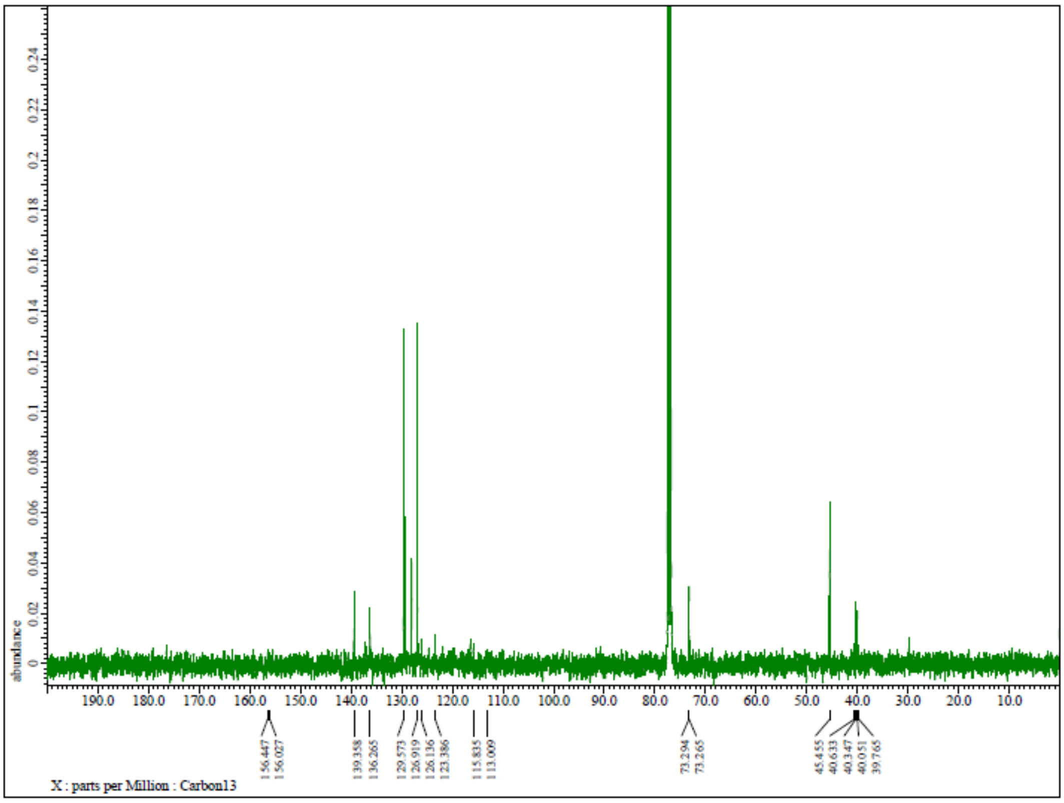
abundance
X : parts per Million : Carbon13
156.447 156.027
139.358 136.265
129.573 126.919 126.136 123.386
115.835 113.009
73.294 73.265
45.455 40.633 40.347 40.051 39.765

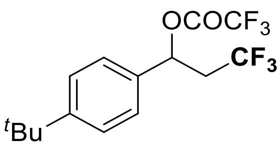
OCOCF3
CF3
tBu

(**2h**)

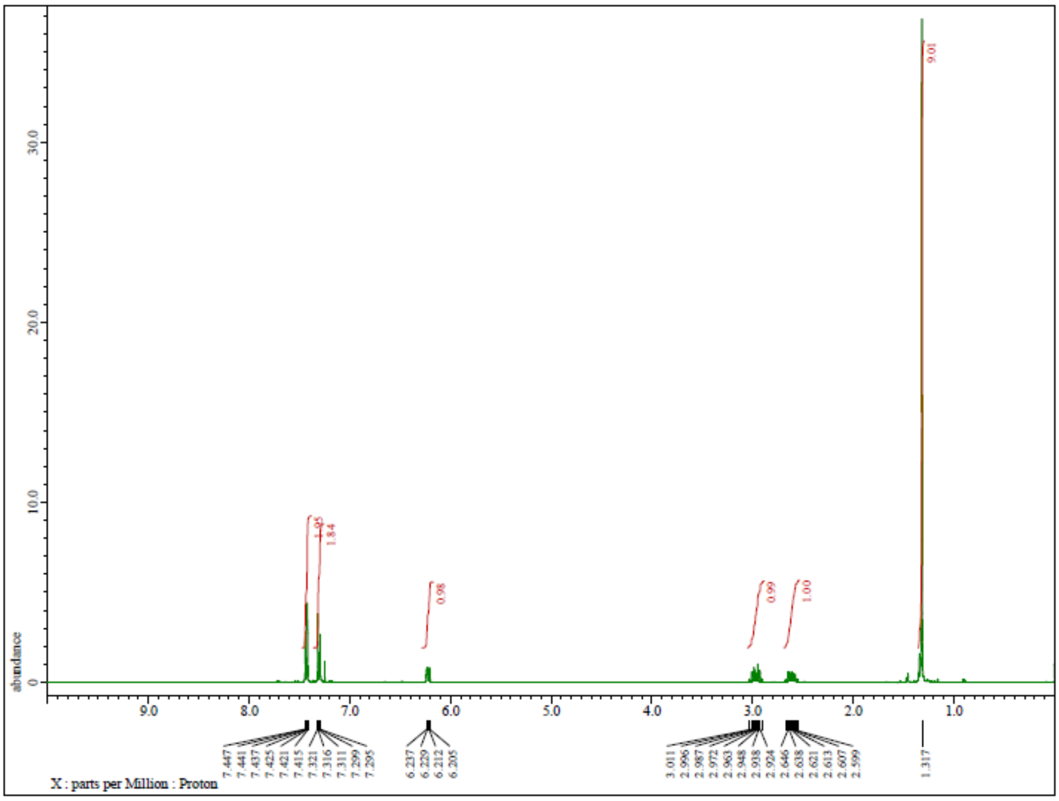
X : parts per Million : Proton
abundance

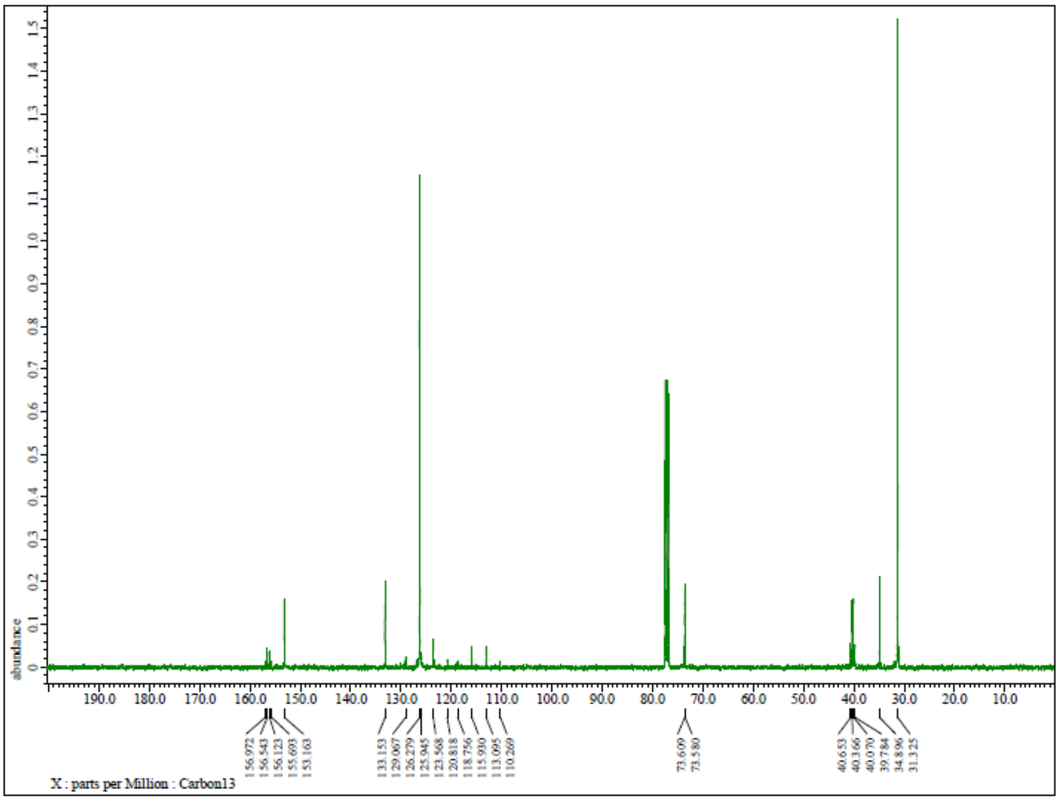
X : parts per Million : Carbon13
abundance

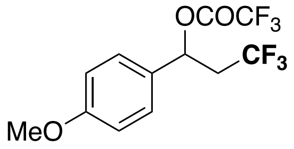
OCOCF3
CF3
MeO

(**2i**)

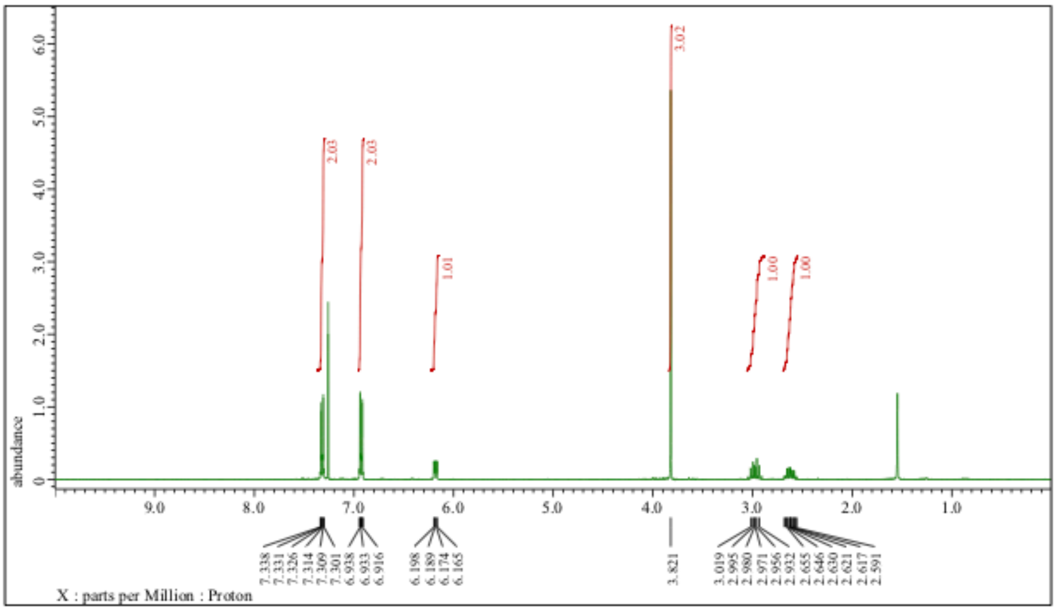
abundance
2.03
2.03
1.01
3.02
1.00
1.00
7.338 7.331 7.326 7.314 7.309 7.301 6.938 6.933 6.916
6.198 6.189 6.174 6.165
3.821
3.019 2.995 2.980 2.971 2.956 2.932 2.655 2.646 2.630 2.621 2.617 2.591
X : parts per Million : Proton

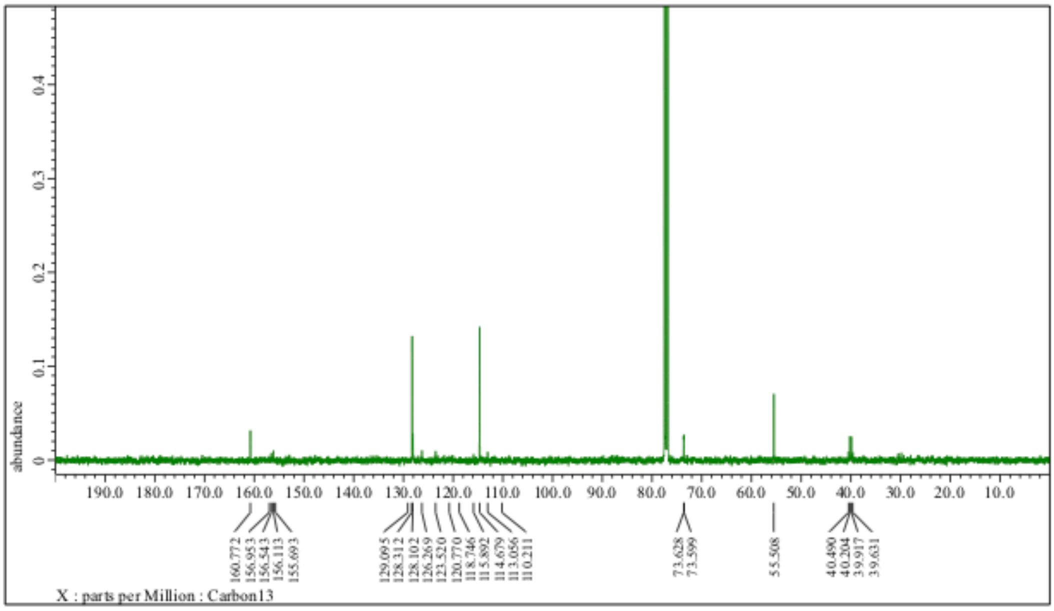
abundance
160.772 156.953 156.543 156.113 155.693
129.095 128.312 128.102 126.269 123.520 120.770 118.746 115.892 114.679 113.056 110.211
73.628 73.599
55.508
40.490 40.204 39.917 39.631
X : parts per Million : Carbon13

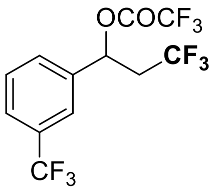
OCOCF3
CF3
CF3

(**2j**)

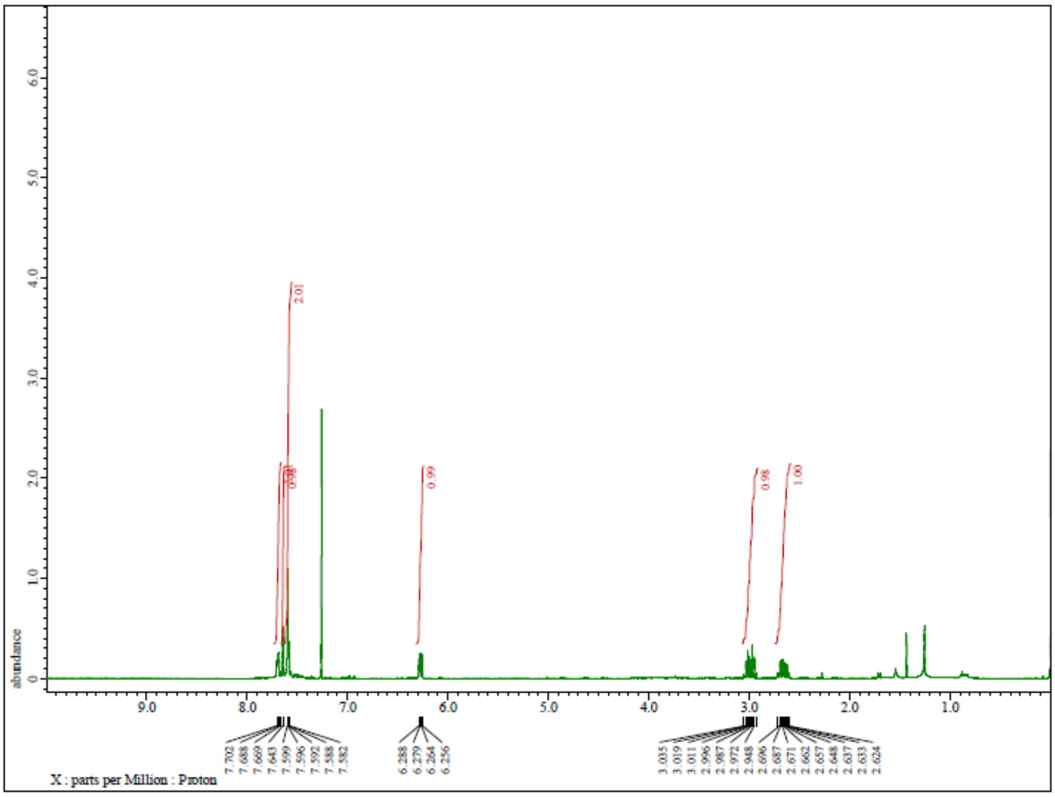
abundance
X : parts per Million : Proton
7.702
7.688
7.669
7.643
7.599
7.596
7.592
7.588
7.582
6.288
6.279
6.264
6.256
3.035
3.019
3.011
2.996
2.987
2.972
2.948
2.696
2.687
2.671
2.662
2.657
2.648
2.637
2.633
2.624
2.01
0.99
0.98
1.00

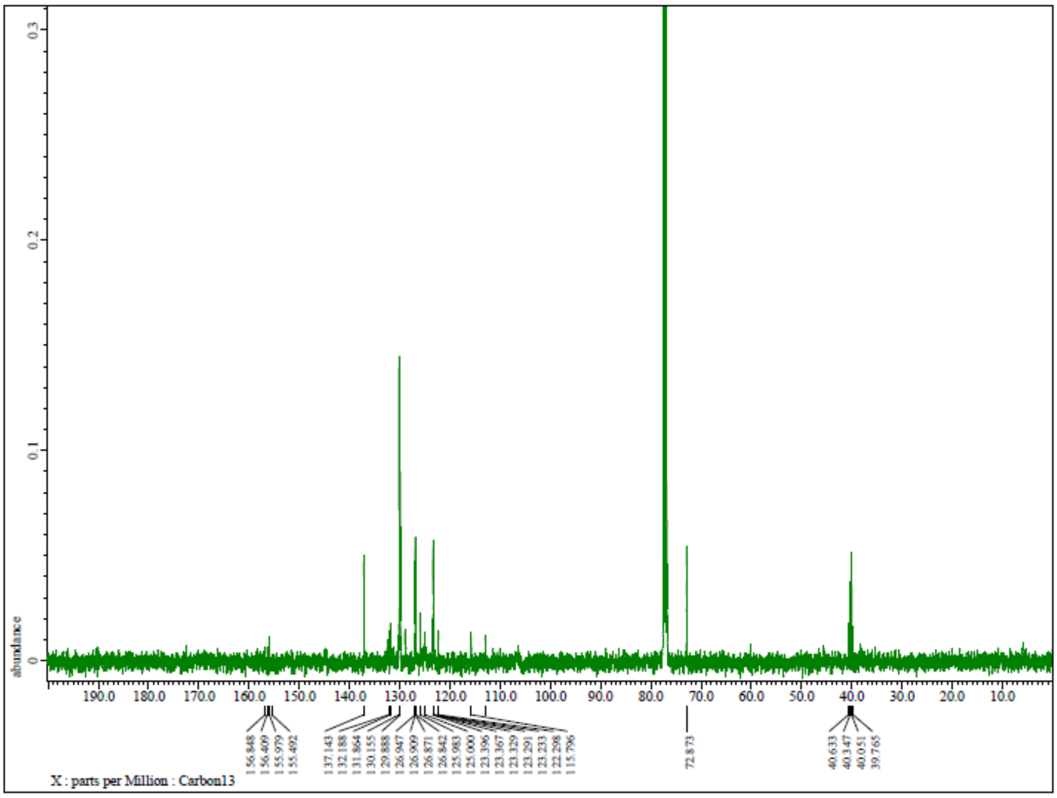
abundance
X : parts per Million : Carbon13
156.848
156.409
155.979
155.492
137.143
132.188
131.864
130.155
129.888
126.947
126.909
126.871
126.842
125.983
125.000
123.396
123.367
123.329
123.291
123.233
122.298
115.796
72.873
40.633
40.347
40.051
39.765

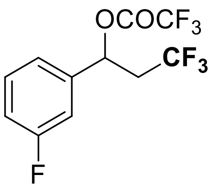

(**2k**)

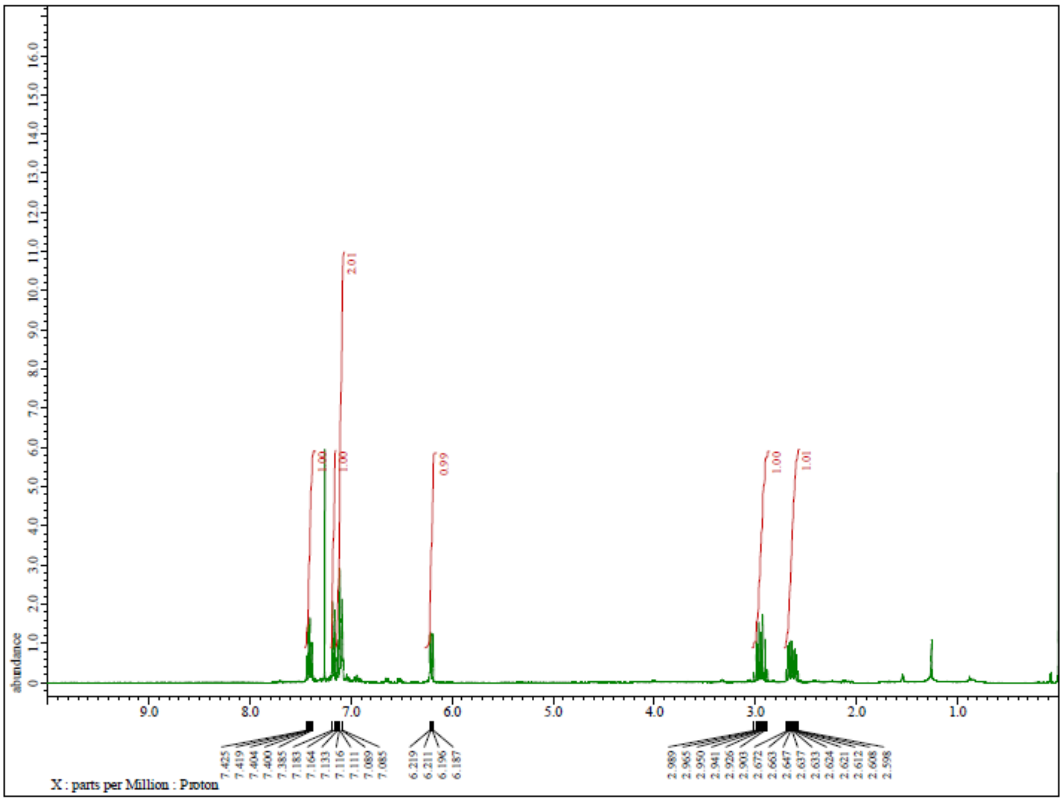

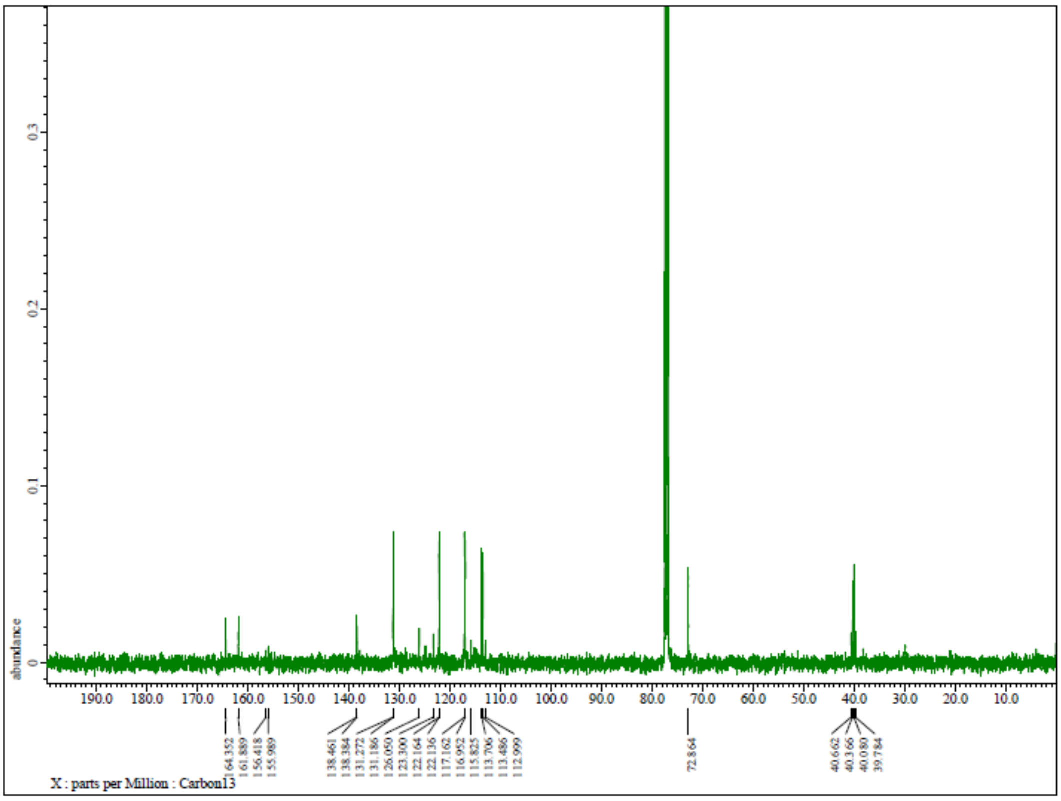

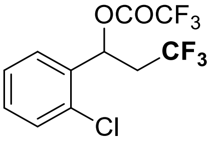
OCOCF3
CF3
Cl

(**2l**)

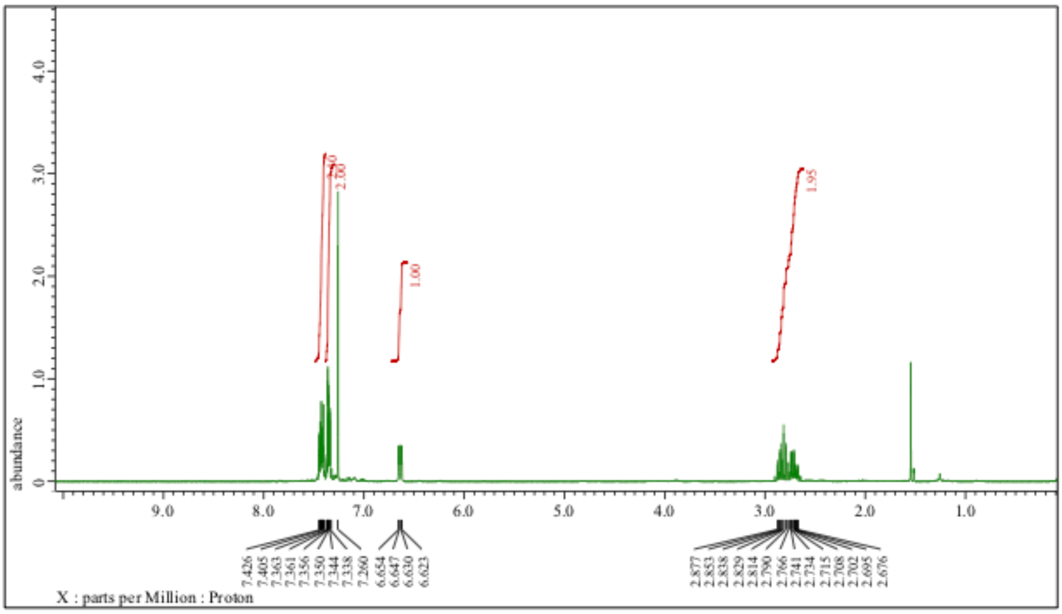
abundance
4.0
3.0
2.0
1.0
0
2.00
1.00
1.95
9.0
8.0
7.0
6.0
5.0
4.0
3.0
2.0
1.0
7.426
7.405
7.363
7.361
7.356
7.350
7.344
7.338
7.260
6.654
6.647
6.630
6.623
2.877
2.853
2.838
2.829
2.814
2.790
2.766
2.741
2.734
2.715
2.708
2.702
2.695
2.676
X : parts per Million : Proton

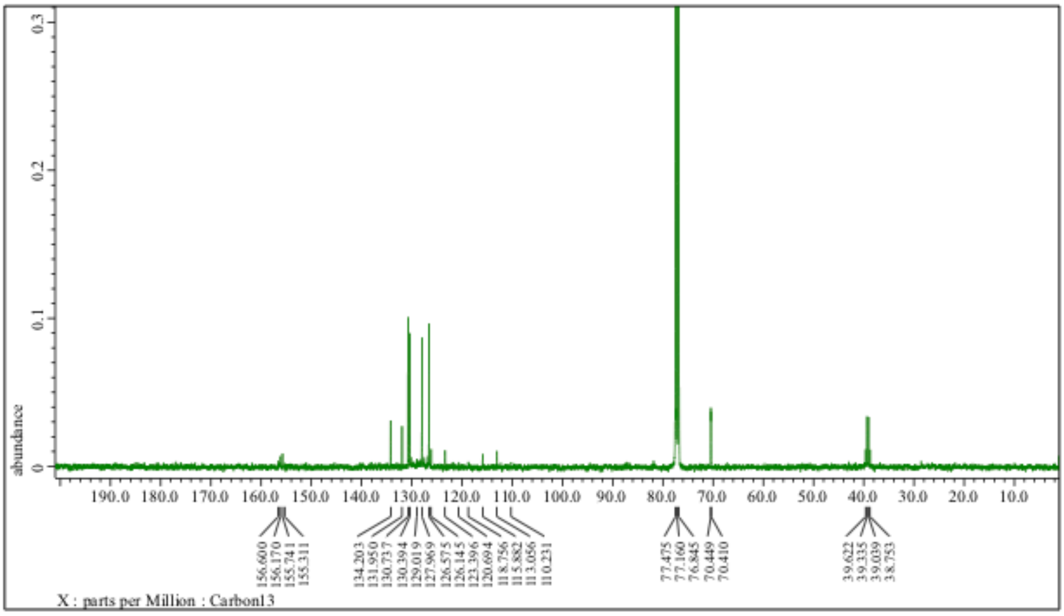
abundance
0.3
0.2
0.1
0
190.0
180.0
170.0
160.0
150.0
140.0
130.0
120.0
110.0
100.0
90.0
80.0
70.0
60.0
50.0
40.0
30.0
20.0
10.0
156.600
156.170
155.741
155.311
134.203
131.950
130.737
130.394
129.019
127.969
126.575
126.145
123.396
120.694
118.756
115.882
113.056
110.231
77.475
77.160
76.845
70.449
70.410
39.622
39.335
39.039
38.753
X : parts per Million : Carbon13

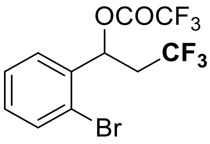
OCOCF3
CF3
Br

(**2m**)

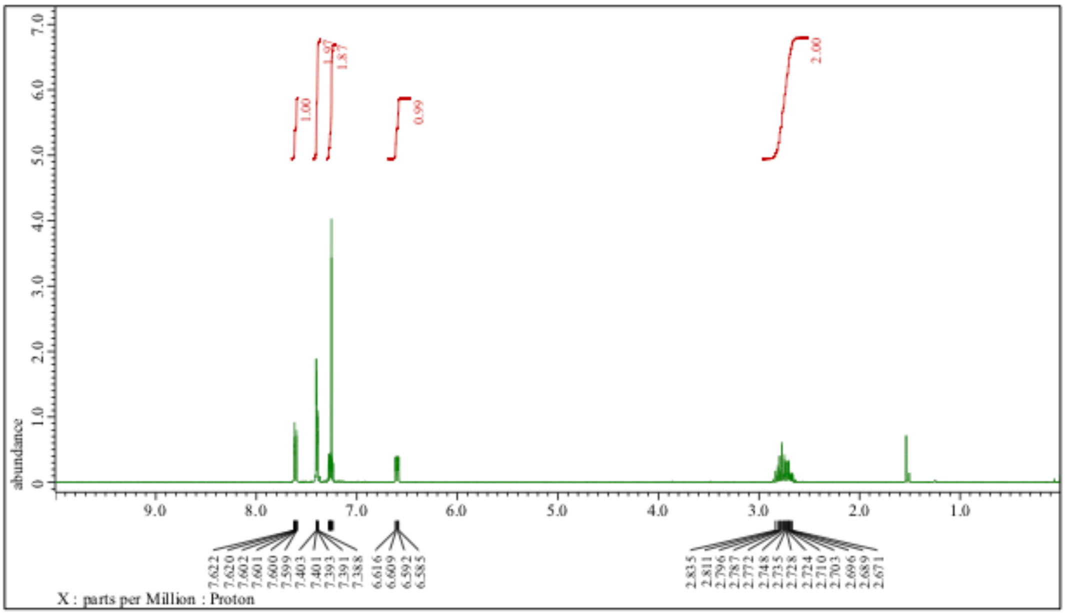
abundance
1.00
1.97
1.87
0.99
2.00
9.0 8.0 7.0 6.0 5.0 4.0 3.0 2.0 1.0
7.622 7.620 7.602 7.601 7.600 7.599 7.403 7.401 7.393 7.391 7.388
6.616 6.609 6.592 6.585
2.835 2.811 2.796 2.787 2.772 2.748 2.735 2.728 2.724 2.710 2.703 2.696 2.689 2.671
X : parts per Million : Proton

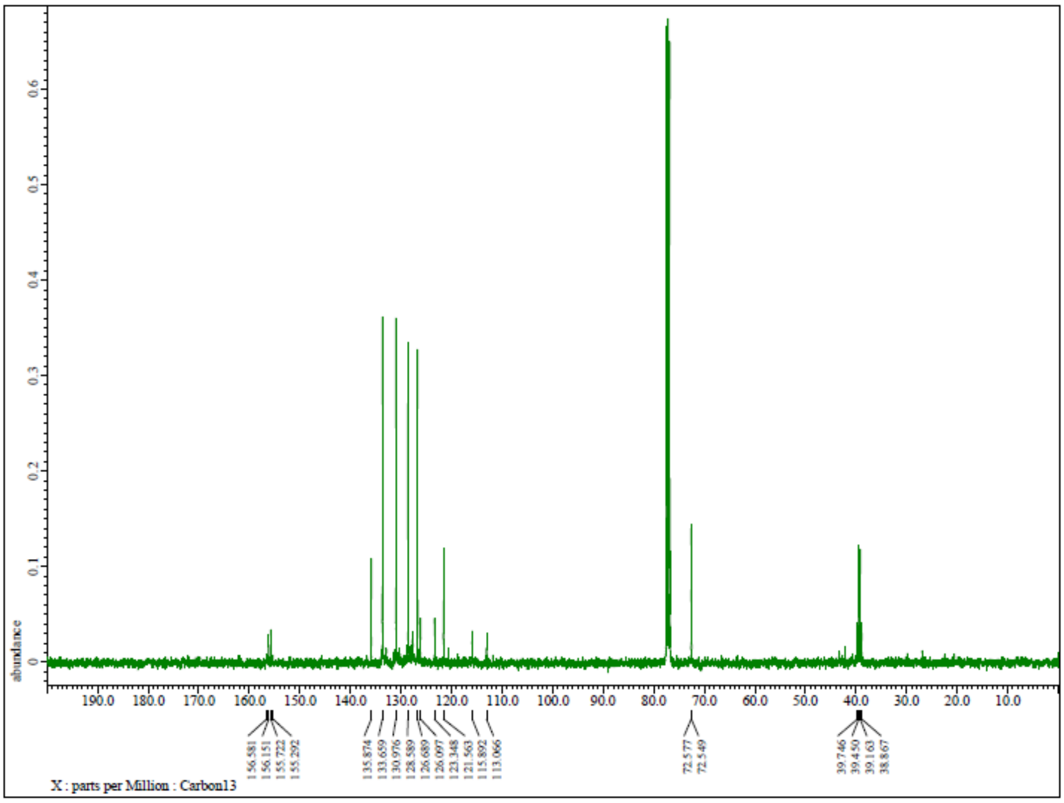
abundance
190.0 180.0 170.0 160.0 150.0 140.0 130.0 120.0 110.0 100.0 90.0 80.0 70.0 60.0 50.0 40.0 30.0 20.0 10.0
156.581 156.151 155.722 155.292
135.874 133.659 130.976 128.589 126.689 126.097 123.348 121.563 115.892 113.066
72.577 72.549
39.746 39.450 39.163 38.867
X : parts per Million : Carbon13

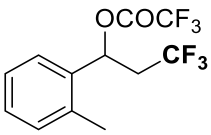
OCOCF3
CF3

(**2n**)

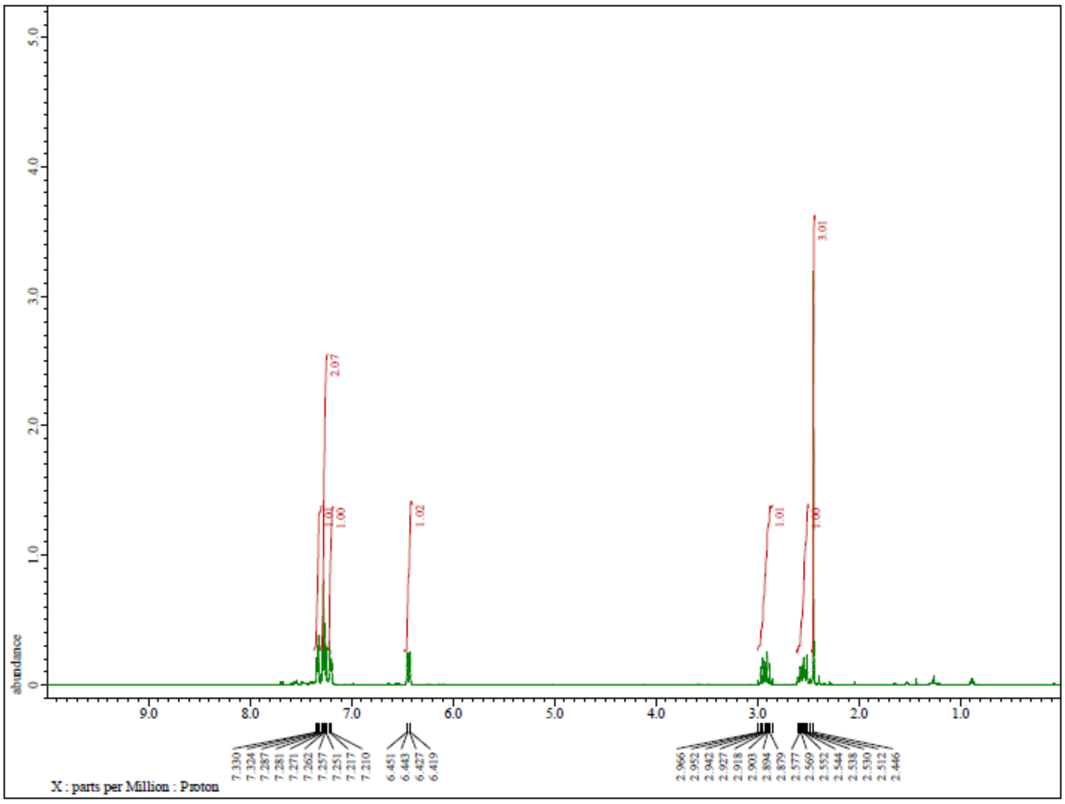
5.0
4.0
3.0
2.0
1.0
0
abundance
2.07
1.01
1.00
1.02
1.01
1.00
3.01
9.0
8.0
7.0
6.0
5.0
4.0
3.0
2.0
1.0
7.330
7.324
7.287
7.281
7.271
7.262
7.257
7.251
7.217
7.210
6.451
6.443
6.427
6.419
2.966
2.952
2.942
2.927
2.918
2.903
2.894
2.879
2.577
2.569
2.552
2.544
2.538
2.530
2.512
2.446
X : parts per Million : Proton

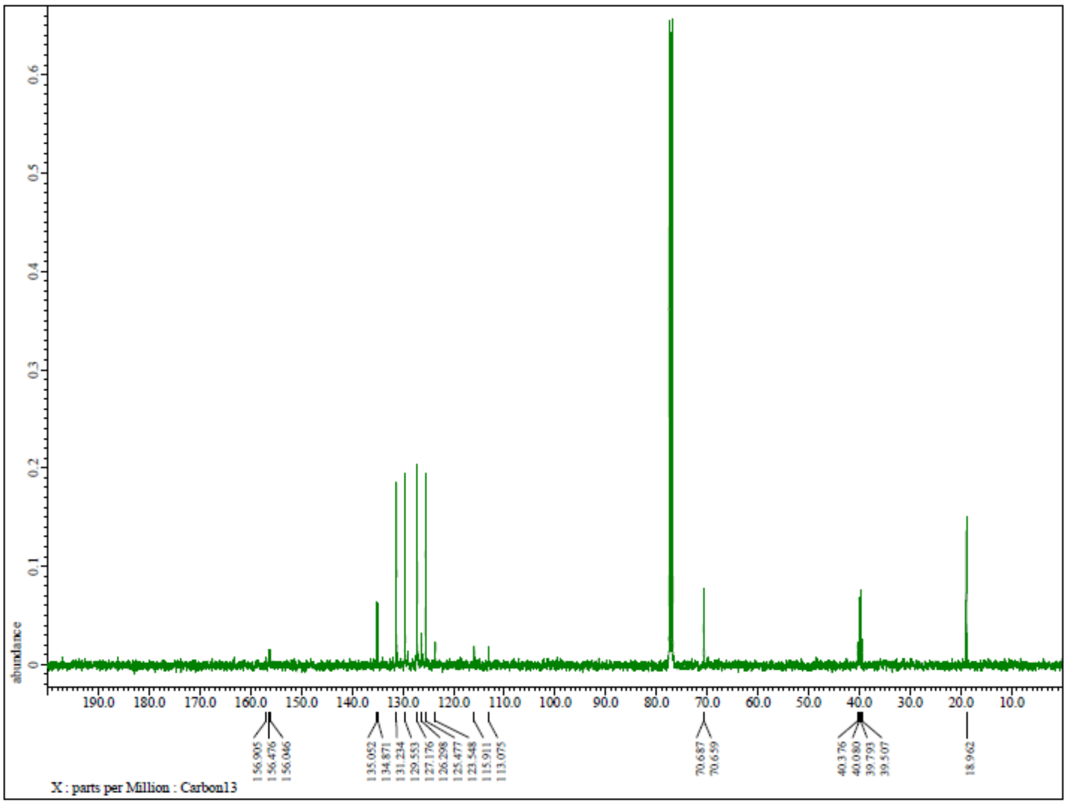
0.6
0.5
0.4
0.3
0.2
0.1
0
abundance
190.0
180.0
170.0
160.0
150.0
140.0
130.0
120.0
110.0
100.0
90.0
80.0
70.0
60.0
50.0
40.0
30.0
20.0
10.0
156.905
156.476
156.046
135.052
134.871
131.234
129.553
127.176
126.298
125.477
123.548
115.911
113.075
70.687
70.659
40.376
40.080
39.793
39.507
18.962
X : parts per Million : Carbon13

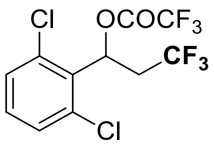
Cl
OCOCF3
CF3
Cl

(**2o**)

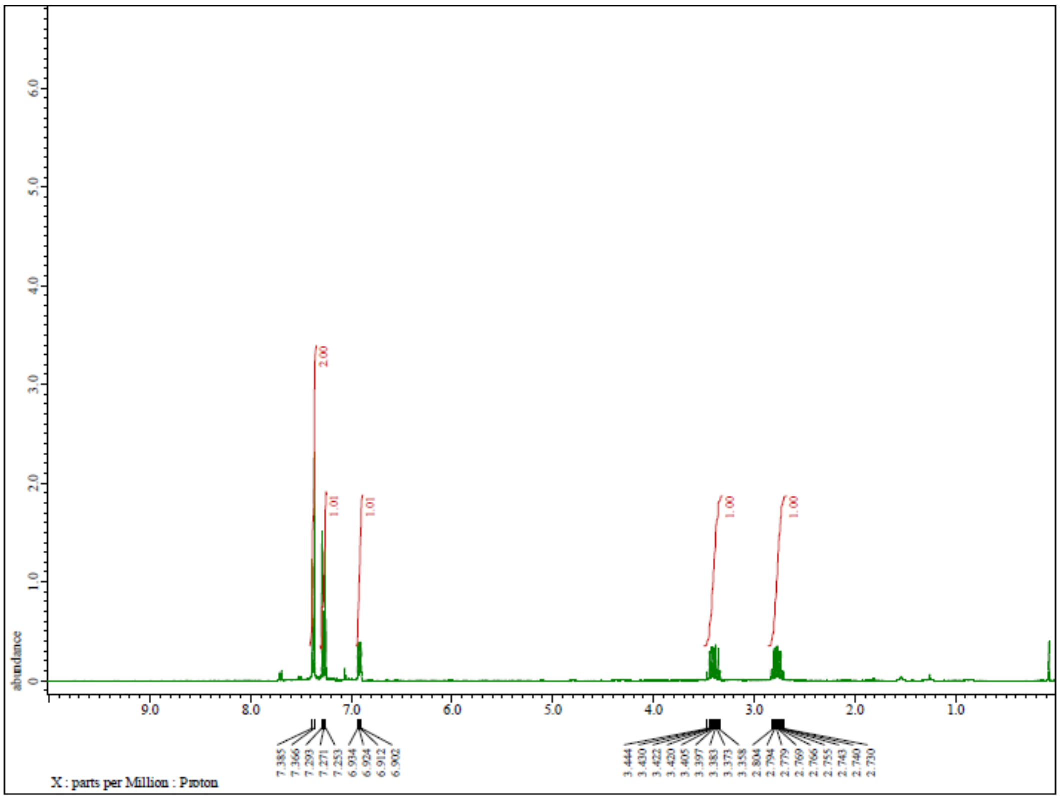
abundance
X : parts per Million : Proton
7.385
7.366
7.293
7.271
7.253
6.934
6.924
6.912
6.902
3.444
3.430
3.422
3.420
3.405
3.397
3.383
3.373
3.358
2.804
2.794
2.779
2.769
2.766
2.755
2.743
2.740
2.730
2.00
1.01
1.01
1.00
1.00

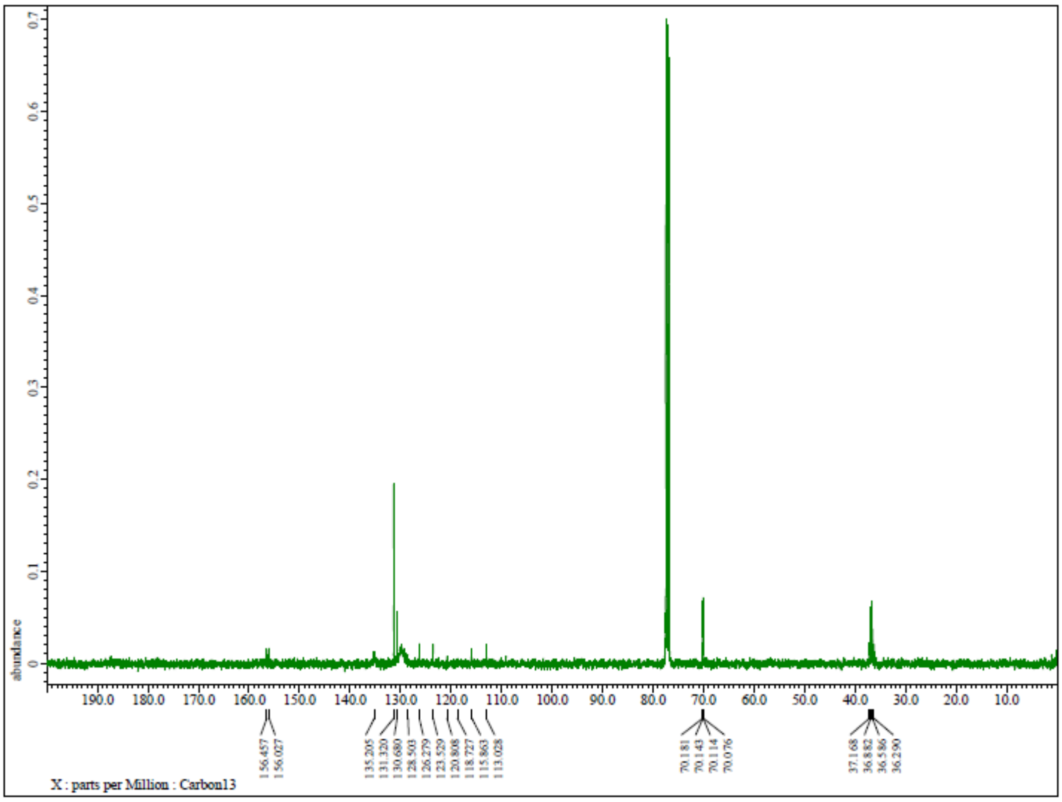
abundance
X : parts per Million : Carbon13
156.457
156.027
135.205
131.320
130.680
128.503
126.279
123.529
120.808
118.727
115.863
113.028
70.181
70.143
70.114
70.076
37.168
36.882
36.586
36.290

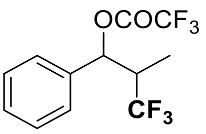

(2p)

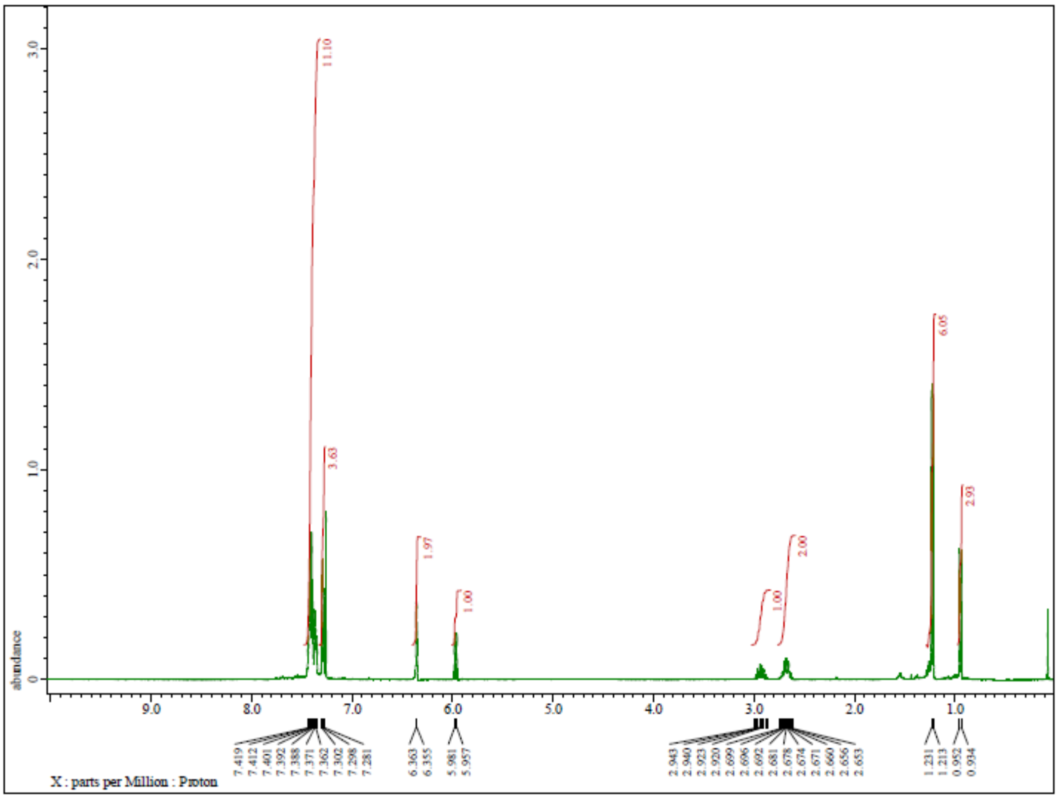

abundance
X : parts per Million : Proton

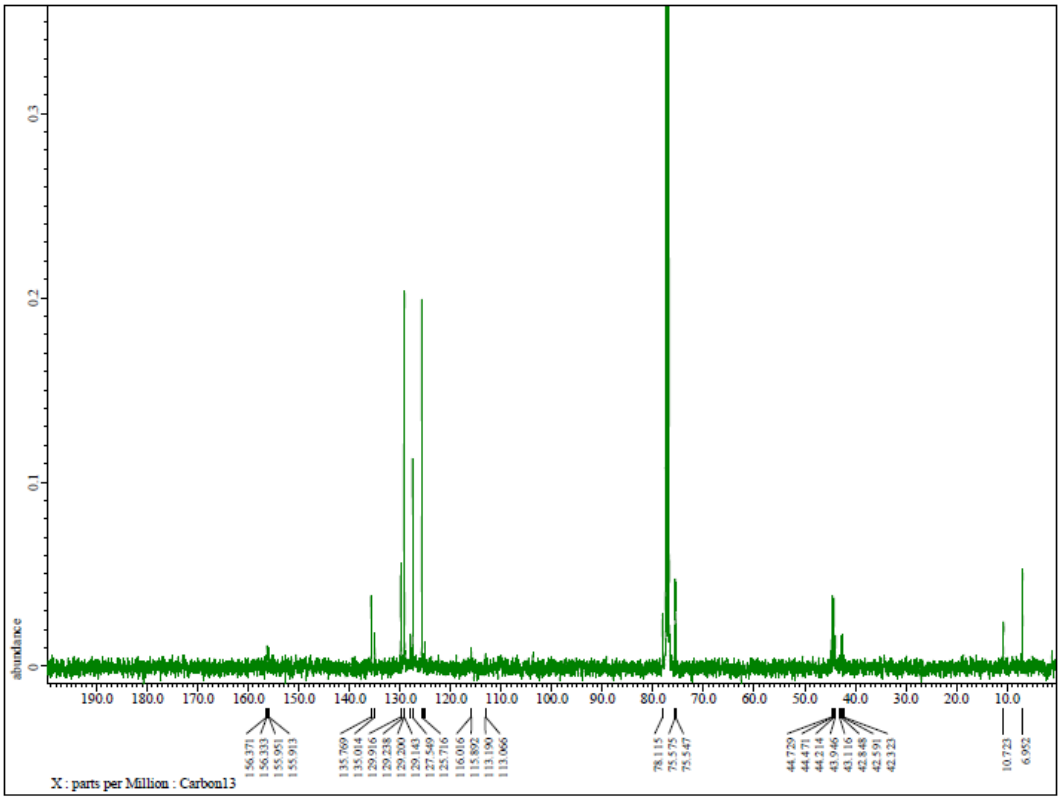

abundance
X : parts per Million : Carbon13

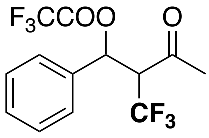
F3CCOO
O
CF3

(**2q**)

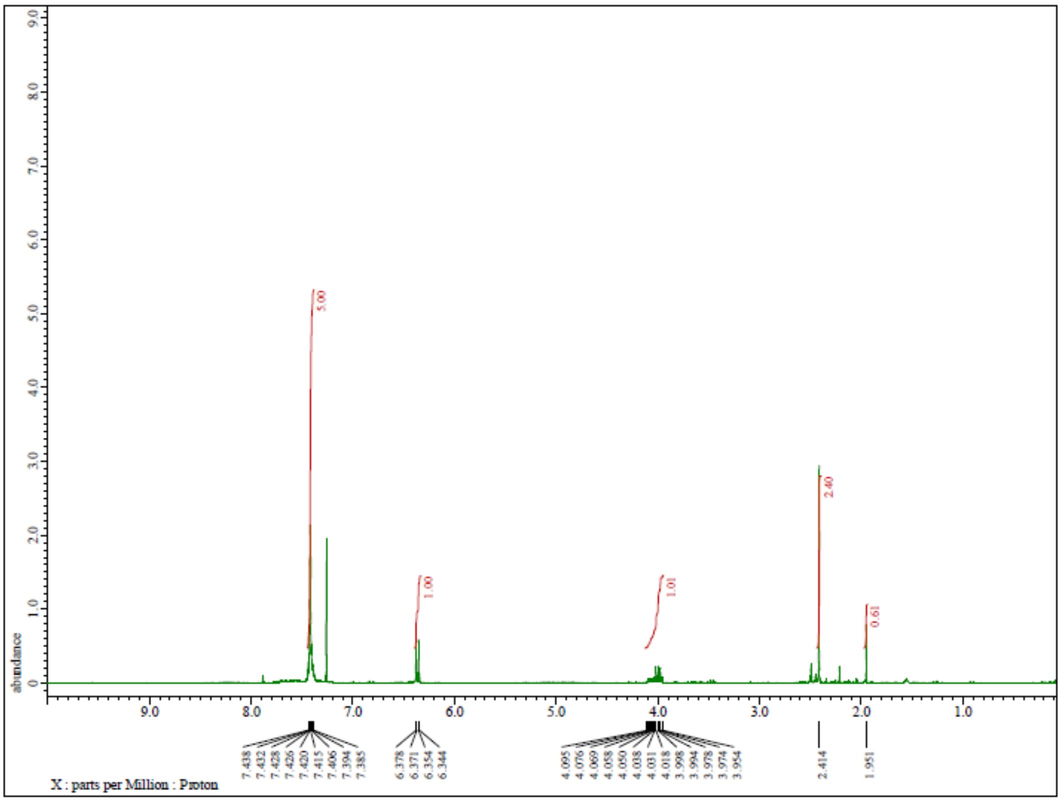
abundance
X : parts per Million : Proton

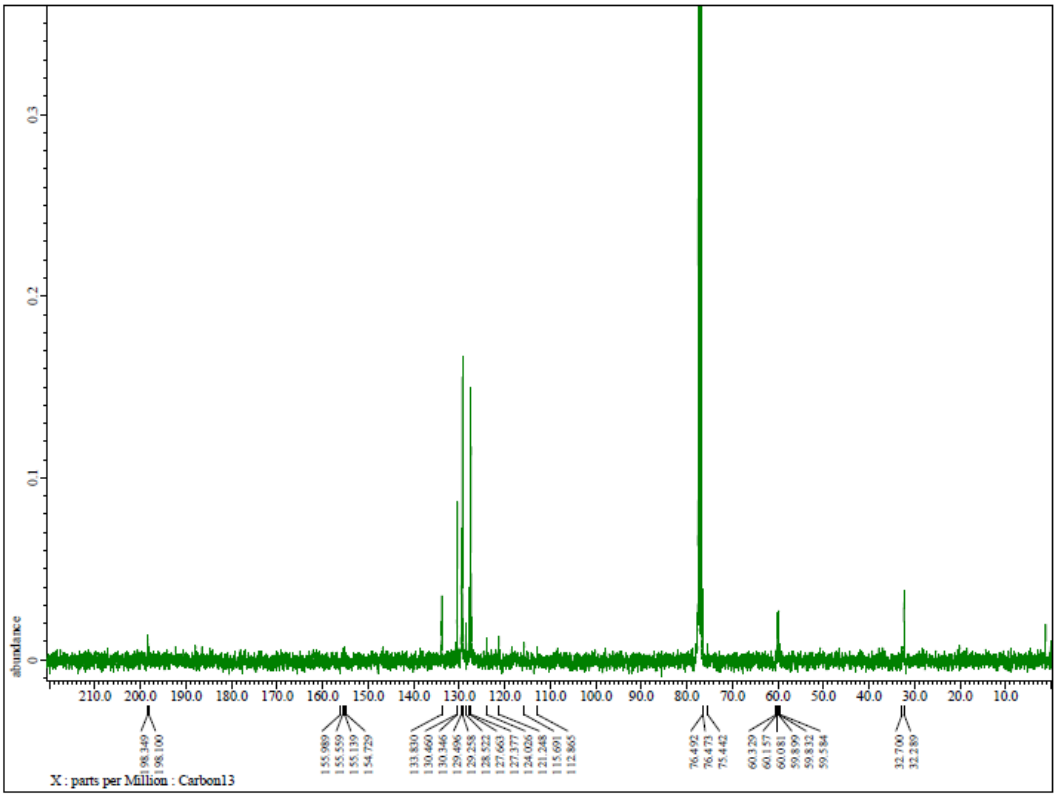
abundance
X : parts per Million : Carbon13

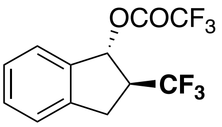

(**2r**, *anti*-isomer)

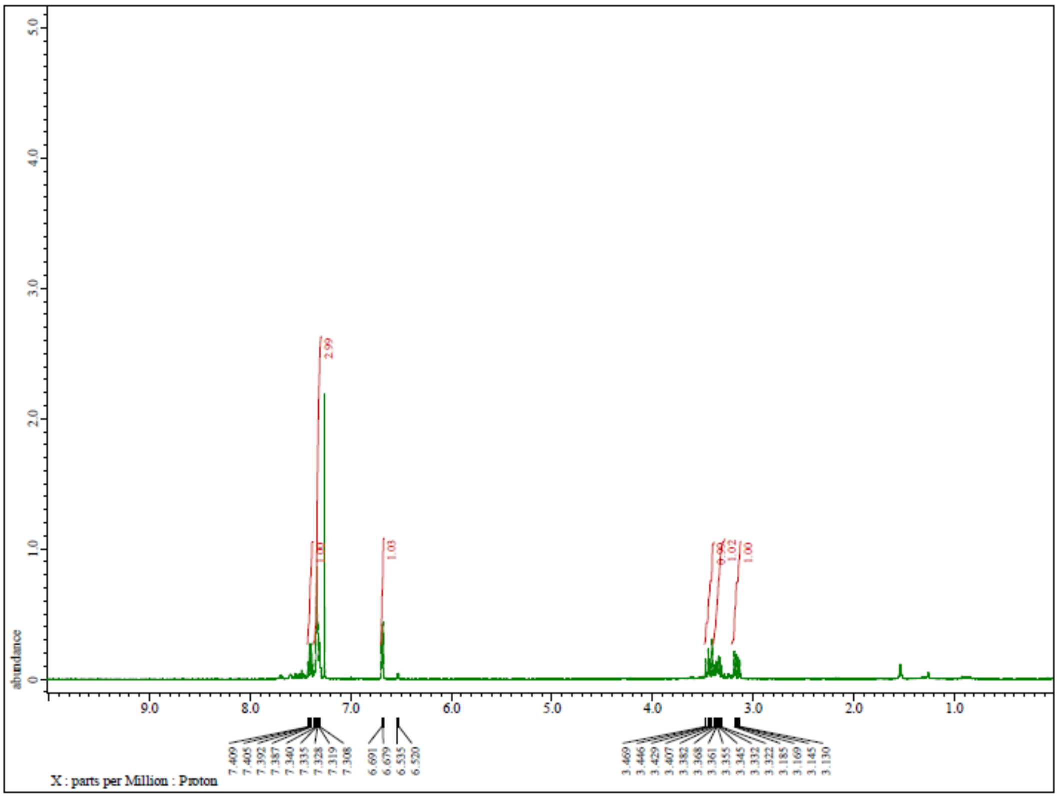

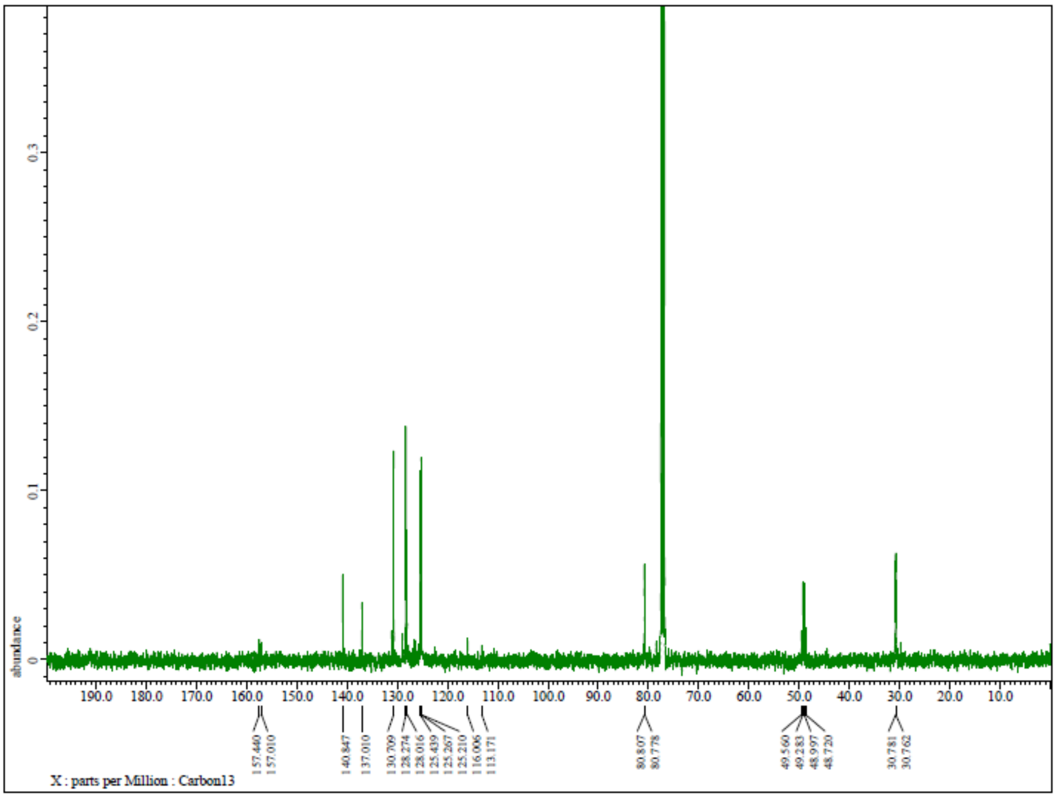

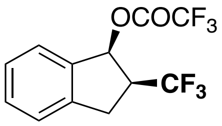

(**2r**, *syn*-isomer)

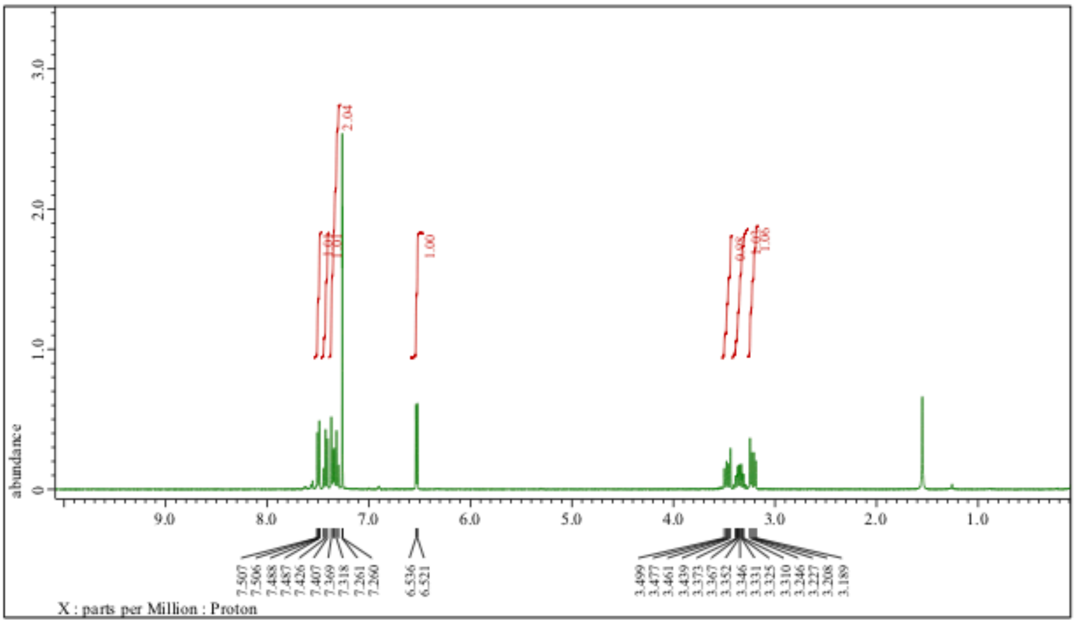

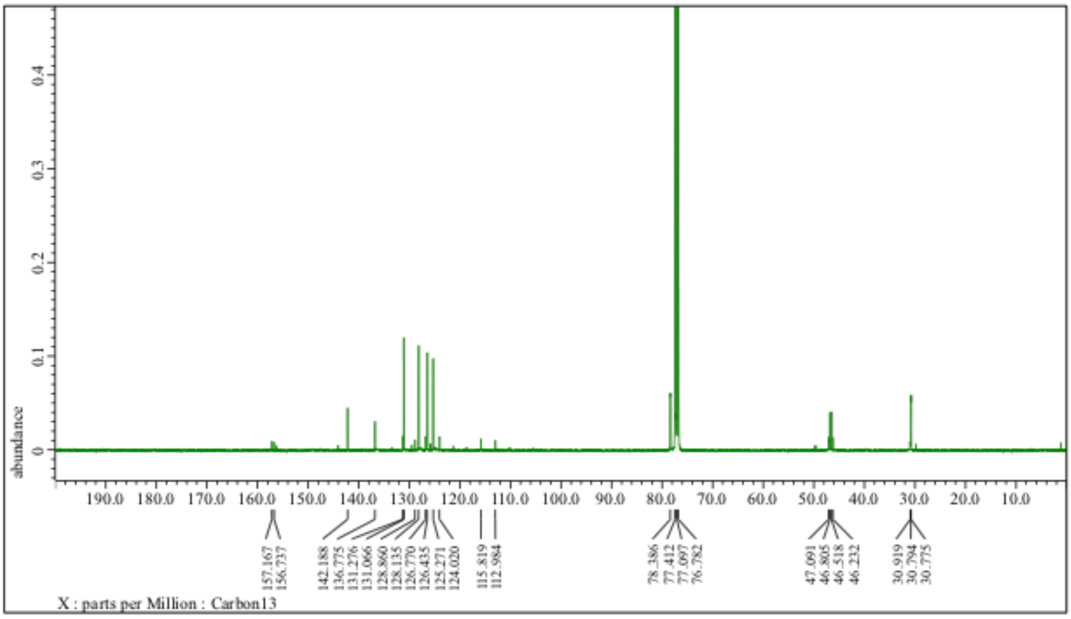

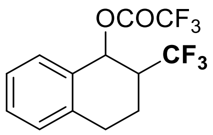

(**2s**, diastereomixture)

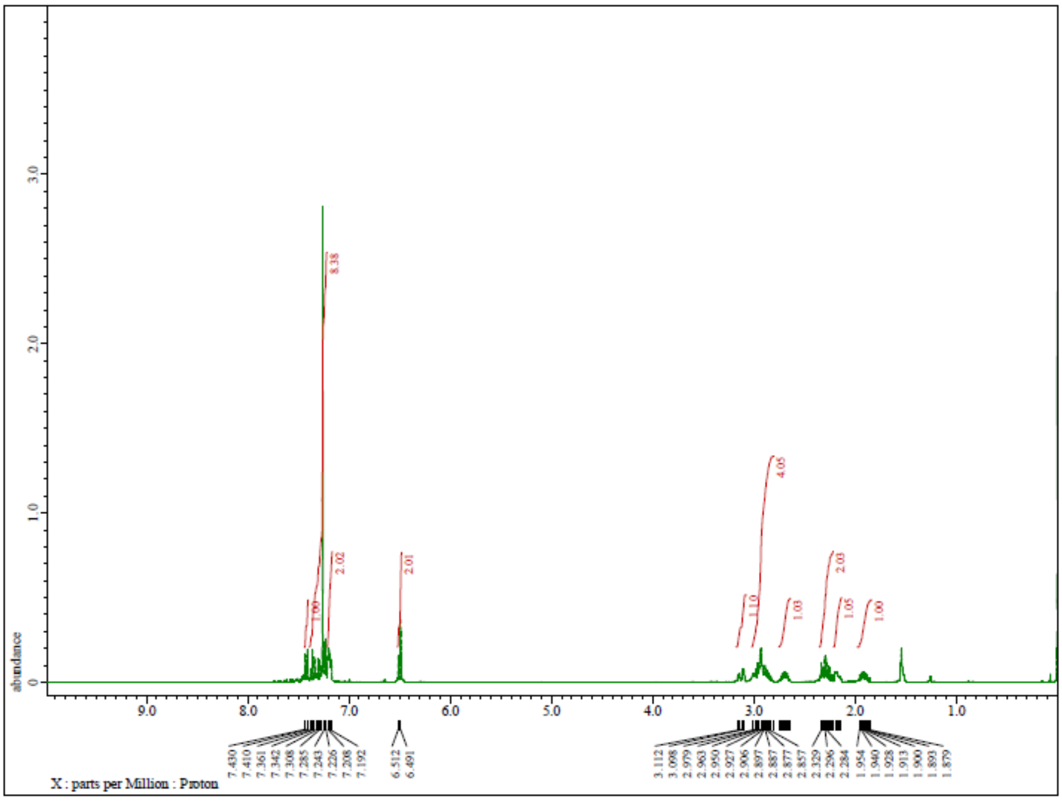

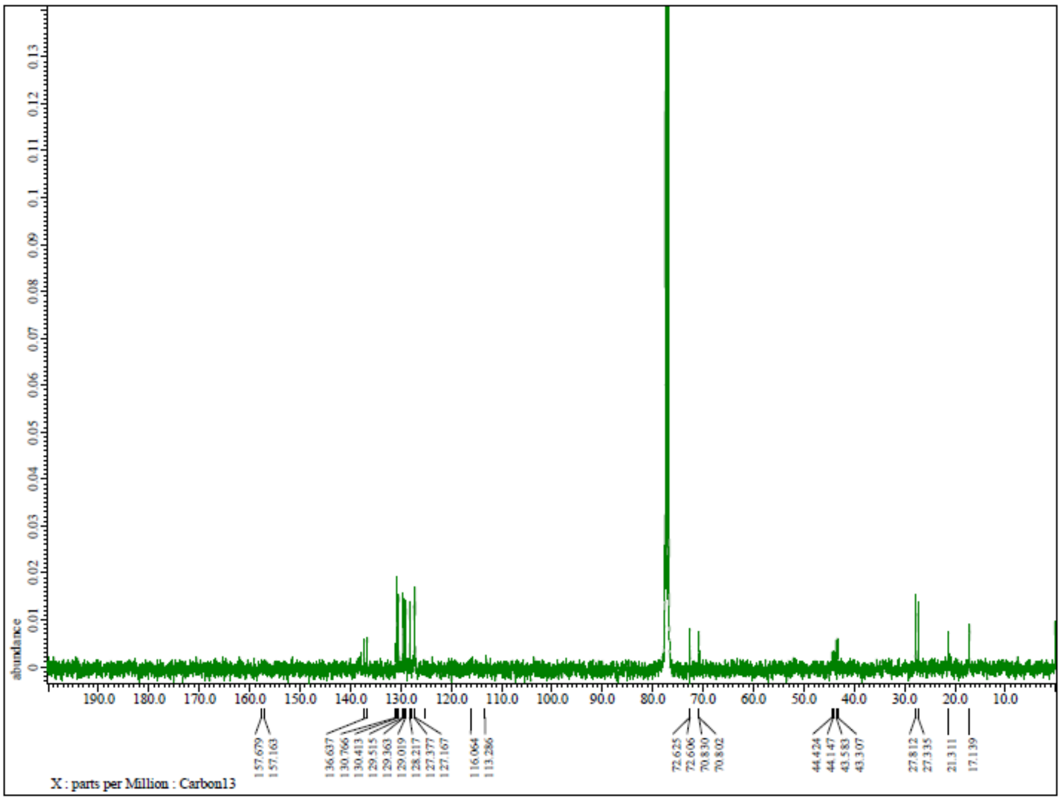

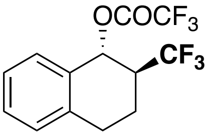

(**2s**, *anti*-isomer)

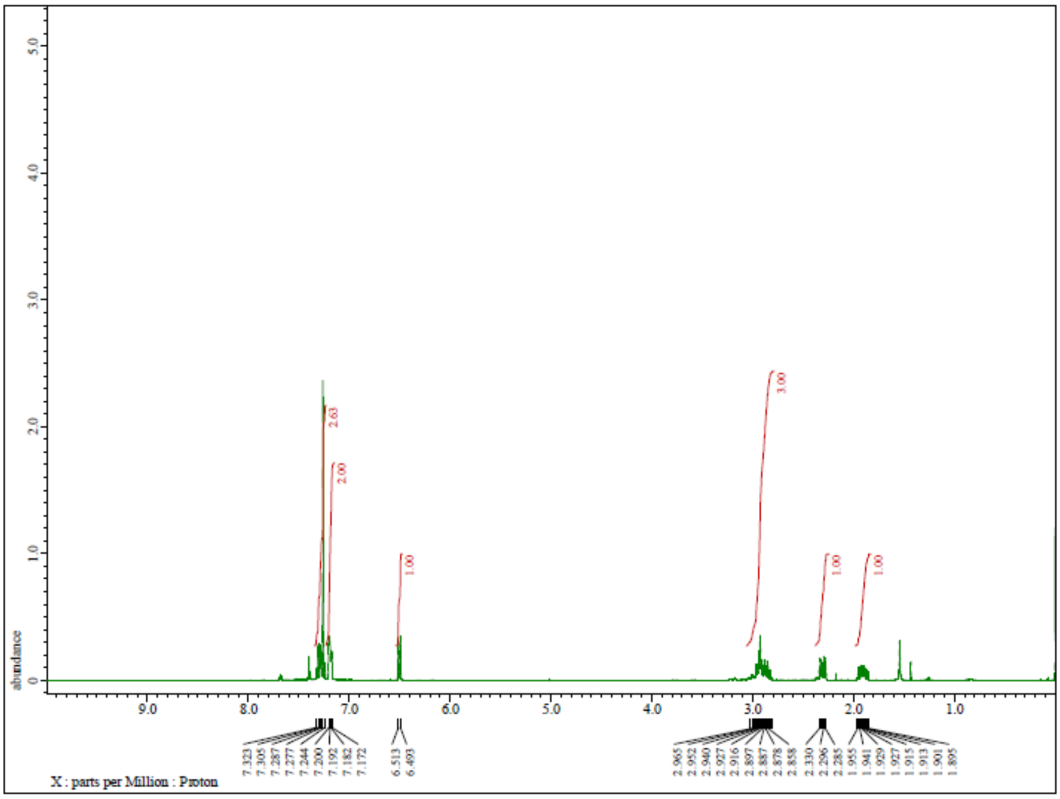

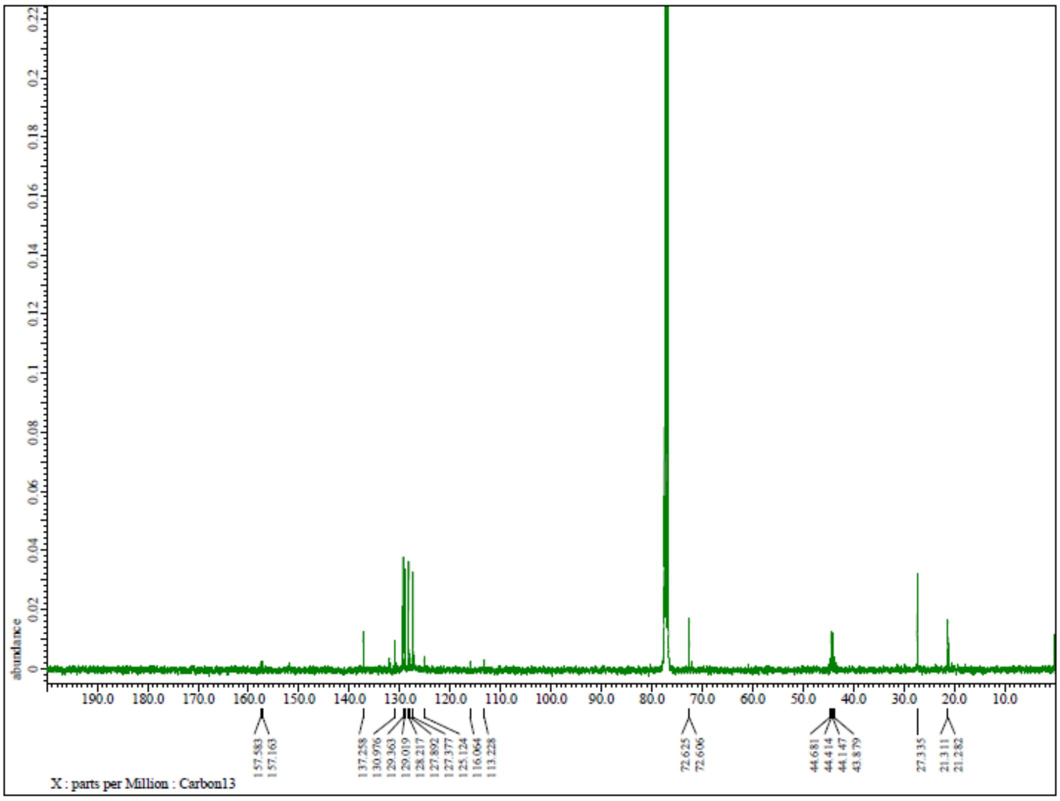

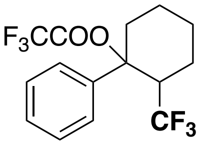
F3CCOO
CF3

(**2t**)

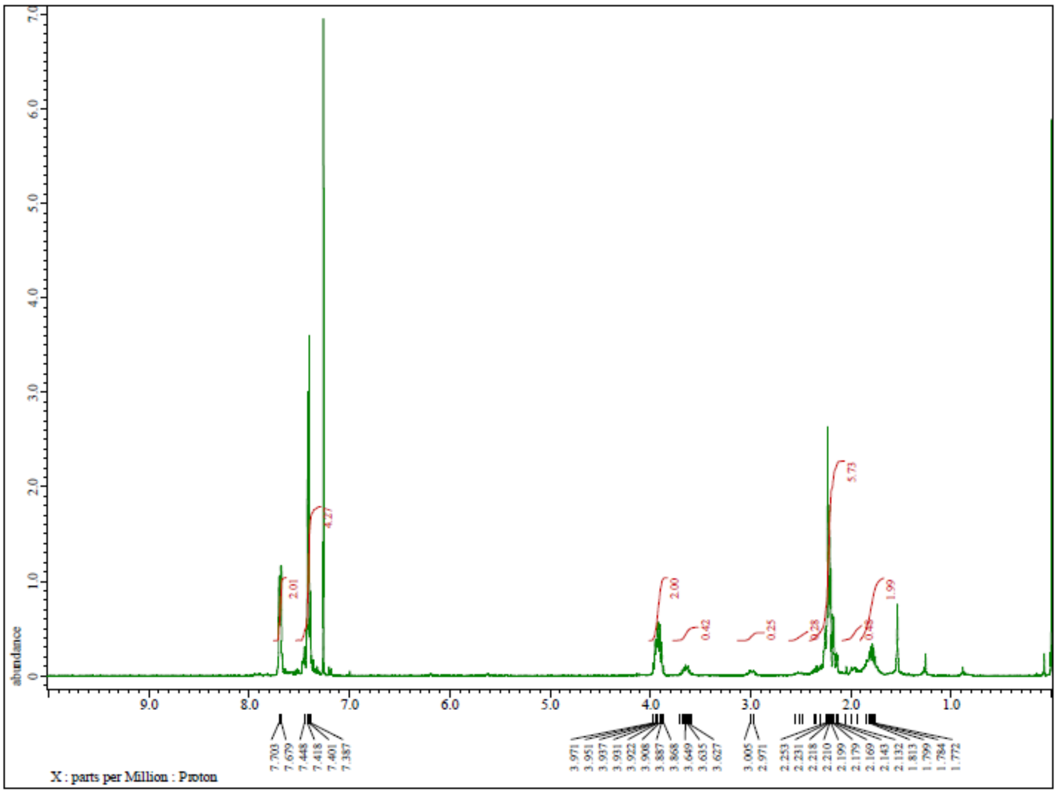
abundance
X : parts per Million : Proton
2.01
4.27
2.00
0.42
0.25
0.28
5.73
1.99
7.703
7.679
7.448
7.418
7.401
7.387
3.971
3.951
3.937
3.931
3.922
3.908
3.887
3.868
3.649
3.635
3.627
3.005
2.971
2.253
2.231
2.218
2.210
2.199
2.179
2.169
2.143
2.132
1.813
1.799
1.784
1.772

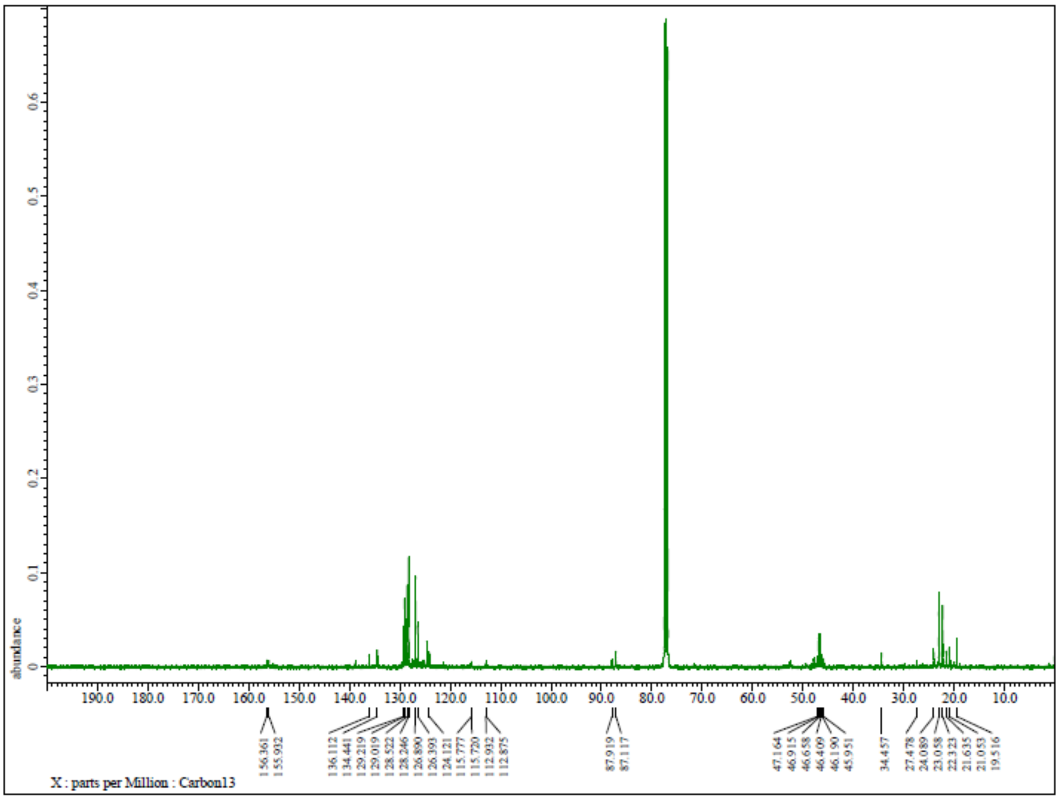
abundance
X : parts per Million : Carbon13
156.361
155.932
136.112
134.441
129.219
129.019
128.522
128.246
126.890
126.393
124.121
115.777
115.720
112.932
112.875
87.919
87.117
47.164
46.915
46.658
46.409
46.190
45.951
34.457
27.478
24.089
23.058
22.323
21.635
21.053
19.516

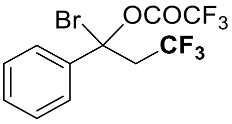
Br
OCOCF3
CF3

(**2u**)

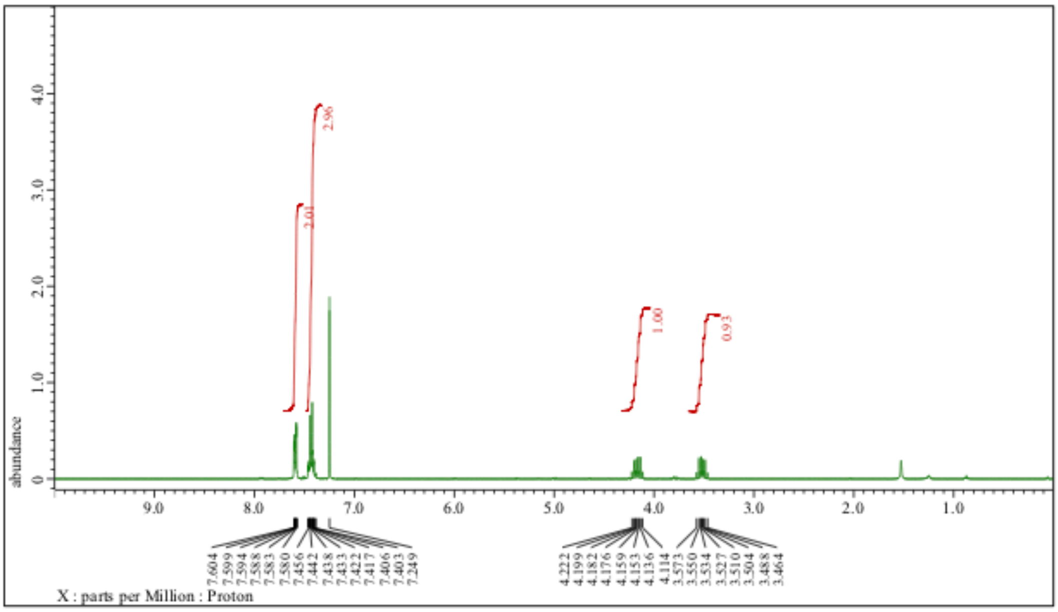
abundance
2.01
2.96
1.00
0.93
7.604
7.599
7.594
7.588
7.583
7.580
7.456
7.442
7.438
7.433
7.422
7.417
7.406
7.403
7.249
4.222
4.199
4.182
4.176
4.159
4.153
4.136
4.114
3.573
3.550
3.534
3.527
3.510
3.504
3.488
3.464
X : parts per Million : Proton

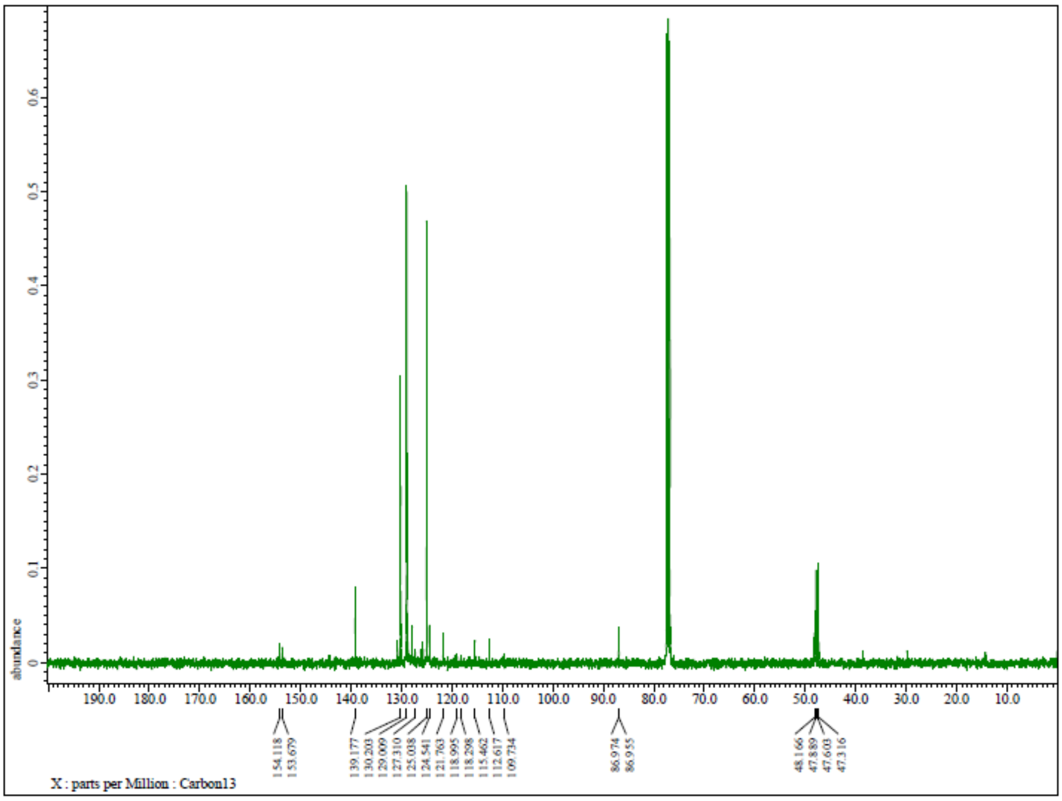
abundance
154.118
153.679
139.177
130.203
129.009
127.310
125.038
124.541
121.763
118.995
118.298
115.462
112.617
109.734
86.974
86.955
48.166
47.889
47.603
47.316
X : parts per Million : Carbon13

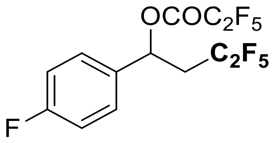

(**2b'**)

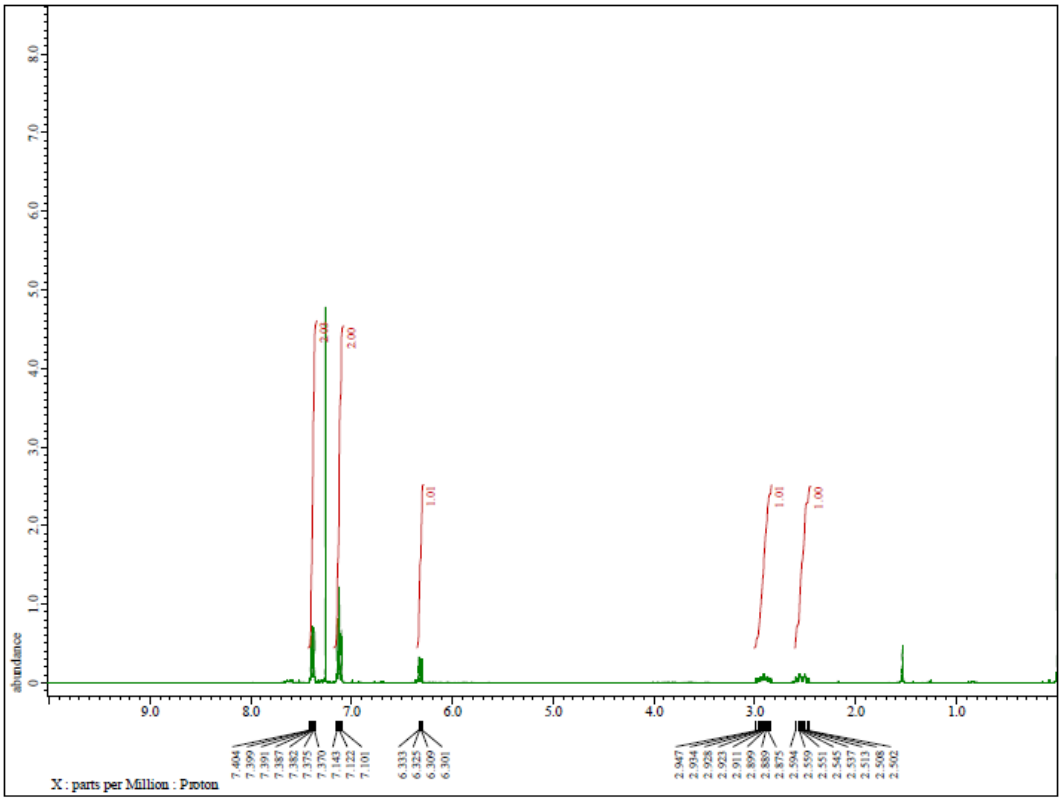

X : parts per Million : Proton
abundance

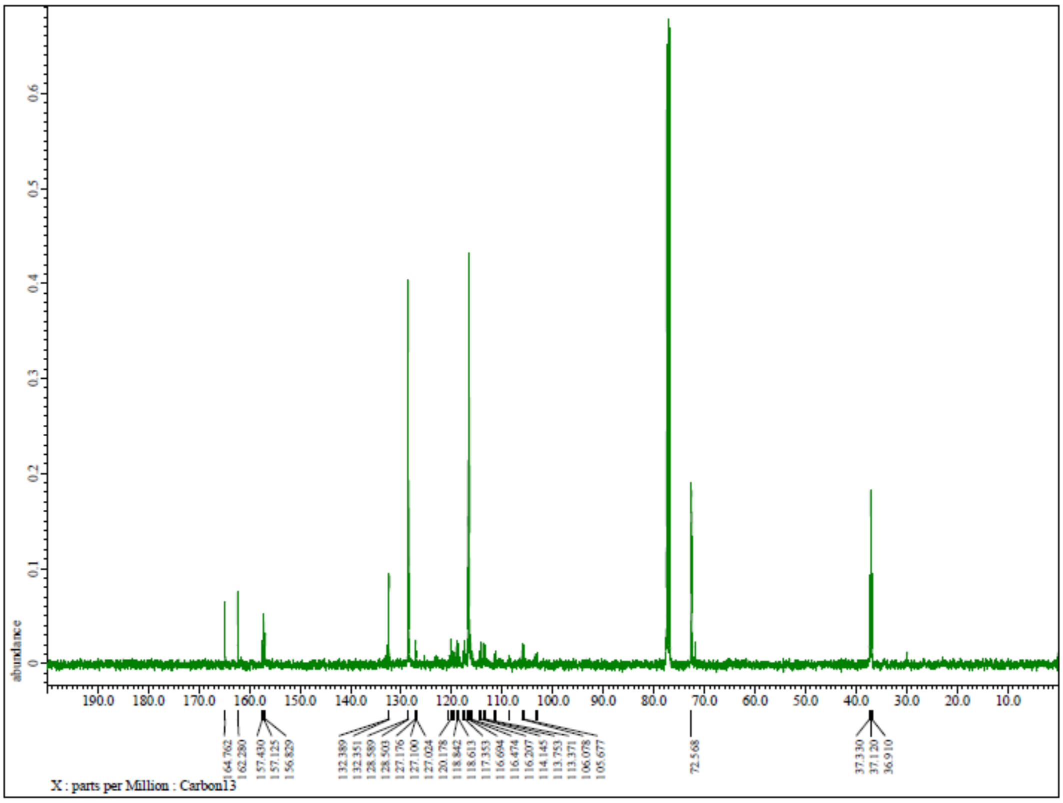

X : parts per Million : Carbon13
abundance

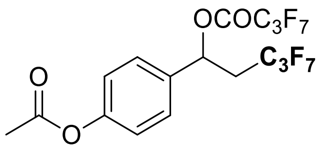
OCOC3F7
C3F7
O
O

(**2d''**)

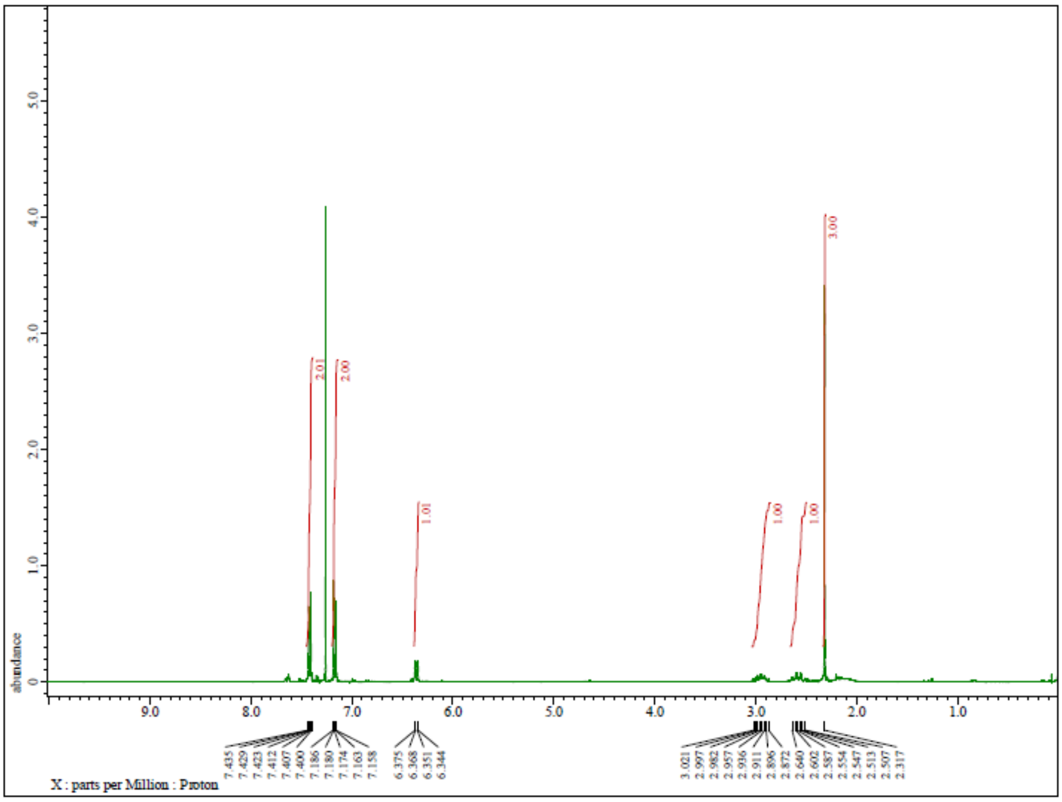
abundance
X : parts per Million : Proton
9.0 8.0 7.0 6.0 5.0 4.0 3.0 2.0 1.0
2.01 2.00 1.01 1.00 1.00 3.00
7.435 7.429 7.423 7.412 7.407 7.400 7.186 7.180 7.174 7.163 7.158
6.375 6.368 6.351 6.344
3.021 2.997 2.982 2.957 2.936 2.911 2.896 2.872 2.640 2.602 2.587 2.554 2.547 2.513 2.507 2.317

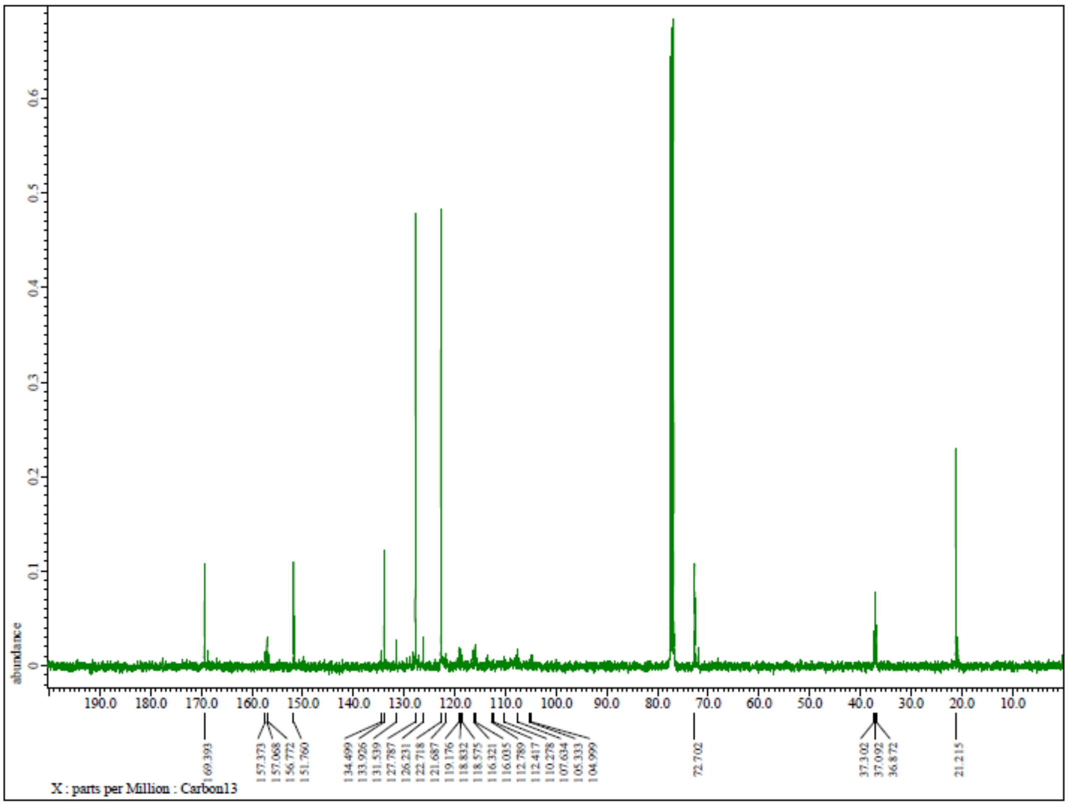
abundance
X : parts per Million : Carbon13
190.0 180.0 170.0 160.0 150.0 140.0 130.0 120.0 110.0 100.0 90.0 80.0 70.0 60.0 50.0 40.0 30.0 20.0 10.0
169.393
157.373 157.068 156.772 151.760
134.499 133.926 131.539 127.787 126.231 122.718 121.687 119.176 118.832 118.575 116.321 116.035 112.789 112.417 110.278 107.634 105.333 104.999
72.702
37.302 37.092 36.872
21.215

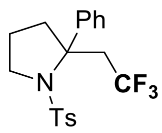
Ph
N
Ts
CF3

(**4a**)

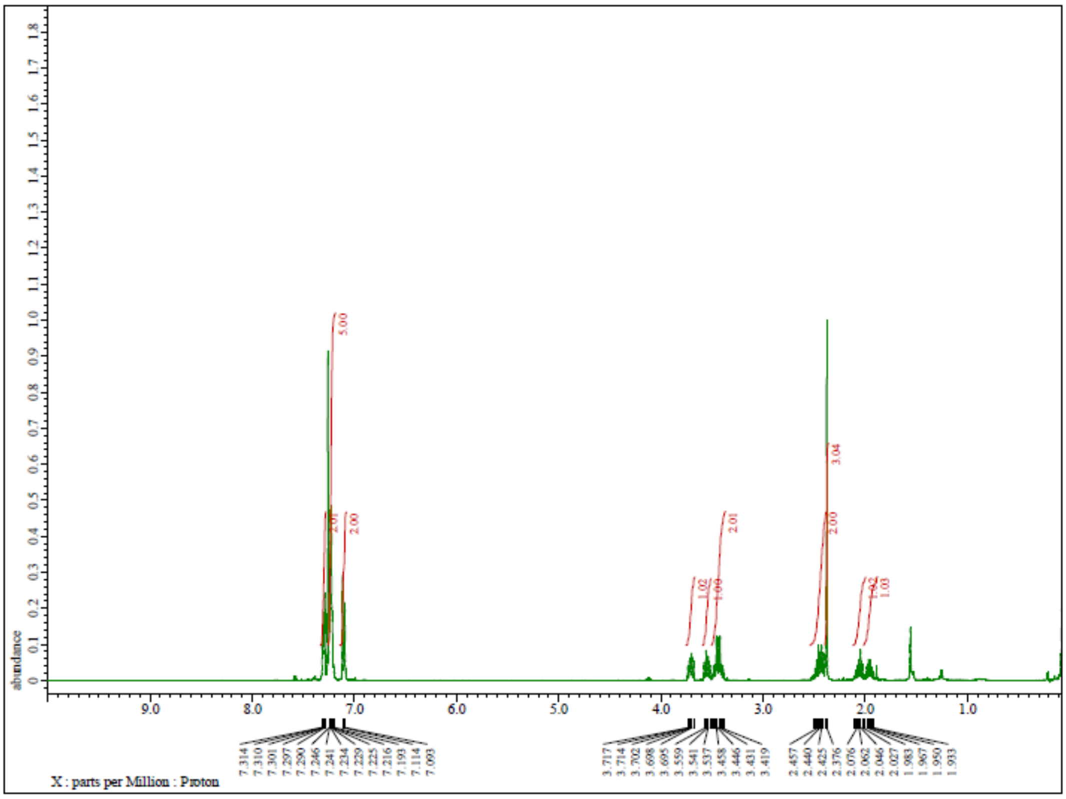
abundance
X : parts per Million : Proton
9.0
8.0
7.0
6.0
5.0
4.0
3.0
2.0
1.0
5.00
2.01
2.00
1.02
1.09
2.01
3.04
2.00
1.02
1.03
7.314
7.310
7.301
7.297
7.290
7.246
7.241
7.234
7.229
7.225
7.216
7.193
7.114
7.093
3.717
3.714
3.702
3.698
3.695
3.559
3.541
3.537
3.458
3.446
3.431
3.419
2.457
2.440
2.425
2.376
2.076
2.062
2.046
2.027
1.983
1.967
1.950
1.933

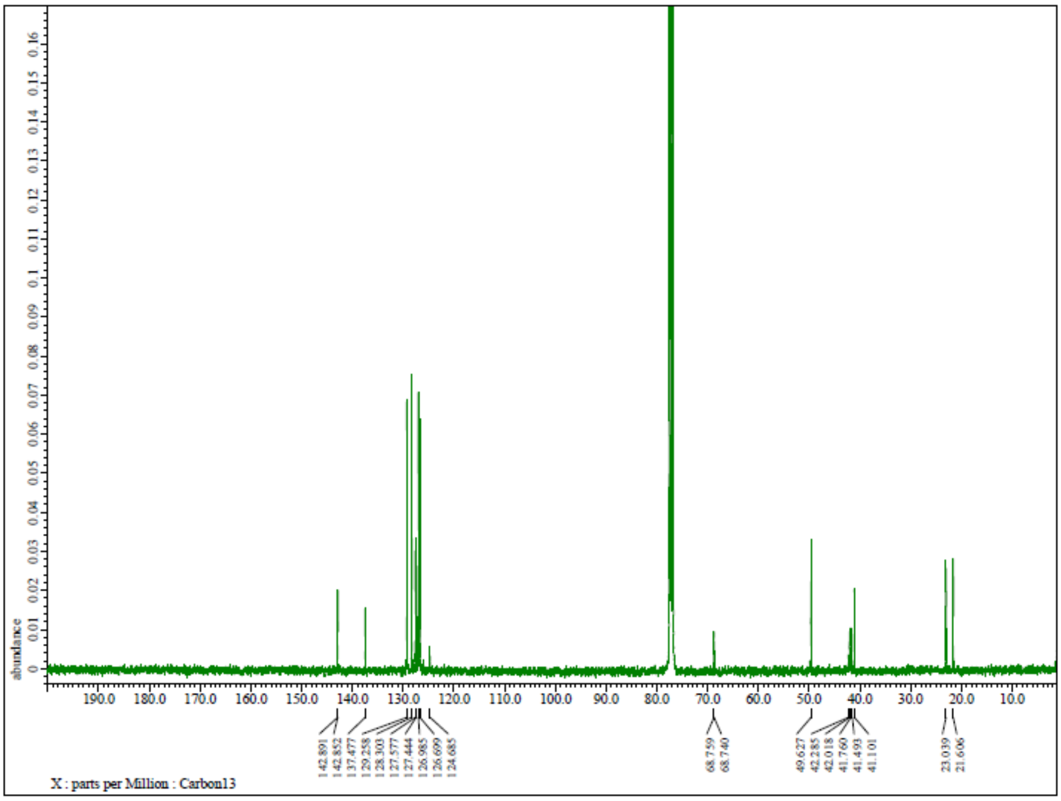
abundance
X : parts per Million : Carbon13
190.0
180.0
170.0
160.0
150.0
140.0
130.0
120.0
110.0
100.0
90.0
80.0
70.0
60.0
50.0
40.0
30.0
20.0
10.0
142.891
142.852
137.477
129.258
128.303
127.577
127.444
126.985
126.699
124.685
68.759
68.740
49.627
42.285
42.018
41.760
41.493
41.101
23.039
21.606

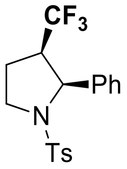
CF3
Ph
N
Ts

(**4b**)

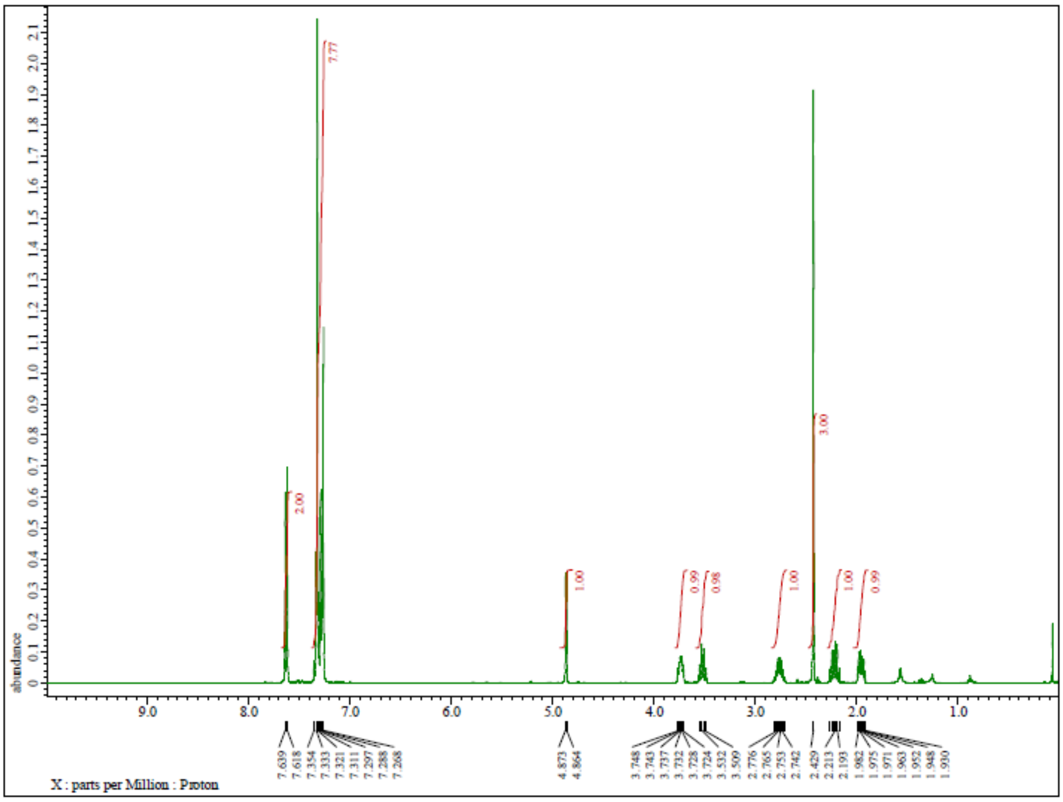
abundance
X : parts per Million : Proton
2.00
7.77
1.00
0.99
0.98
1.00
3.00
1.00
0.99
7.639
7.618
7.354
7.333
7.321
7.311
7.297
7.288
7.268
4.873
4.864
3.748
3.743
3.737
3.732
3.728
3.724
3.532
3.509
2.776
2.765
2.753
2.742
2.429
2.213
2.193
1.982
1.975
1.971
1.963
1.952
1.948
1.930

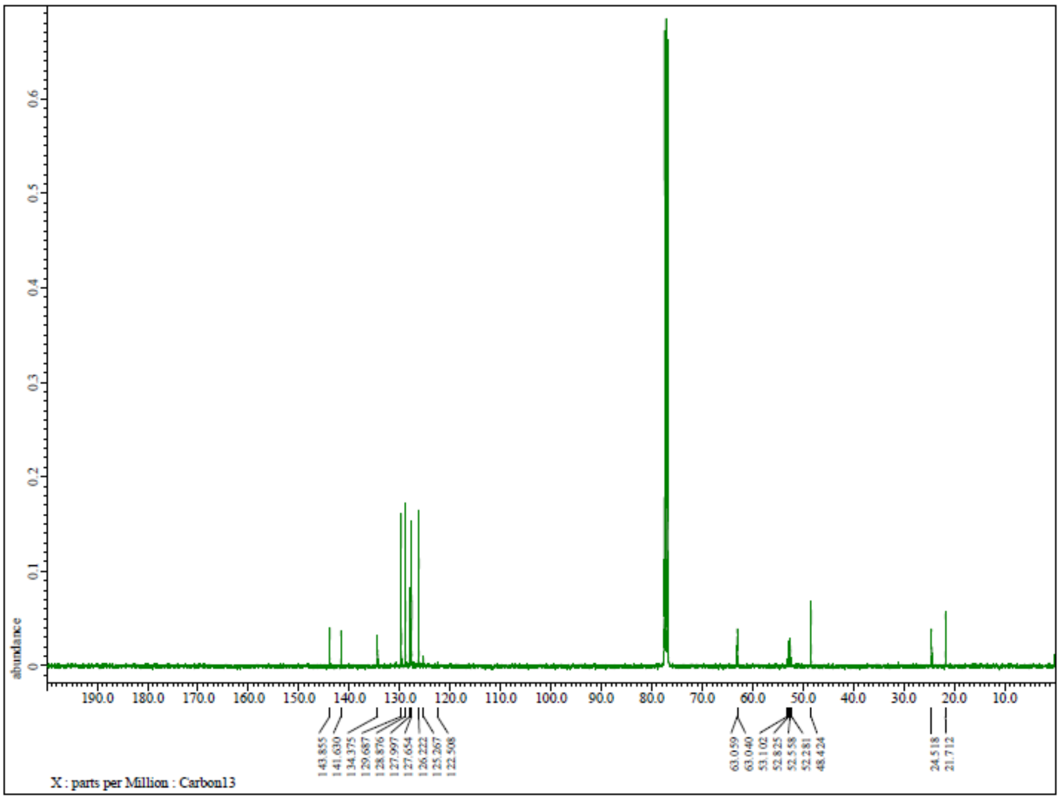
abundance
X : parts per Million : Carbon13
143.855
141.630
134.375
129.687
128.876
127.997
127.654
126.222
125.267
122.508
63.059
63.040
53.102
52.825
52.558
52.281
48.424
24.518
21.712

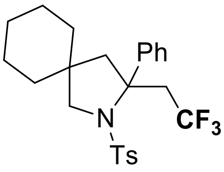
Ph
N
Ts
CF3

(**4c**)

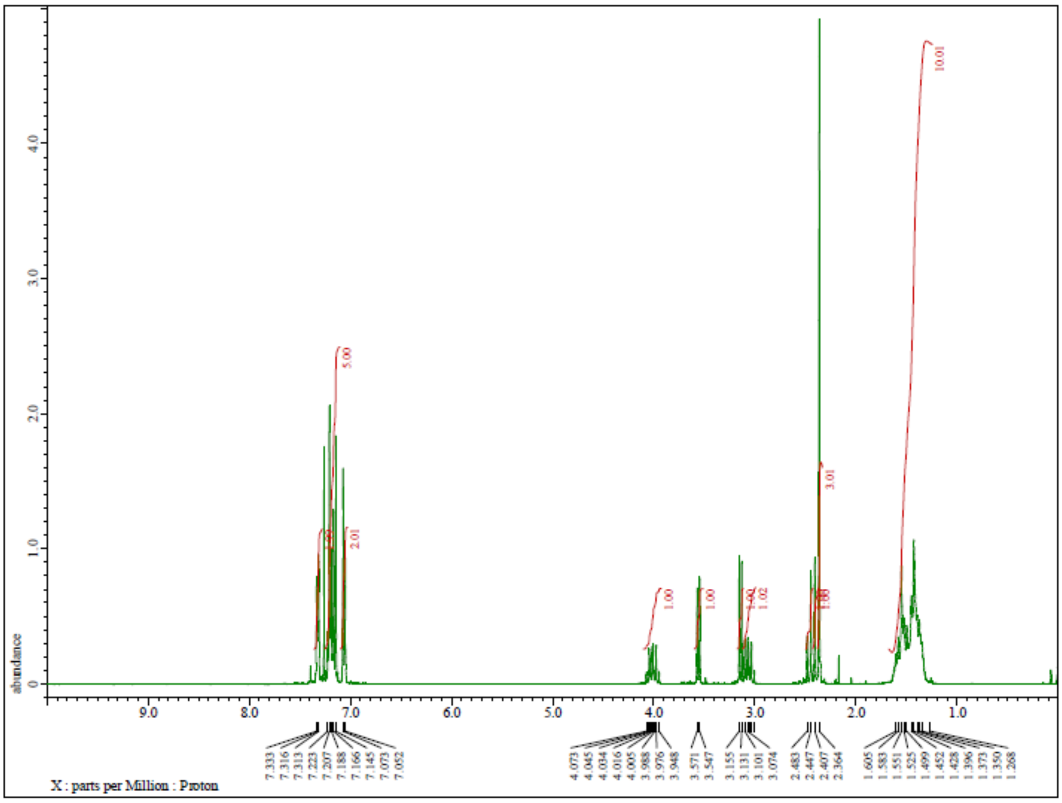
X : parts per Million : Proton
abundance
7.333
7.316
7.313
7.223
7.207
7.188
7.166
7.145
7.073
7.052
4.073
4.045
4.034
4.016
4.005
3.988
3.976
3.948
3.571
3.547
3.155
3.131
3.101
3.074
2.483
2.447
2.407
2.364
1.605
1.583
1.551
1.525
1.499
1.452
1.428
1.396
1.373
1.350
1.268
5.00
2.01
1.00
1.00
1.00
1.02
3.01
10.01

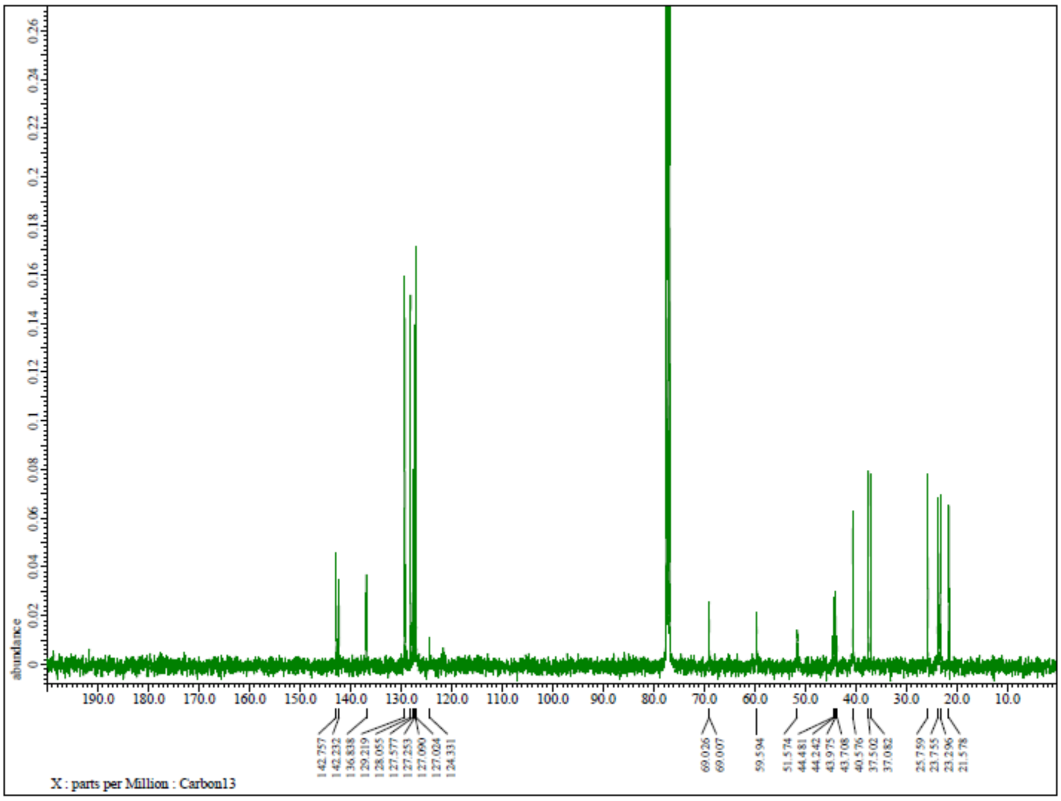
X : parts per Million : Carbon13
abundance
142.757
142.232
136.838
129.219
128.055
127.577
127.253
127.090
127.024
124.331
69.026
69.007
59.594
51.574
44.481
44.242
43.975
43.708
40.576
37.502
37.082
25.759
23.755
23.296
21.578

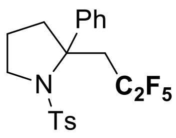
Ph
N
Ts
C2F5

(4a')

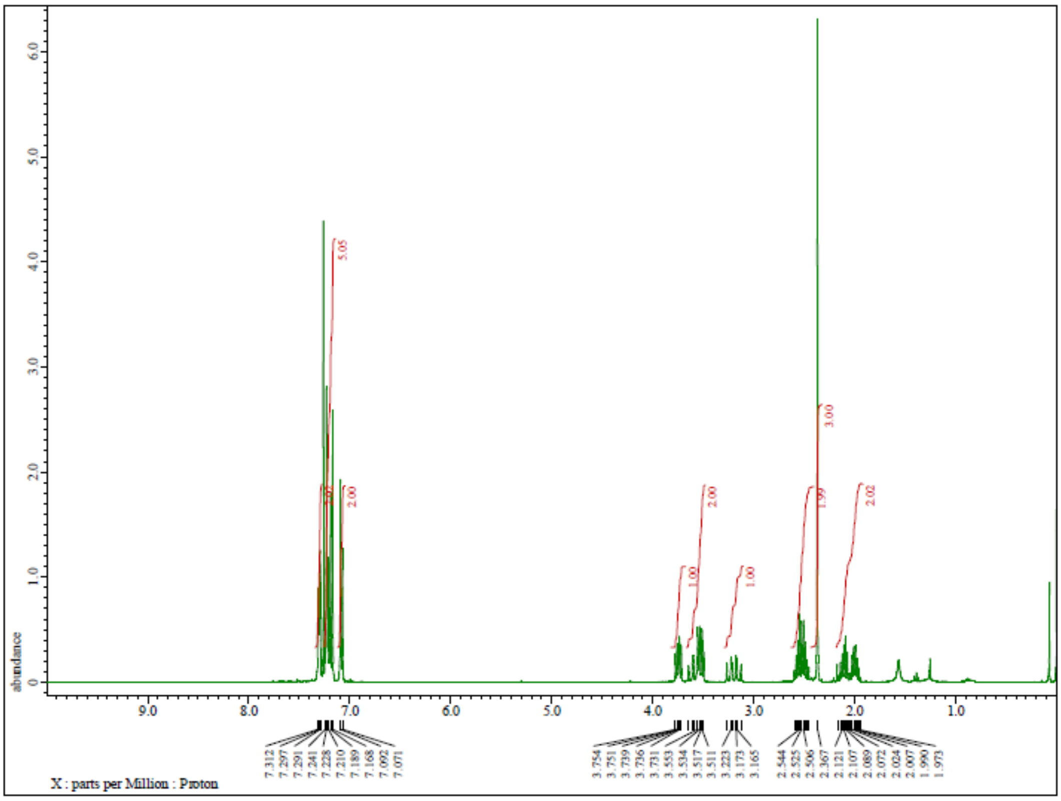
abundance
X : parts per Million : Proton

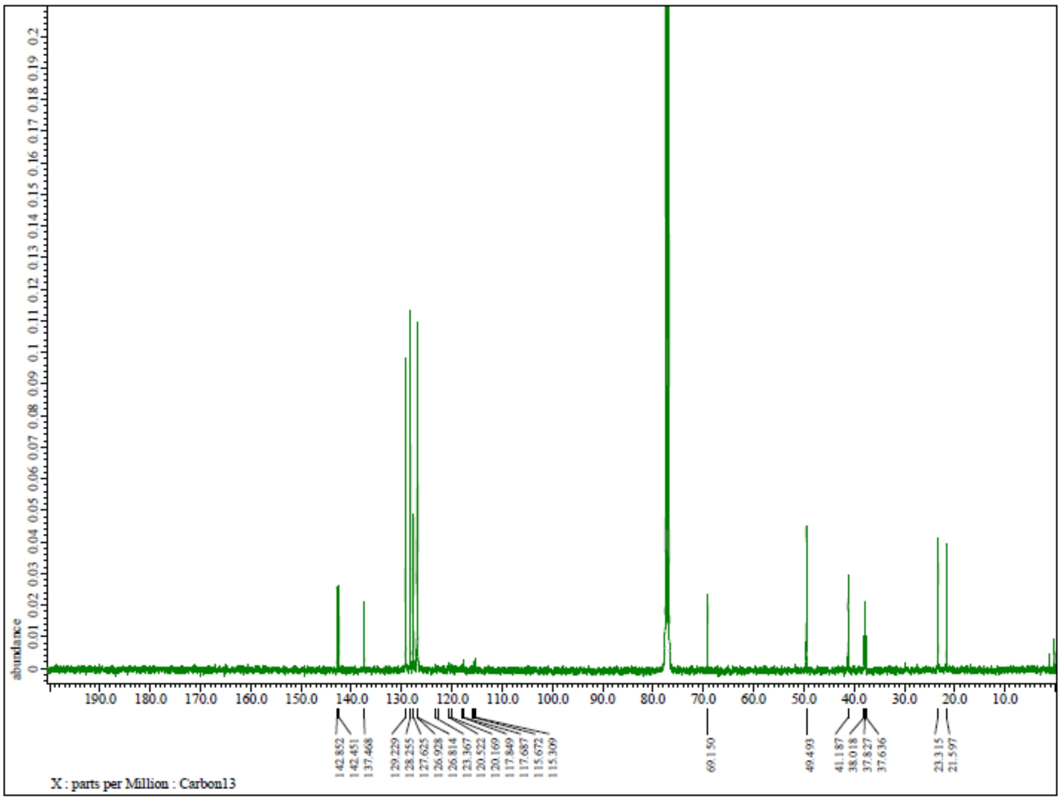
abundance
X : parts per Million : Carbon13

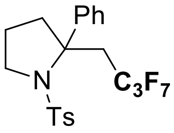

(**4a"**)

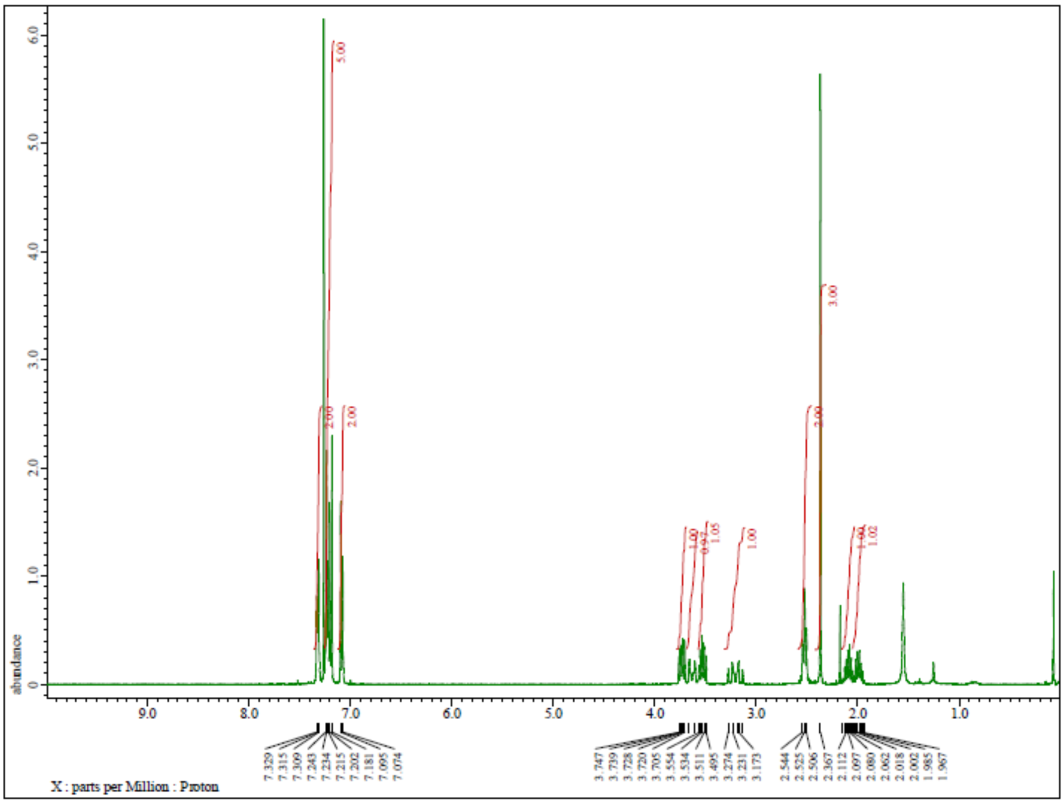

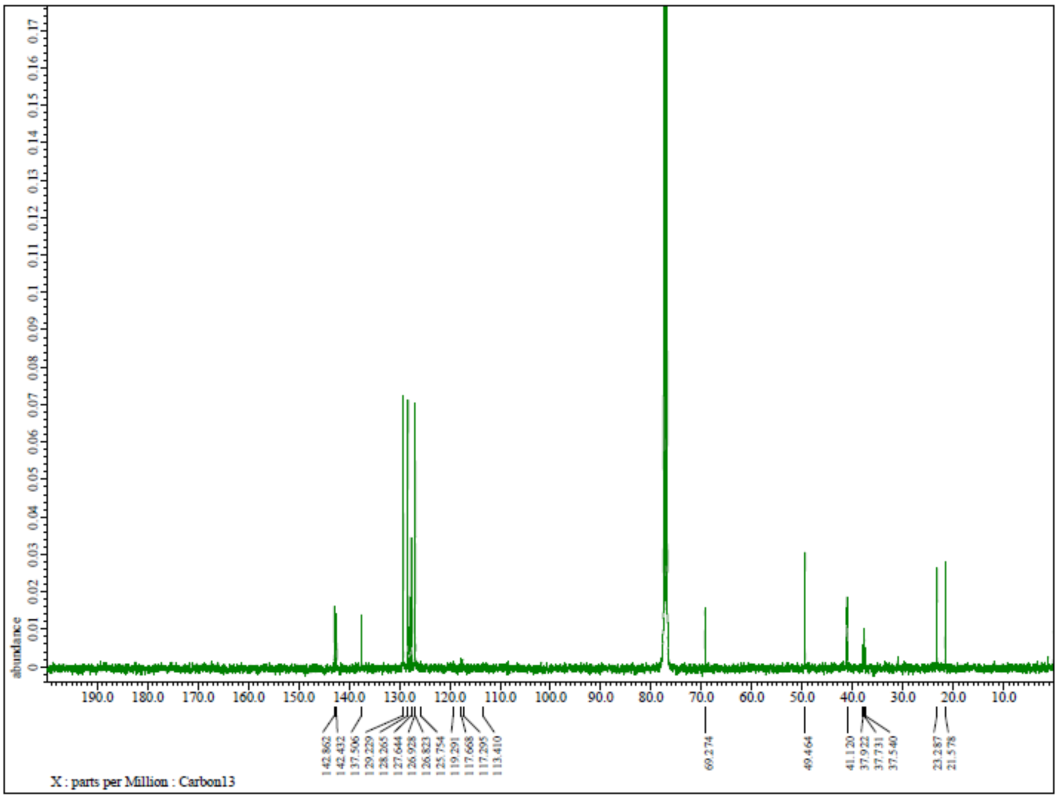

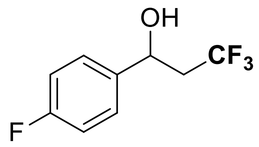
OH
CF3
F

(**5b**)

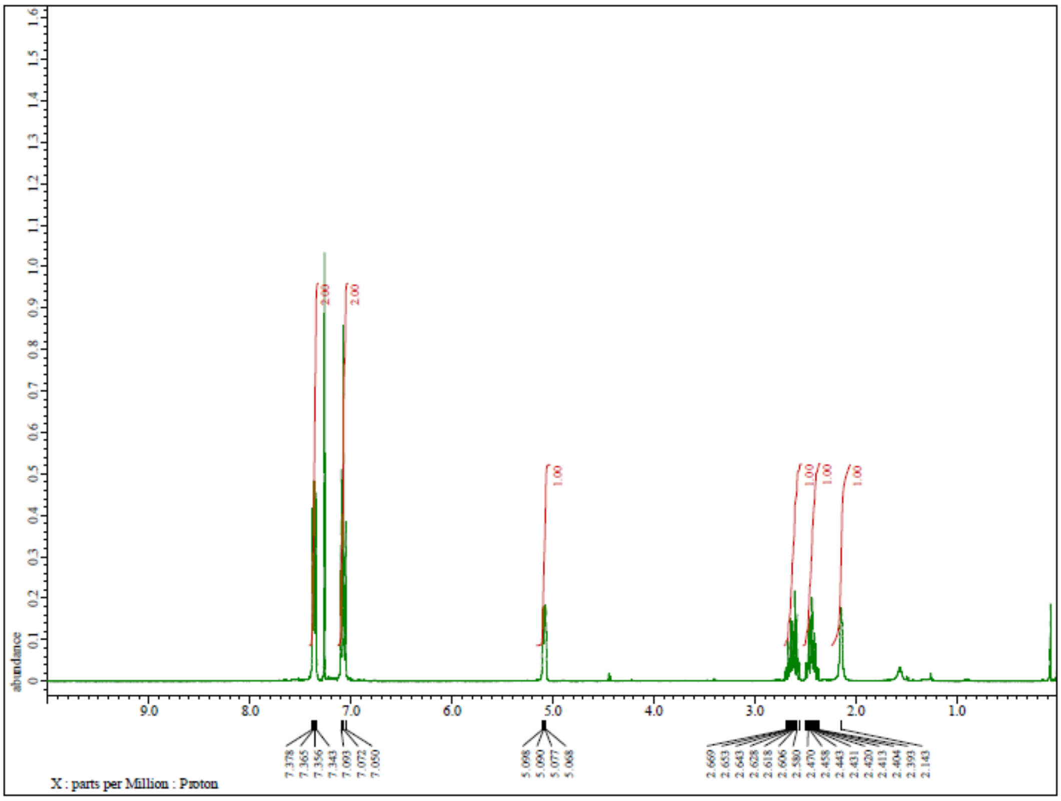
X : parts per Million : Proton
abundance
7.378
7.365
7.356
7.343
7.093
7.072
7.050
5.098
5.090
5.077
5.068
2.669
2.653
2.643
2.628
2.618
2.606
2.580
2.470
2.458
2.443
2.431
2.420
2.413
2.404
2.393
2.143

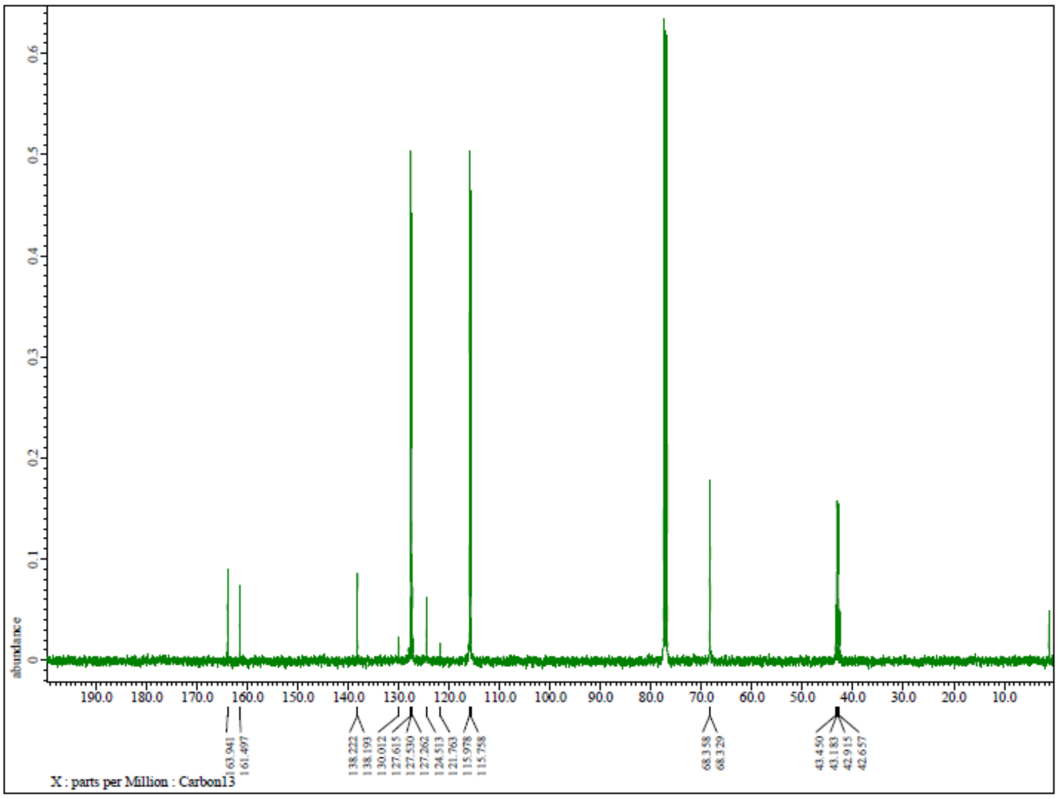
X : parts per Million : Carbon13
abundance
163.941
161.497
138.222
138.193
130.012
127.615
127.530
127.262
124.513
121.763
115.978
115.758
68.358
68.329
43.450
43.183
42.915
42.657

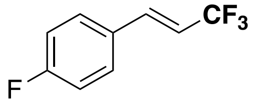
CF3
F

(**6b**)

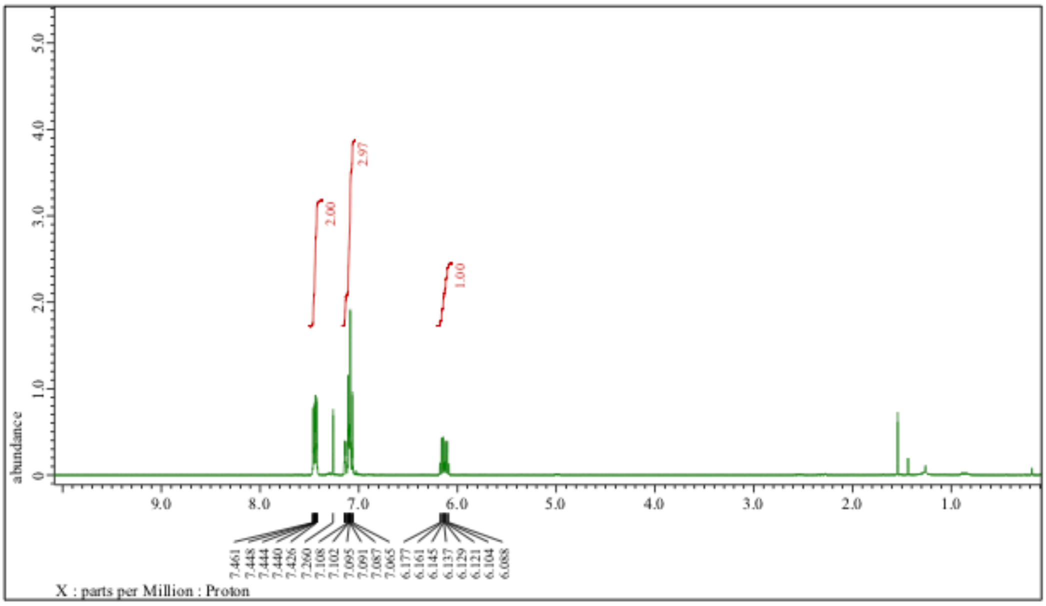
abundance
2.00
2.97
1.00
9.0
8.0
7.0
6.0
5.0
4.0
3.0
2.0
1.0
7.461
7.448
7.444
7.440
7.426
7.260
7.108
7.102
7.095
7.091
7.087
7.065
6.177
6.161
6.145
6.137
6.129
6.121
6.104
6.088
X : parts per Million : Proton

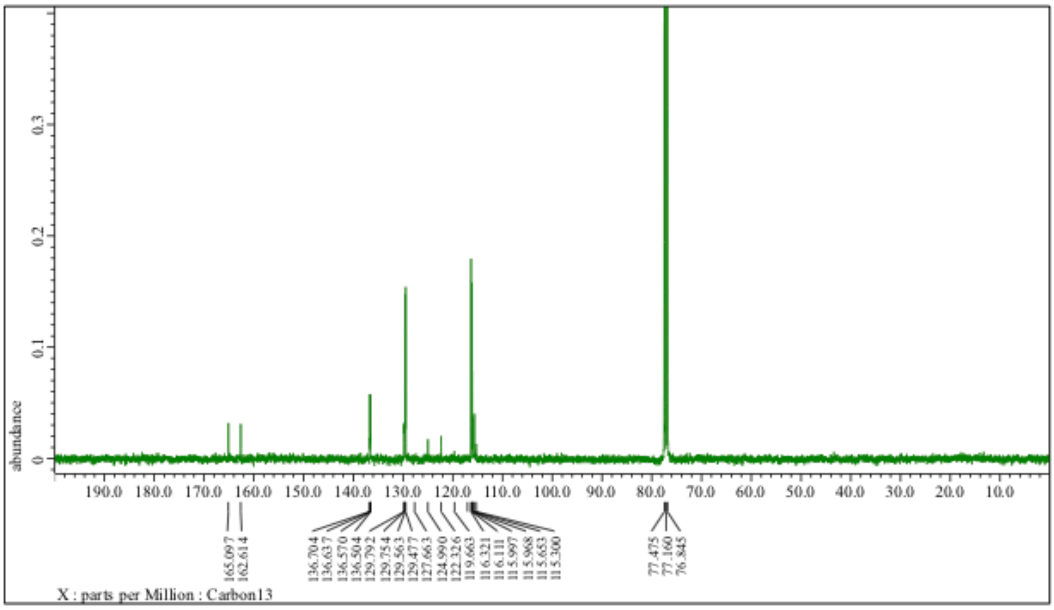
abundance
190.0
180.0
170.0
160.0
150.0
140.0
130.0
120.0
110.0
100.0
90.0
80.0
70.0
60.0
50.0
40.0
30.0
20.0
10.0
165.097
162.614
136.704
136.637
136.570
136.504
129.792
129.754
129.563
129.477
127.663
124.990
122.326
119.663
116.321
116.111
115.997
115.968
115.653
115.300
77.475
77.160
76.845
X : parts per Million : Carbon13

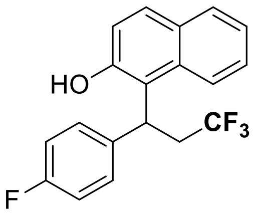
HO
CF3
F

(**7b**)

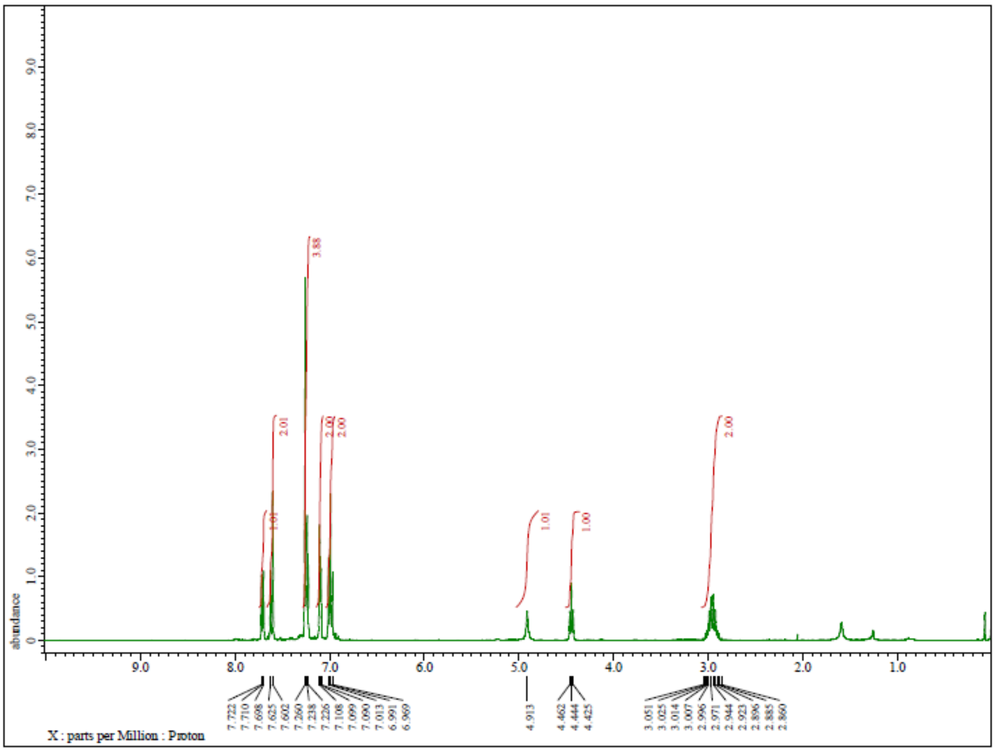
abundance
X : parts per Million : Proton

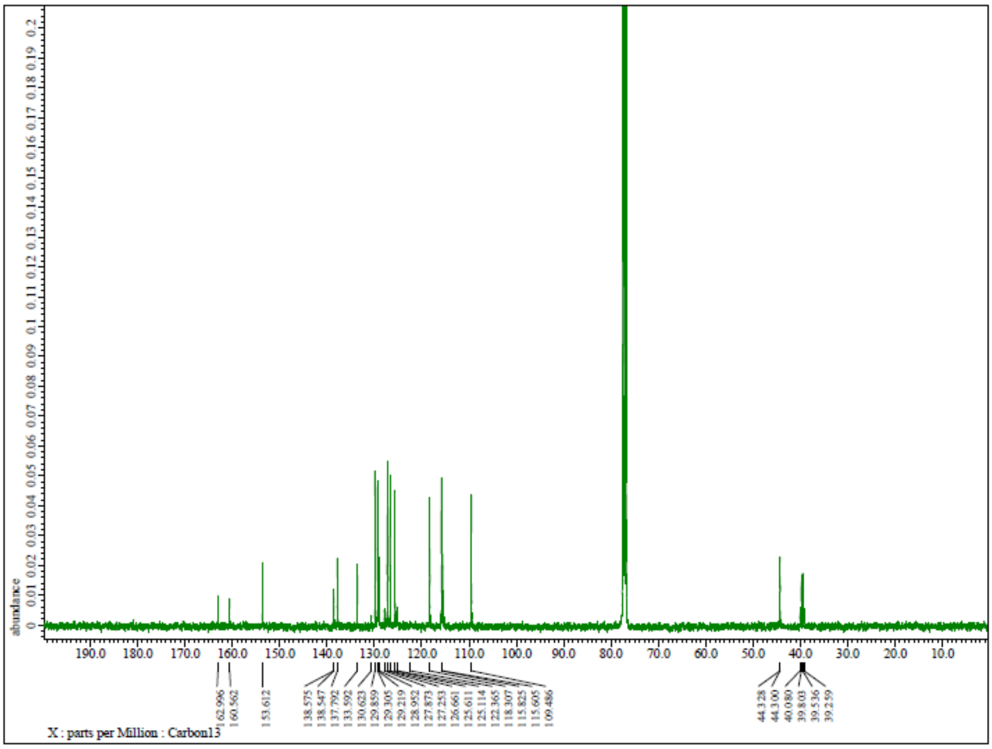
abundance
X : parts per Million : Carbon13

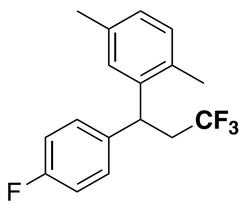
CF3
F

(**8b**)

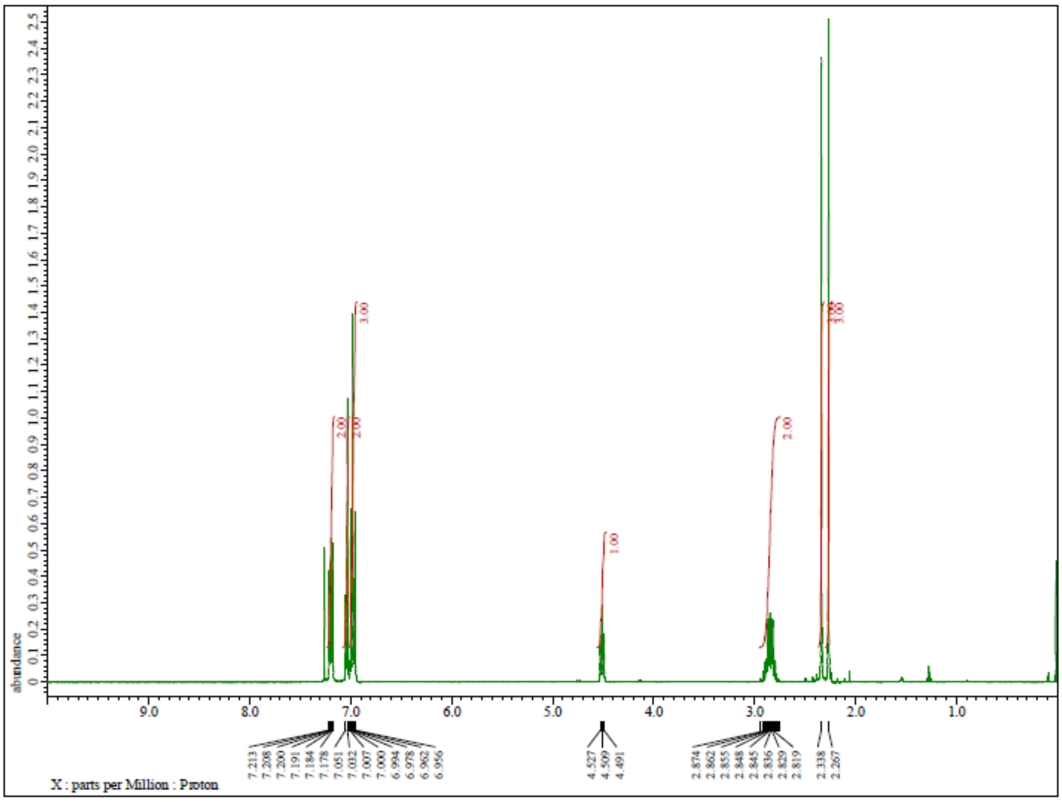
X : parts per Million : Proton
abundance

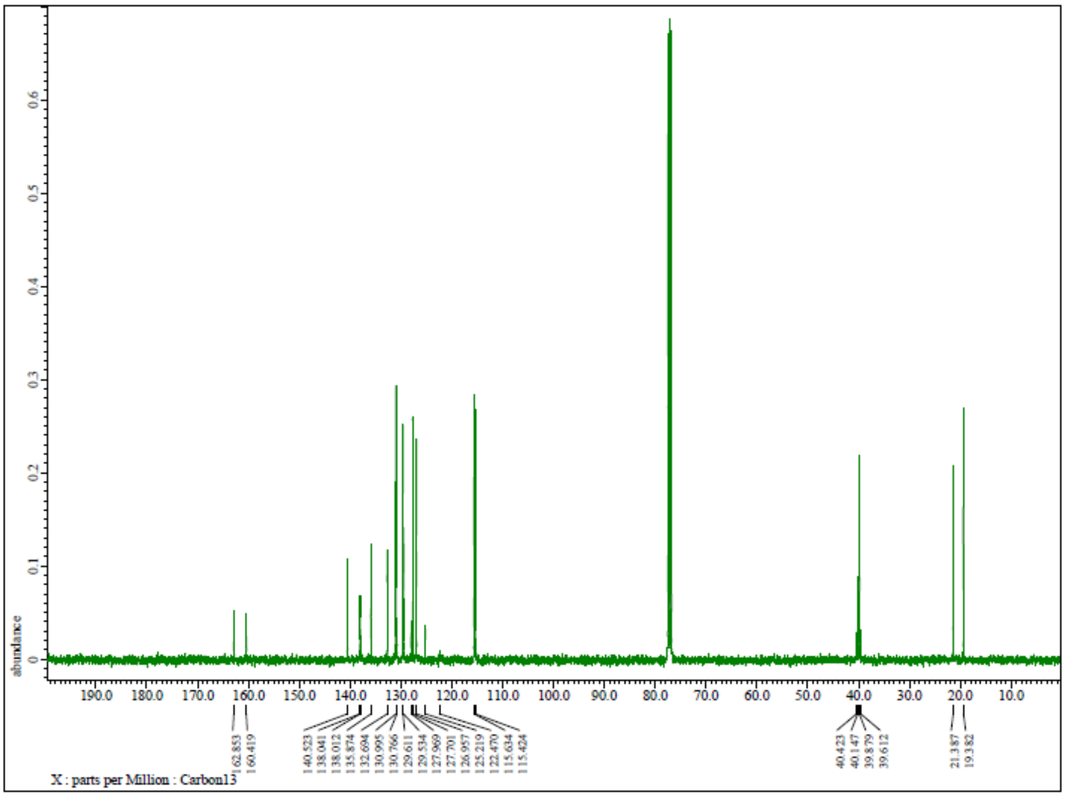
X : parts per Million : Carbon13
abundance

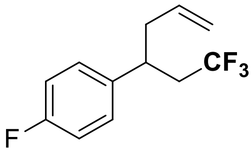

(**9b**)

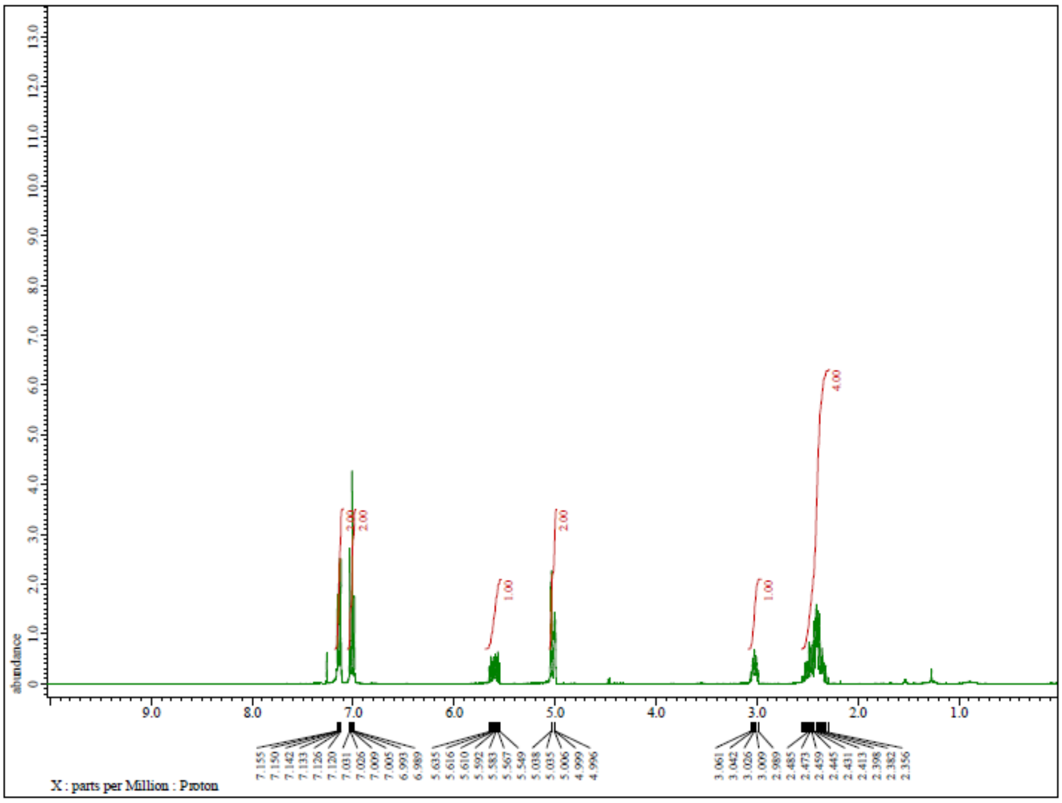
X : parts per Million : Proton
abundance

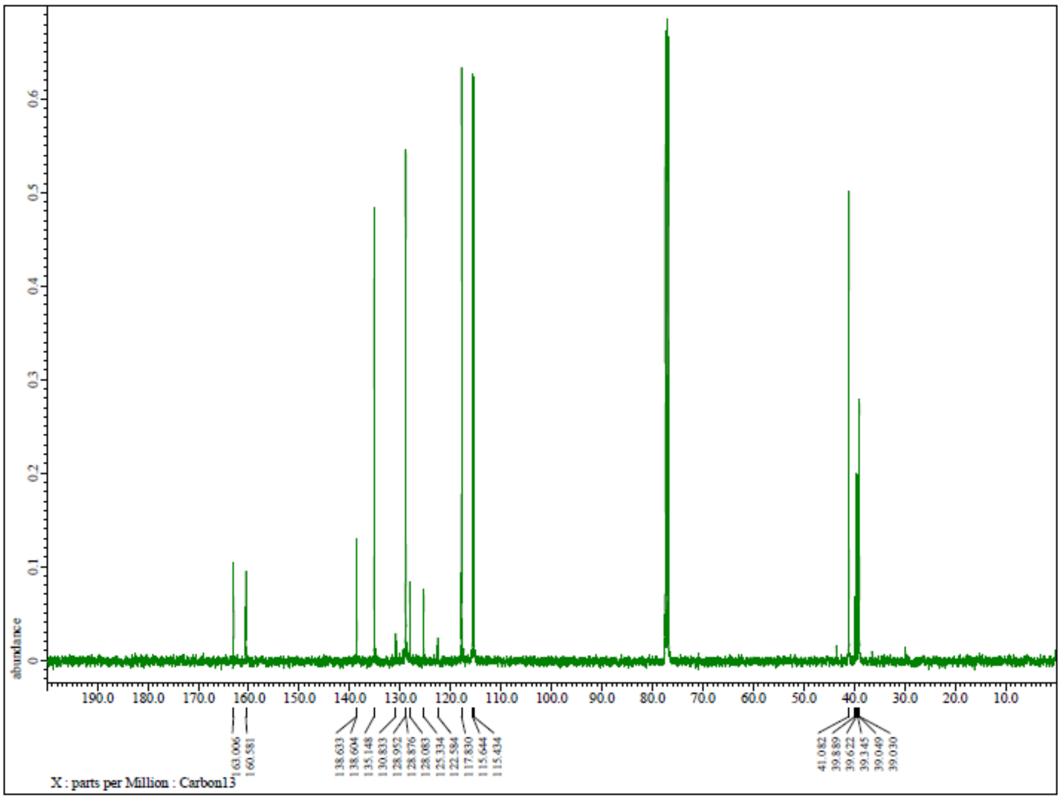
X : parts per Million : Carbon13
abundance

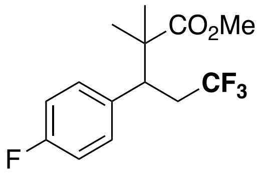

CO2Me
CF3
F

(**10b**)

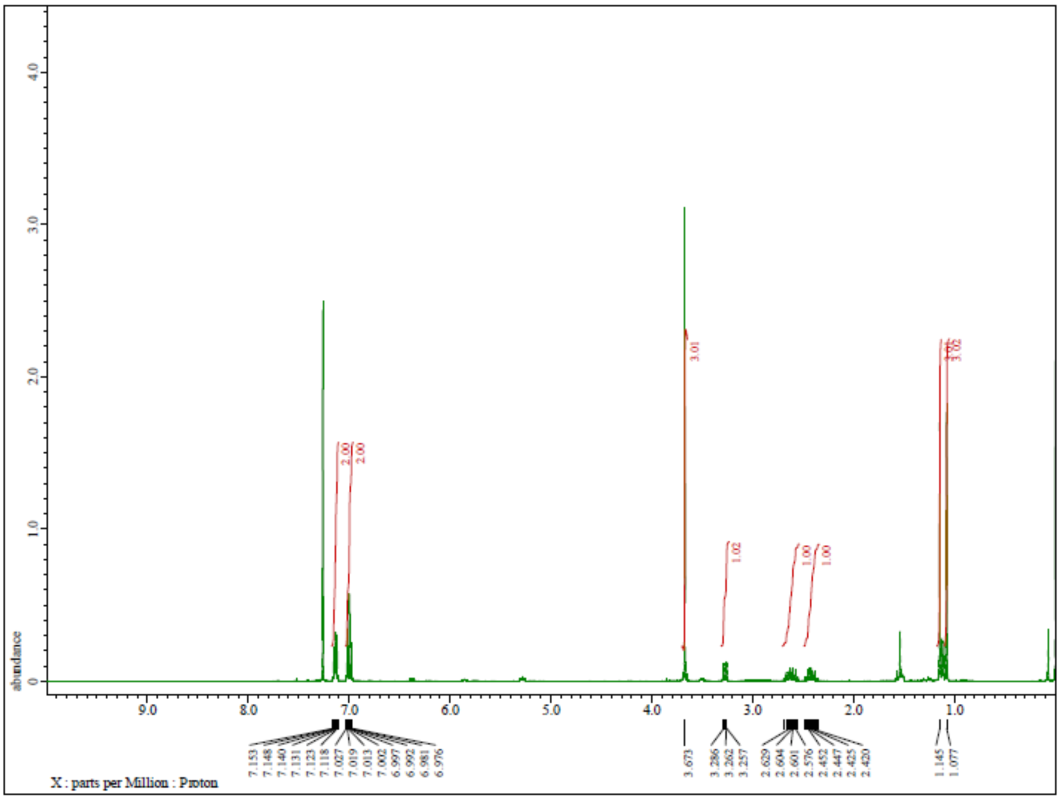

abundance
4.0
3.0
2.0
1.0
0
9.0
8.0
7.0
6.0
5.0
4.0
3.0
2.0
1.0
2.00
2.00
3.01
1.02
1.00
1.00
3.02
7.153
7.148
7.140
7.131
7.123
7.118
7.027
7.019
7.013
7.002
6.997
6.992
6.981
6.976
3.673
3.286
3.262
3.257
2.629
2.604
2.601
2.576
2.452
2.447
2.425
2.420
1.145
1.077
X : parts per Million : Proton

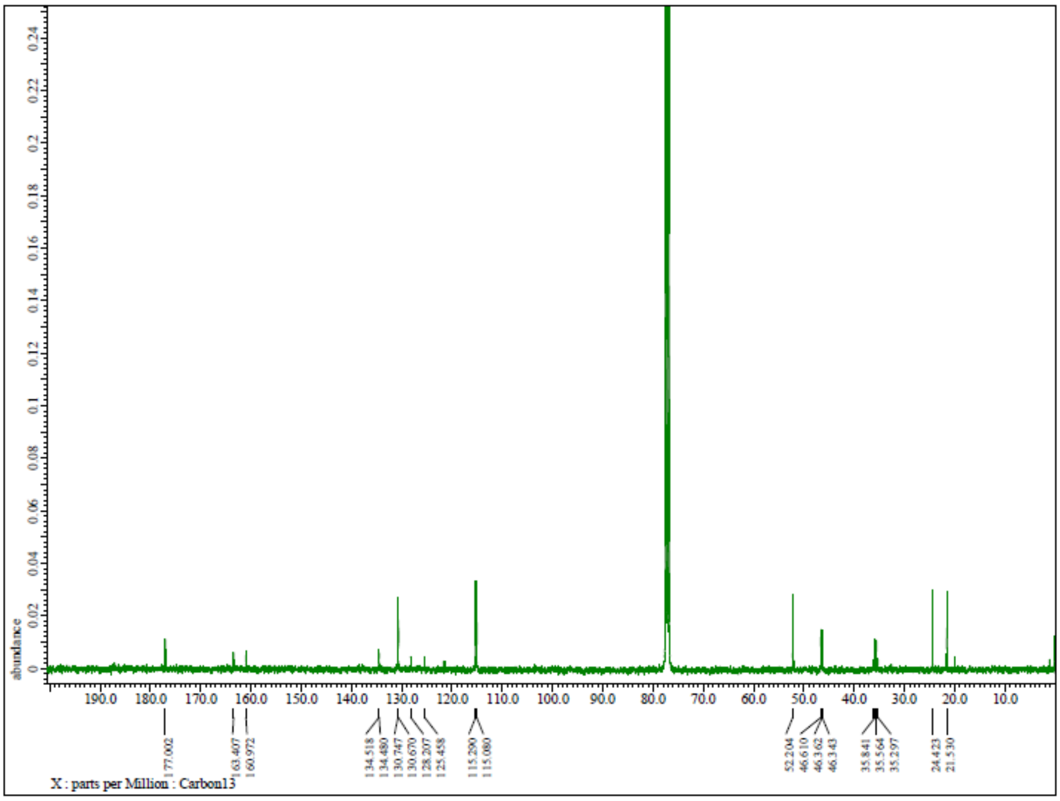

abundance
0.24
0.22
0.2
0.18
0.16
0.14
0.12
0.1
0.08
0.06
0.04
0.02
0
190.0
180.0
170.0
160.0
150.0
140.0
130.0
120.0
110.0
100.0
90.0
80.0
70.0
60.0
50.0
40.0
30.0
20.0
10.0
177.002
163.407
160.972
134.518
134.480
130.747
130.670
128.207
125.458
115.290
115.080
52.204
46.610
46.362
46.343
35.841
35.564
35.297
24.423
21.530
X : parts per Million : Carbon13

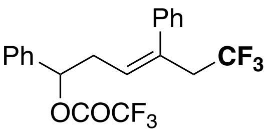

(**13**)

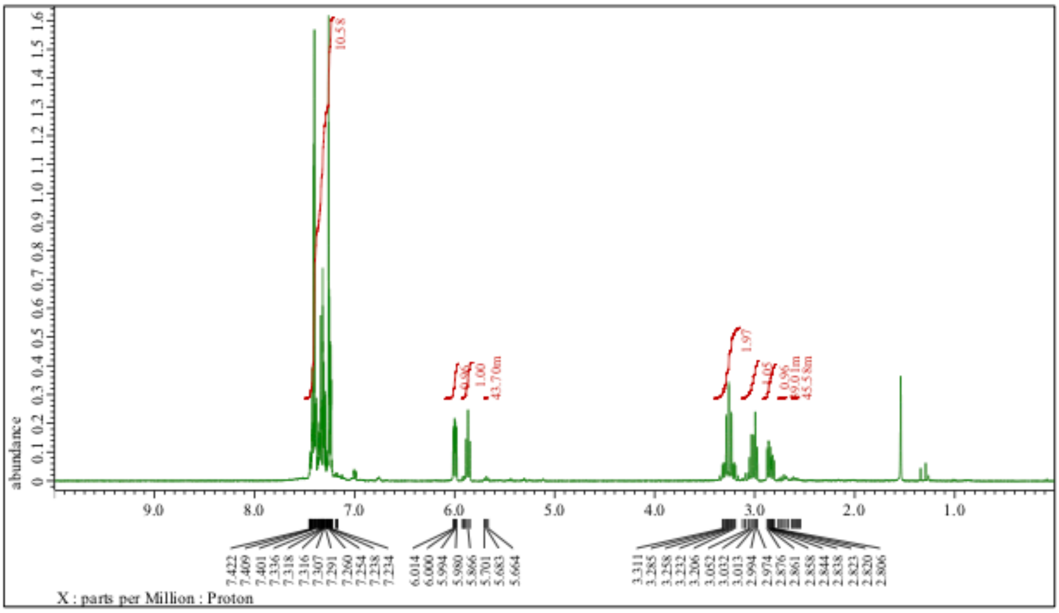

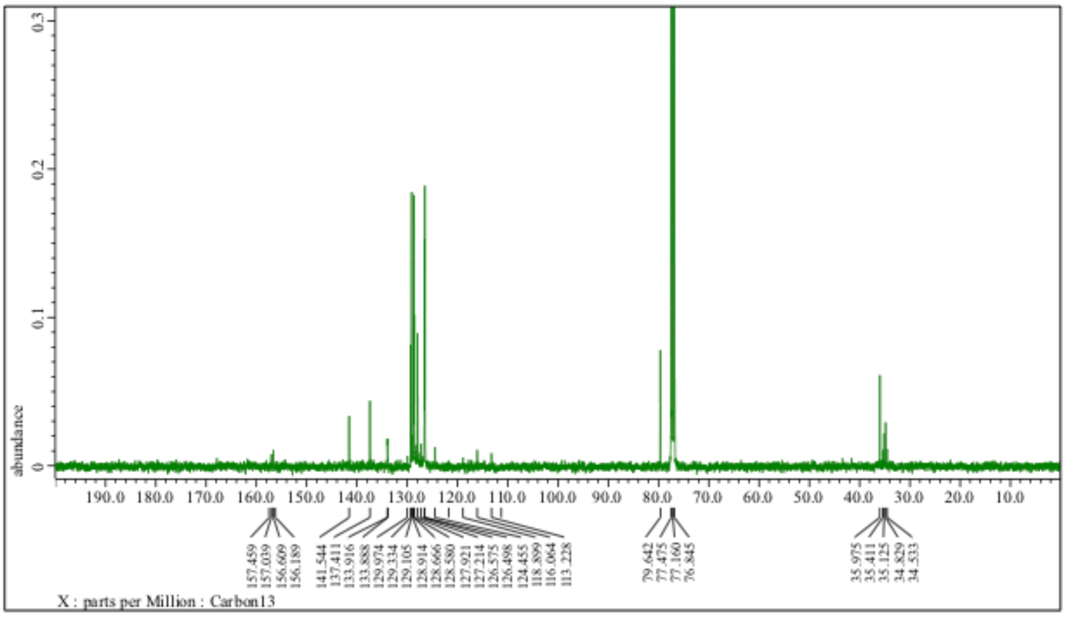

5. Computational details:

DFT calculations were conducted with Gaussian 16 series[13] of programs. The structures were optimized at the UB3LYP level of theory, and 6-31+G(d,p) basis set was used. The single-point energy calculation was performed using UMPWB1K/6-311+G(2df,2p), except that LanL2DZ was used for Cu. The CPCM solvation model (dichloromethane) was used to reflect the solvent effect. The free energies described in this work were estimated from the ZPEs from UMPWB1K with thermal corrections by using vibrational analysis at the UB3LYP level of theory. No imaginary frequencies for intermediates and one imaginary frequency for the transition state were observed. The reaction pathway from the transition state was confirmed by IRC calculation and the vibration mode of the imaginary frequency. DFT calculations were conducted according to the literature procedure reported by Houk and Buchwald.[15] The activation energy ($\Delta G^{\ddagger}$) of SET was estimated according to the Marcus equation with parameters as shown in Scheme S1 (n = 1.424, ε= 8.93 for CH_2Cl_2).

$$\Delta^{\ddagger}G = \left(\lambda/_4\right)\left(1 + {}^{\Delta G}/_{\lambda}\right)^2$$

$$\lambda \approx 332 \times \left(\frac{1}{2r_A} + \frac{1}{2r_B} - \frac{1}{R}\right)\left(\frac{1}{n^2} - \frac{1}{\varepsilon}\right)$$; reorganization energy (kcal/mol)

ΔG; reaction energy (kcal/mol)

r; radius of molecules (Å)

$R = r_A + r_B$ (Å)

n; index of refraction

ε; dielectric constant

---

[13] M. J. Frisch, G. W. Trucks, H. B. Schlegel, G. E. Scuseria, M. A. Robb, J. R. Cheeseman, G. Scalmani, V. Barone, B. Mennucci, G. A. Petersson, H. Nakatsuji, M. Caricato, X. Li, H. P. Hratchian, A. F. Izmaylov, J. Bloino, G. Zheng, J. L. Sonnenberg, M. Hada, M. Ehara, K. Toyota, R. Fukuda, J. Hasegawa, M. Ishida, T. Nakajima, Y. Honda, O. Kitao, H. Nakai, T. Vreven, J. A. Montgomery, Jr., J. E. Peralta, F. Ogliaro, M. Bearpark, J. J. Heyd, E. Brothers, K. N. Kudin, V. N. Staroverov, R. Kobayashi, J. Normand, K. Raghavachari, A. Rendell, J. C. Burant, S. S. Iyengar, J. Tomasi, M. Cossi, N. Rega, J. M. Millam, M. Klene, J. E. Knox, J. B. Cross, V. Bakken, C. Adamo, J. Jaramillo, R. Gomperts, R. E. Stratmann, O. Yazyev, A. J. Austin, R. Cammi, C. Pomelli, J. W. Ochterski, R. L. Martin, K. Morokuma, V. G. Zakrzewski, G. A. Voth, P. Salvador, J. J. Dannenberg, S. Dapprich, A. D. Daniels, O. Farkas, J. B. Foresman, J. V. Ortiz, J. Cioslowski, and D. J. Fox, *Gaussian 09,* Revision A.02; Gaussian, Inc.: Wallingford CT, 2009.

[15] G. O. Jones, P. Liu, K. N. Houk, S. L. Buchwald, *J. Am. Chem. Soc.* **2010**, *132*, 6205.

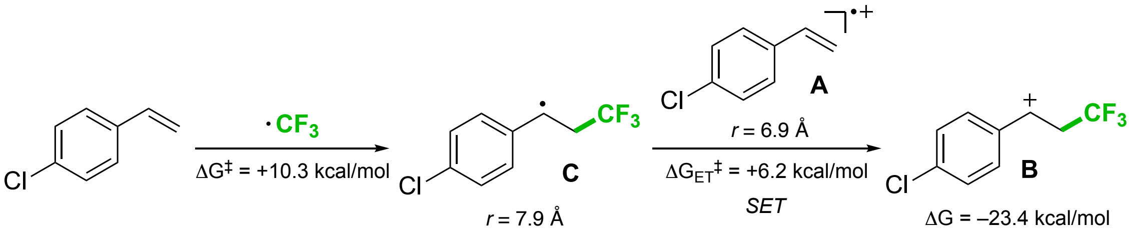

Scheme S1. Estimation of activation energies of pathway b

HOMO and LUMO levels

Table S2. HOMO levels of styrenes

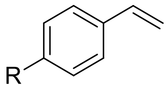

R	HOMO (eV)
OMe	-5.90
Me	-6.20
Cl	-6.41
CF_3	-6.74
Benzene	-7.90

Table S3. LUMO levels of potential oxidants

Oxidant	LUMO (eV)
BTFAP	-2.35
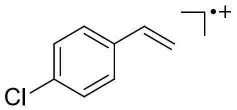	-6.26
$Cu^{II}(O_2CCF_3)_2$	-5.09

Cartesian coordinates and energies

$\cdot CF_3$

E (UMPWB1K) = -337.5780555
Sum electronic and thermal free energies =
-337.5930245
Charge = 0 Multiplicity = 2

C	0.00027	0.00002	0.32655
F	-0.97128	0.80219	-0.07254
F	-0.20919	-1.24218	-0.07256
F	1.18029	0.43998	-0.0726

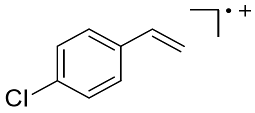

A

E (UMPWB1K) = -769.0158561
Sum electronic and thermal free energies =
-768.9261741
Charge = 1 Multiplicity = 2

C	1.42252	0.23852	0.00001
C	0.57961	1.39434	0.
C	-0.78631	1.27451	0.
C	-1.3553	-0.01927	0.
C	-0.55418	-1.18497	0.00001
C	0.80961	-1.0561	0.00002
H	1.03617	2.3765	-0.00001
H	-1.42954	2.14396	-0.00001
H	-1.02559	-2.15832	0.00001
H	1.4217	-1.9473	0.00002
C	2.83359	0.43103	0.00001
H	3.1796	1.45969	0.00005
C	3.77812	-0.56229	-0.00003
H	3.52819	-1.61556	-0.00006
H	4.83112	-0.31	-0.00001
Cl	-3.05339	-0.17904	-0.00001

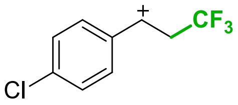

B

E (UMPWB1K) = -1106.7106645
Sum electronic and thermal free energies =
-1106.609694
Charge = 1 Multiplicity = 1

C	-0.29953	0.30696	-0.27609
C	-1.17376	1.4372	-0.11899
C	-2.52621	1.26734	0.02967
C	-3.04496	-0.04189	0.02514
C	-2.22233	-1.18008	-0.12686
C	-0.87142	-1.01036	-0.2733
H	-0.748	2.43311	-0.11909
H	-3.19105	2.1113	0.1505
H	-2.66637	-2.16586	-0.12209
H	-0.23909	-1.88042	-0.38431
C	2.12478	-0.44394	-0.65391
H	2.38109	-0.3976	-1.72298
H	1.84108	-1.46965	-0.42615
C	3.40535	-0.11391	0.11101
F	3.20734	-0.15482	1.44154
F	4.36873	-0.9964	-0.18483
F	3.86597	1.11412	-0.18731
C	1.04941	0.54931	-0.42355
H	1.36743	1.58918	-0.40279
Cl	-4.72774	-0.26529	0.21433

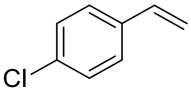

E (UMPWB1K) = -769.2528712

Sum electronic and thermal free energies =
-769.1628782
Charge = 0 Multiplicity = 1

C	1.43138	0.2244	0.
C	0.60427	1.35892	-0.00001
C	-0.7848	1.25226	0.
C	-1.36045	-0.01378	0.
C	-0.57187	-1.16404	-0.00001
C	0.81241	-1.03787	0.
H	1.05481	2.34574	-0.00001
H	-1.40707	2.13801	0.00001
H	-1.0352	-2.14251	0.
H	1.41268	-1.93972	0.
C	2.89089	0.40833	0.
H	3.2127	1.4475	0.
C	3.83258	-0.54305	0.00001
H	3.60103	-1.60251	0.00001
H	4.88375	-0.28021	0.00001
Cl	-3.10878	-0.16926	0.

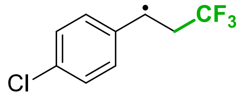

C

E (UMPWB1K) = -1106.9066534
Sum electronic and thermal free energies =
-1106.809183
Charge = 0 Multiplicity = 2

C	-0.32724	0.29685	-0.08624
C	-1.21224	1.41426	-0.04175
C	-2.58472	1.25326	0.00583
C	-3.12201	-0.03893	0.00909
C	-2.29462	-1.16413	-0.03571
C	-0.91979	-0.99918	-0.08256
H	-0.79455	2.41505	-0.04432
H	-3.24024	2.11419	0.04043
H	-2.72835	-2.15615	-0.03425
H	-0.29349	-1.88225	-0.11971
C	2.07819	-0.5953	-0.19484
H	2.07475	-1.09997	-1.17027
H	1.88767	-1.36999	0.55549
C	3.49338	-0.11023	0.0328
F	3.65503	0.46257	1.24566
F	4.37898	-1.12694	-0.03948
F	3.87861	0.80811	-0.88116
C	1.06597	0.50708	-0.1334
H	1.42628	1.52922	-0.15608
Cl	-4.85924	-0.24858	0.06848

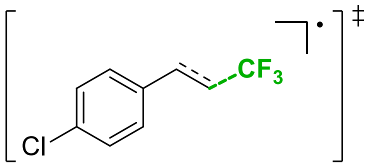

E (UMPWB1K) = -1106.830502
Sum electronic and thermal free energies =
-1106.739551
Charge = 0 Multiplicity = 2

C	0.97264	1.5888	0.42454
H	1.13352	2.45224	-0.2164
C	1.98636	1.20047	1.22651
H	2.89919	1.7809	1.27636
H	1.89439	0.38633	1.93578
C	3.39066	-0.48217	-0.23904
F	4.21128	-1.16372	0.55658
F	4.0917	0.21389	-1.12814
F	2.56251	-1.31362	-0.86046
C	-0.34486	0.96562	0.29283
C	-1.27535	1.52662	-0.5993
C	-0.73695	-0.17386	1.02032
C	-2.54651	0.98426	-0.76578
H	-0.99863	2.40462	-1.17297
C	-2.00117	-0.72823	0.86578
H	-0.05106	-0.63948	1.71748
C	-2.89779	-0.14216	-0.02825
H	-3.25114	1.42911	-1.45659
H	-2.29052	-1.60513	1.43074
Cl	-4.49454	-0.83963	-0.22423
